# Supplementary figures and images for: Human eIF2A has a minimal role in translation initiation and in uORF-mediated translational control in HeLa cells
Source: eLife. 2025 Jul 2;14:RP105311. doi: 10.7554/eLife.105311 (PMC12221301; doi:10.7554/eLife.105311)

Figure 1

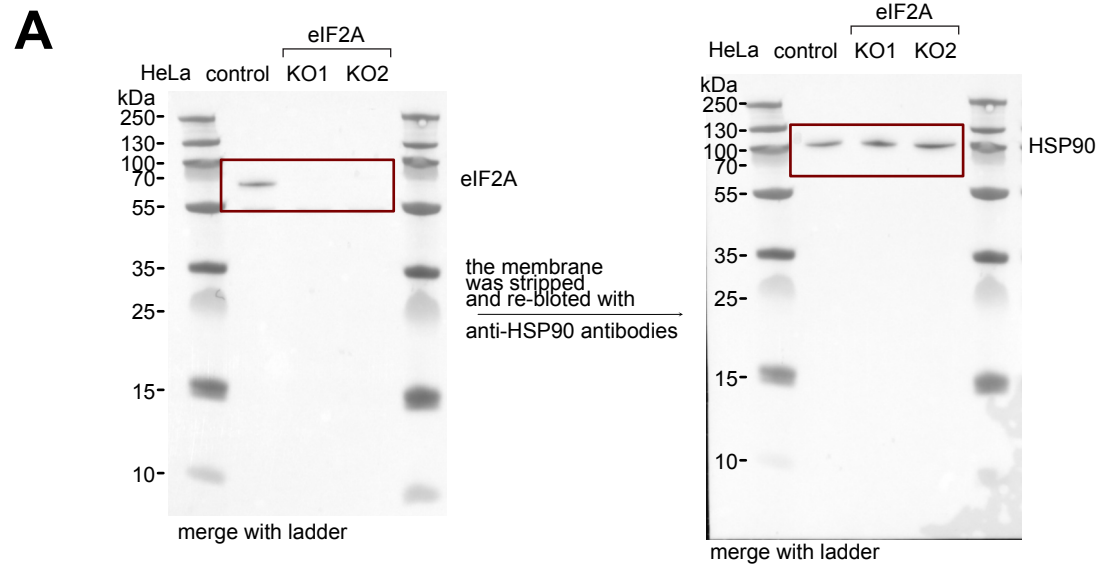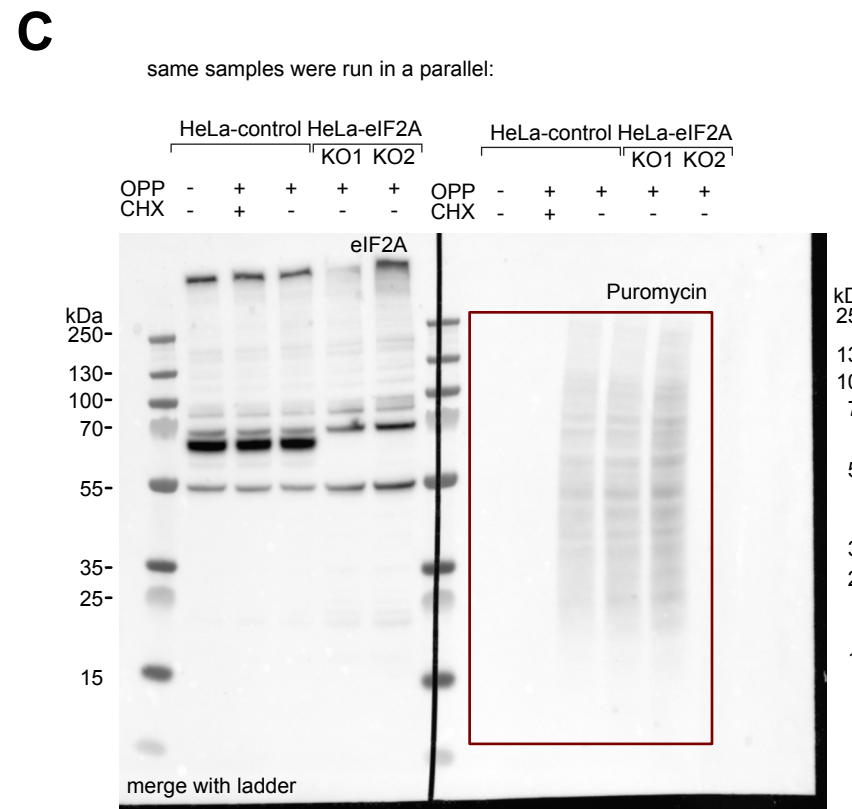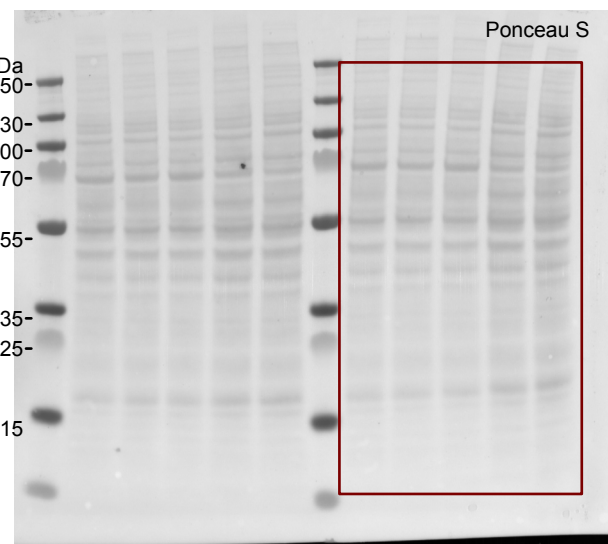

the membranes were stripped and re-blot with anti-HSP90 or eIF2A antibodies

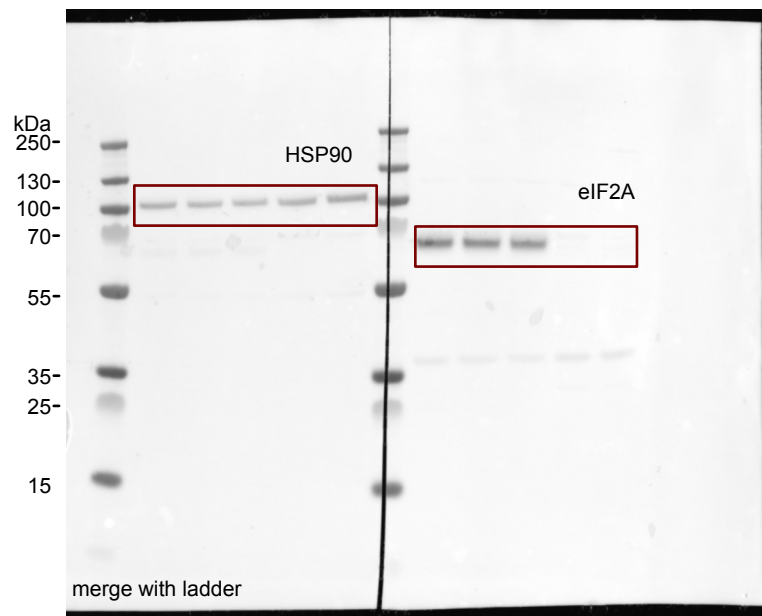

Supplement: Figure 1—source data 1. [file elife-105311-fig1-data1.pdf]

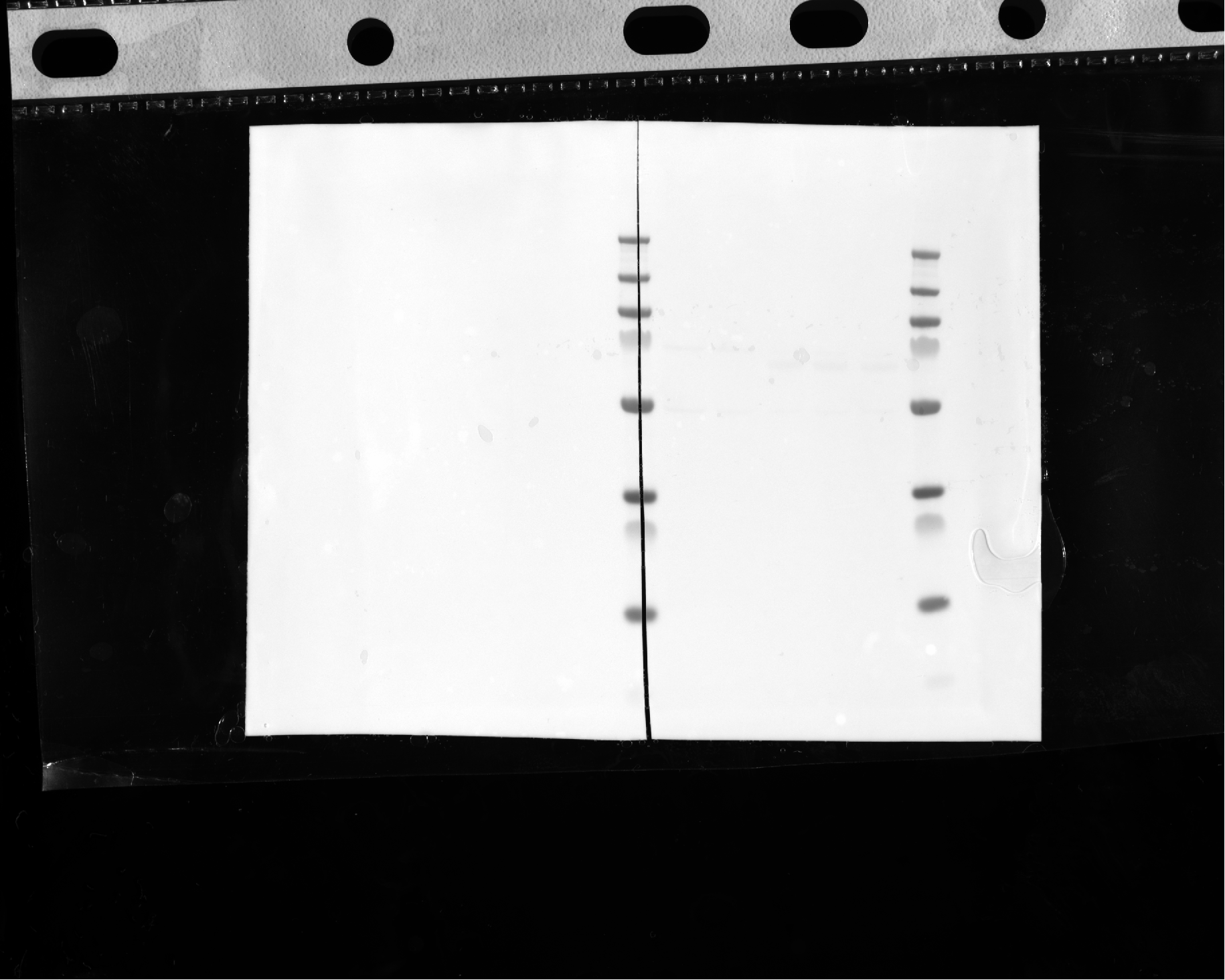

Supplement: Figure 1—source data 2. [file elife-105311-fig1-data2.zip › Figure 1 - Source Data 2/Fig1C_marker_for_HSP90_2023-10-16 13h04m16s 0.161s.tif]

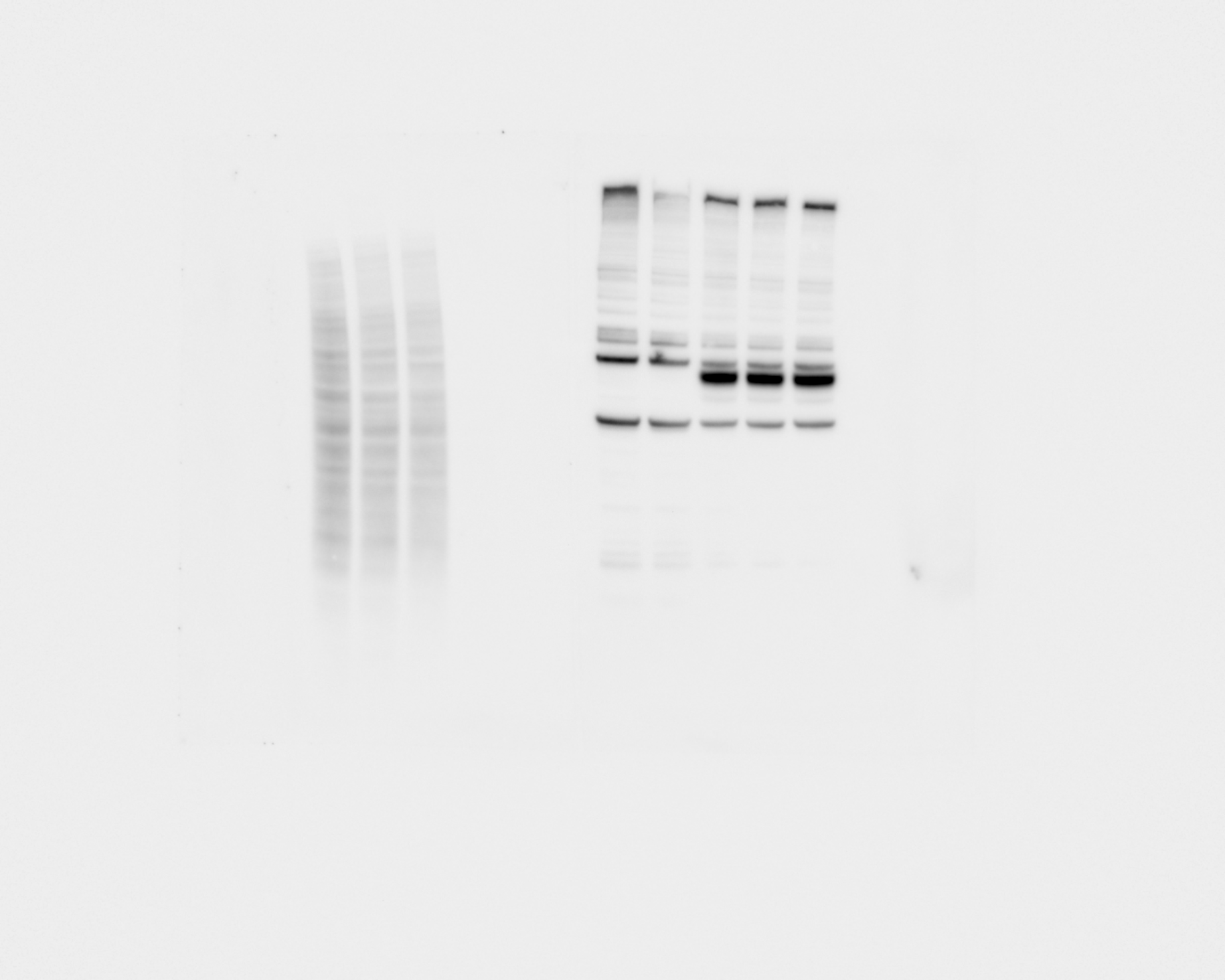

Supplement: Figure 1—source data 2. [file elife-105311-fig1-data2.zip › Figure 1 - Source Data 2/Fig1C_puromycin_eIF2A_2023-10-13 14h10m57s 7.000s.tif]

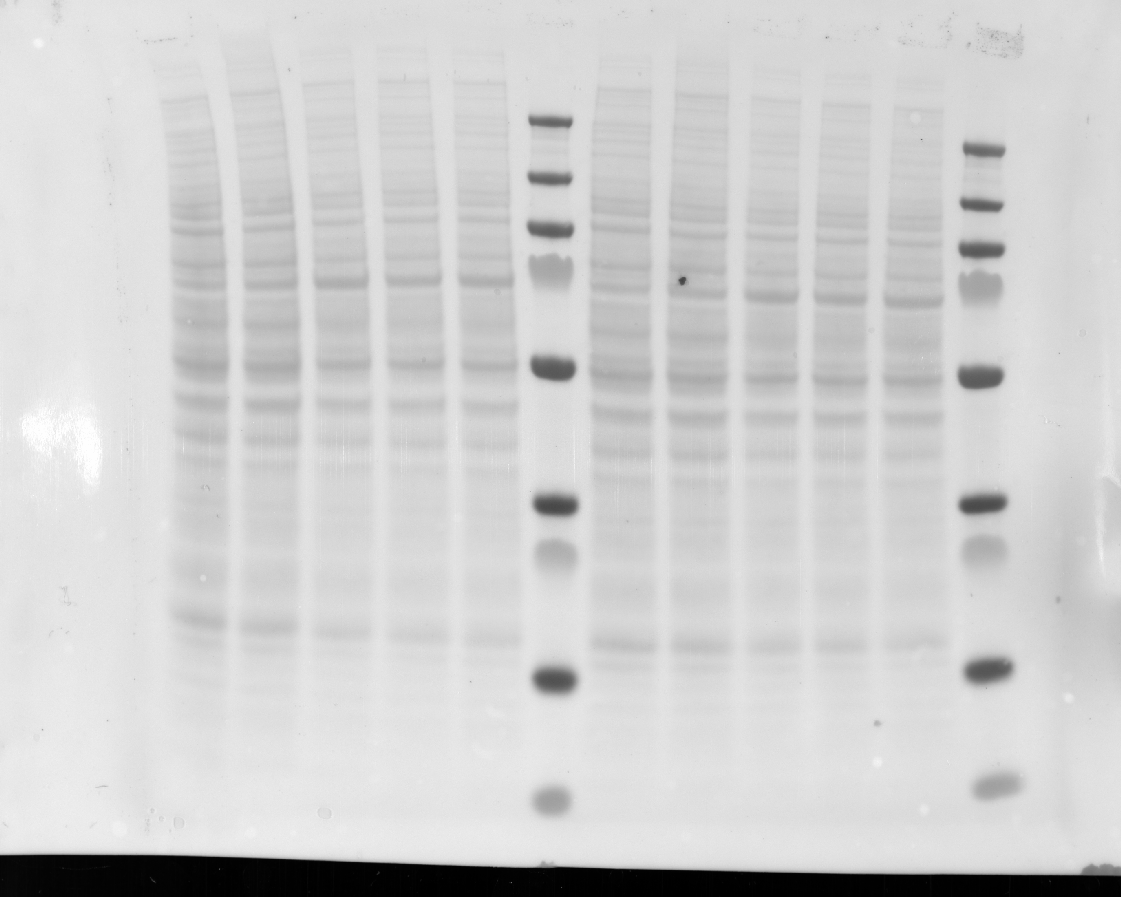

Supplement: Figure 1—source data 2. [file elife-105311-fig1-data2.zip › Figure 1 - Source Data 2/Fig1C_ponceauS_2023-10-12 13h49m08s 0.160s.tif]

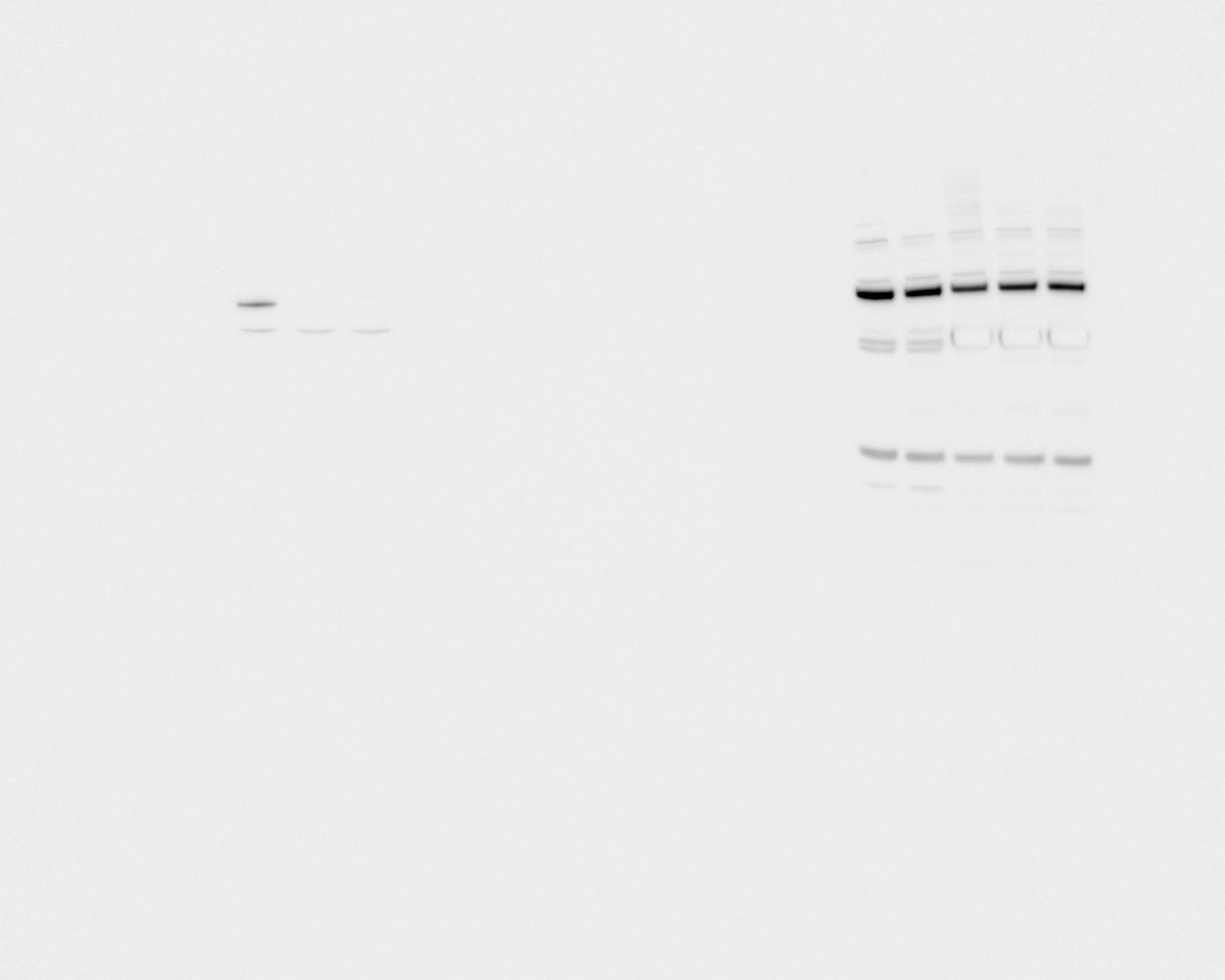

Supplement: Figure 1—source data 2. [file elife-105311-fig1-data2.zip › Figure 1 - Source Data 2/Fig1A_eIF2A_2023-10-17 13h14m13s 13.000s.tif]

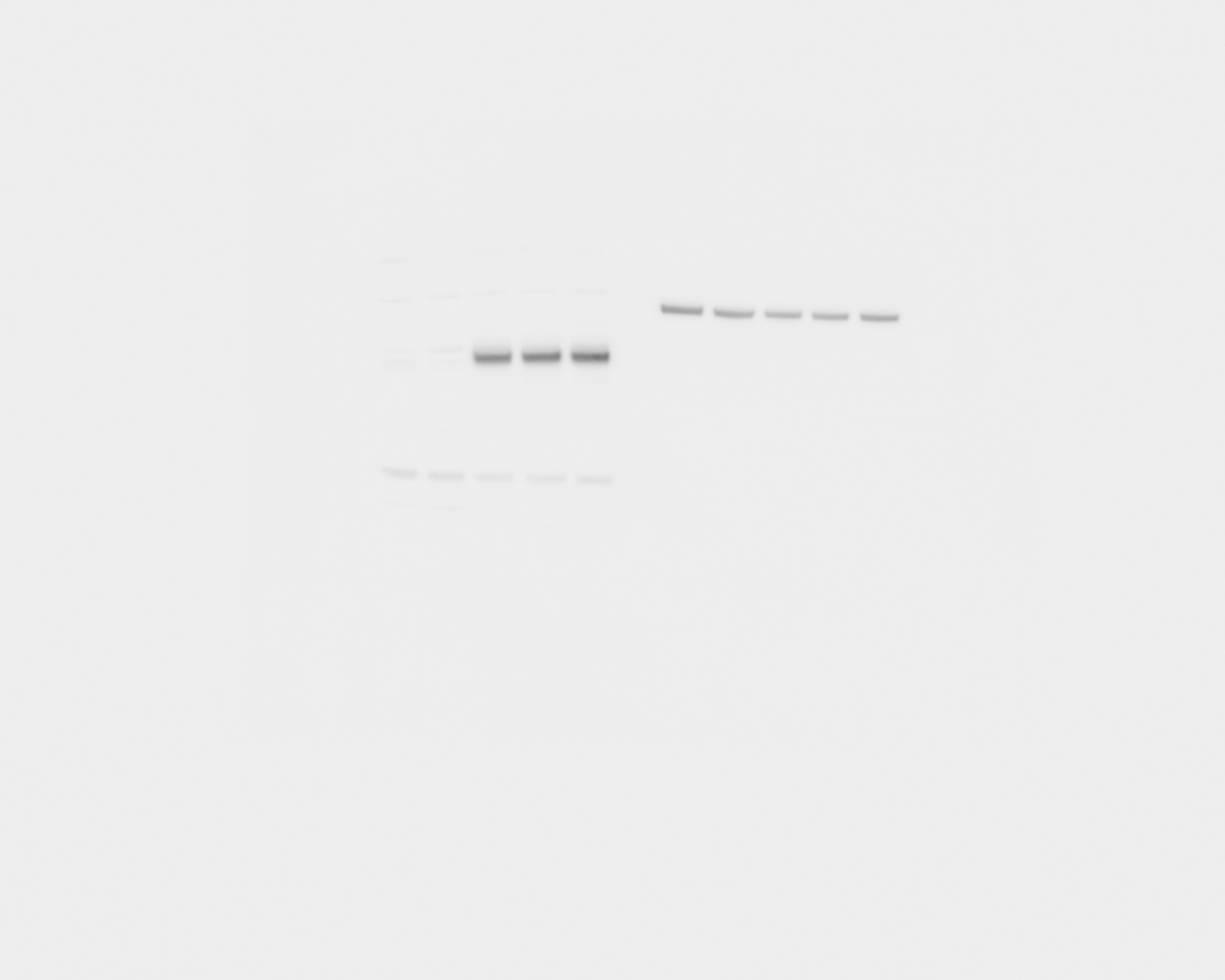

Supplement: Figure 1—source data 2. [file elife-105311-fig1-data2.zip › Figure 1 - Source Data 2/Fig1C_HSP90_2023-10-16 13h01m54s 1.000s.tif]

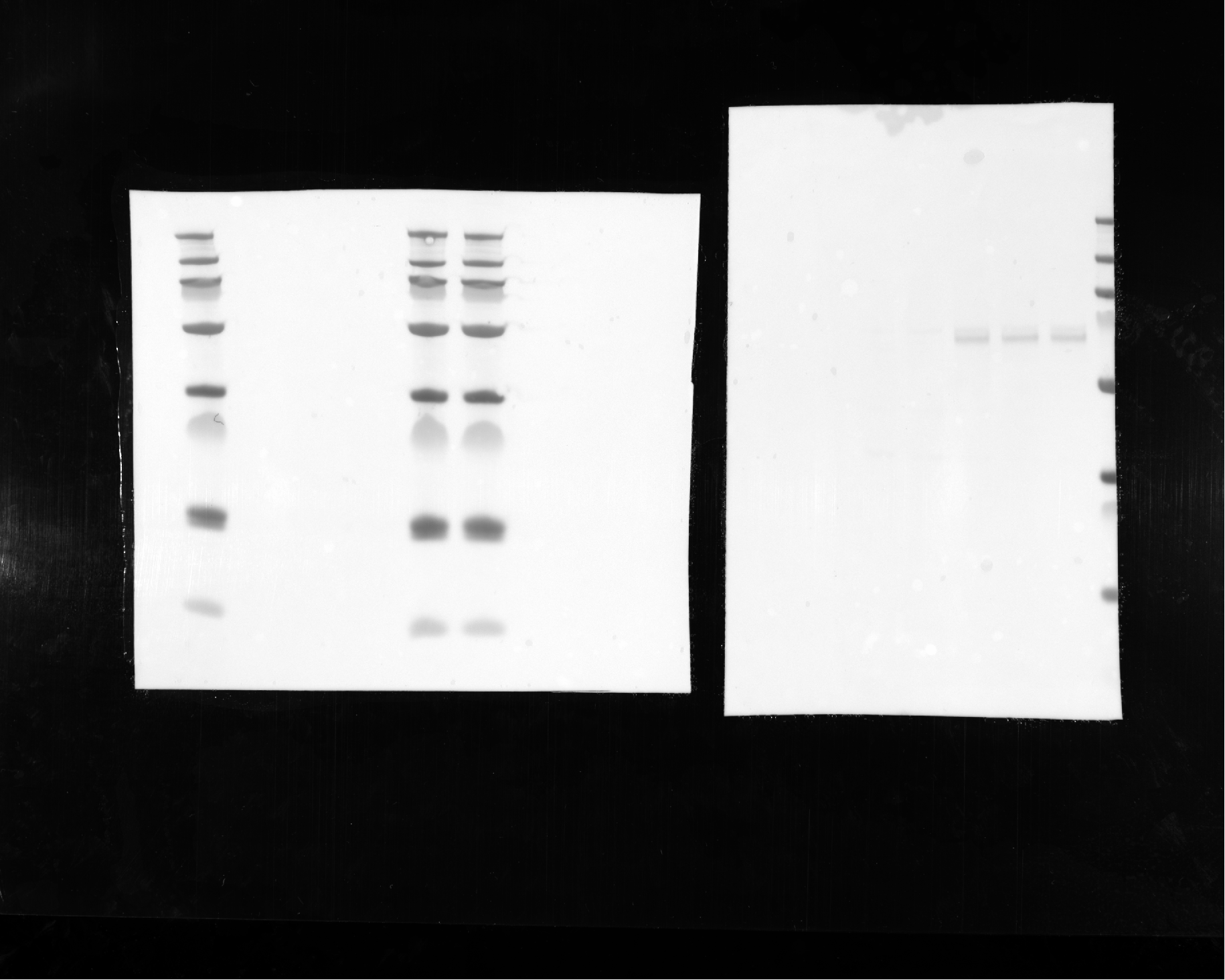

Supplement: Figure 1—source data 2. [file elife-105311-fig1-data2.zip › Figure 1 - Source Data 2/Fig1A_marker_for_eIF2A_2023-10-17 13h15m32s 0.148s.tif]

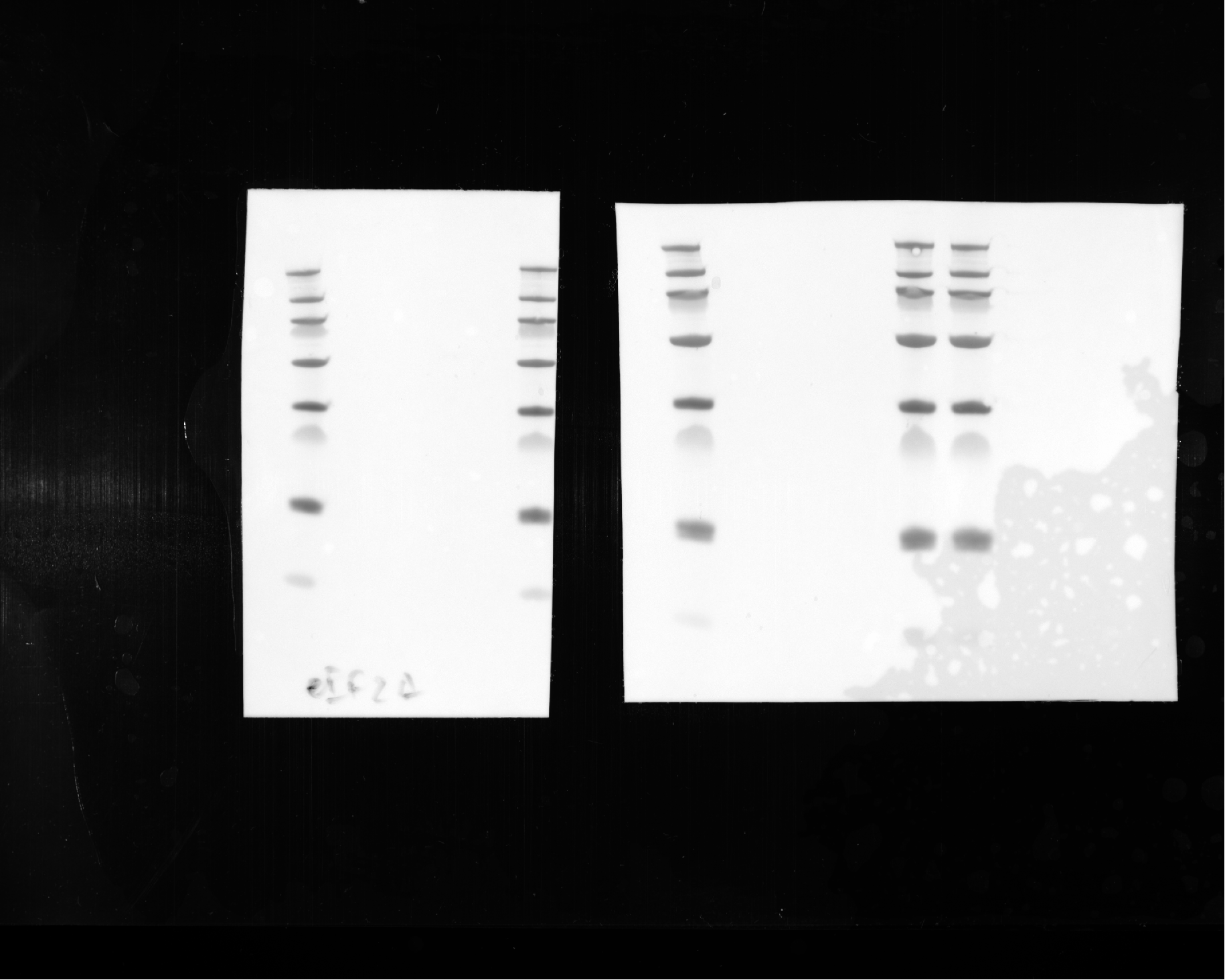

Supplement: Figure 1—source data 2. [file elife-105311-fig1-data2.zip › Figure 1 - Source Data 2/Fig1A_marker_for_HSP90_2023-10-18 13h50m42s 0.156s.tif]

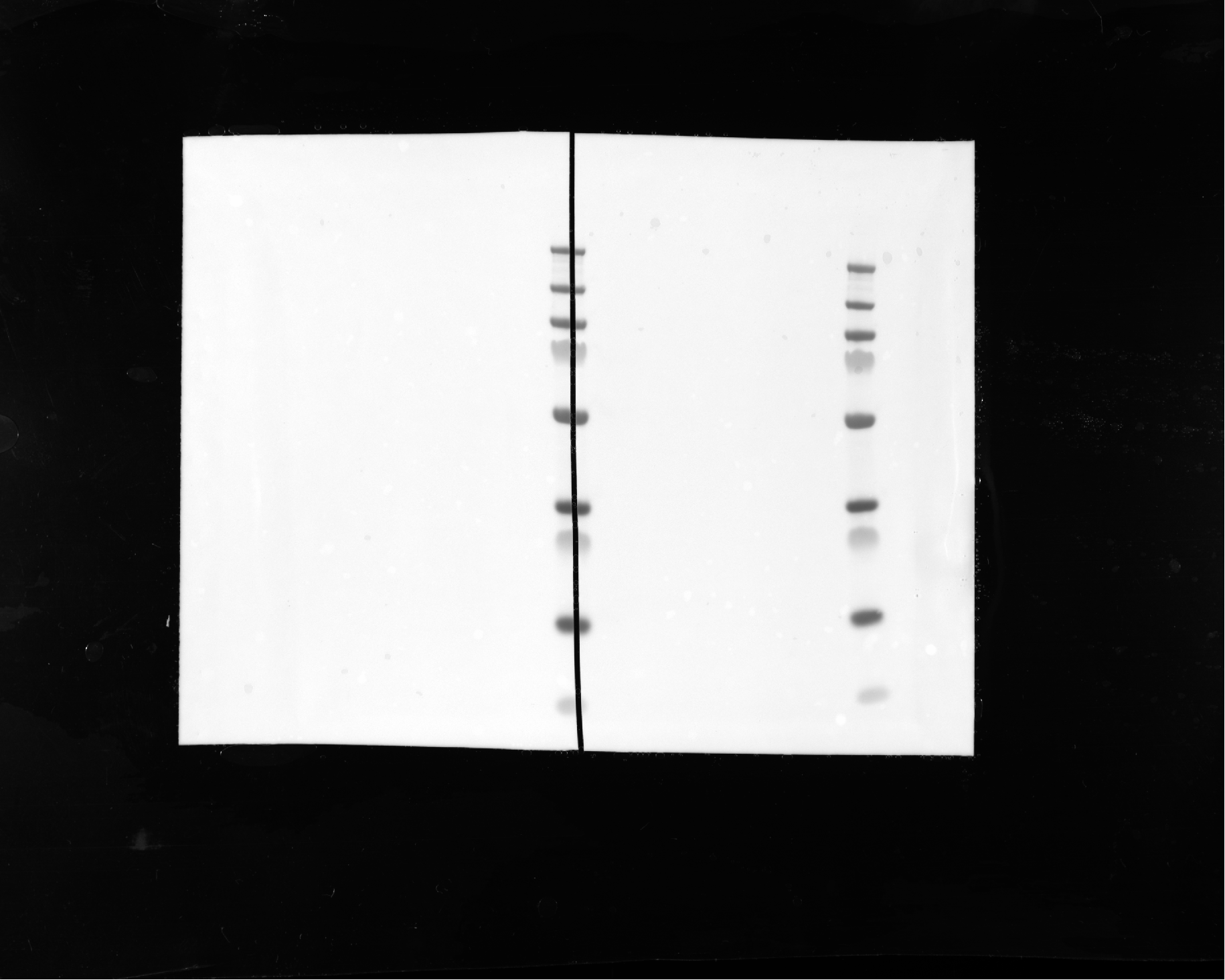

Supplement: Figure 1—source data 2. [file elife-105311-fig1-data2.zip › Figure 1 - Source Data 2/Fig1C_marker_for_puromycin_eIF2A_2023-10-13 14h14m56s 0.160s.tif]

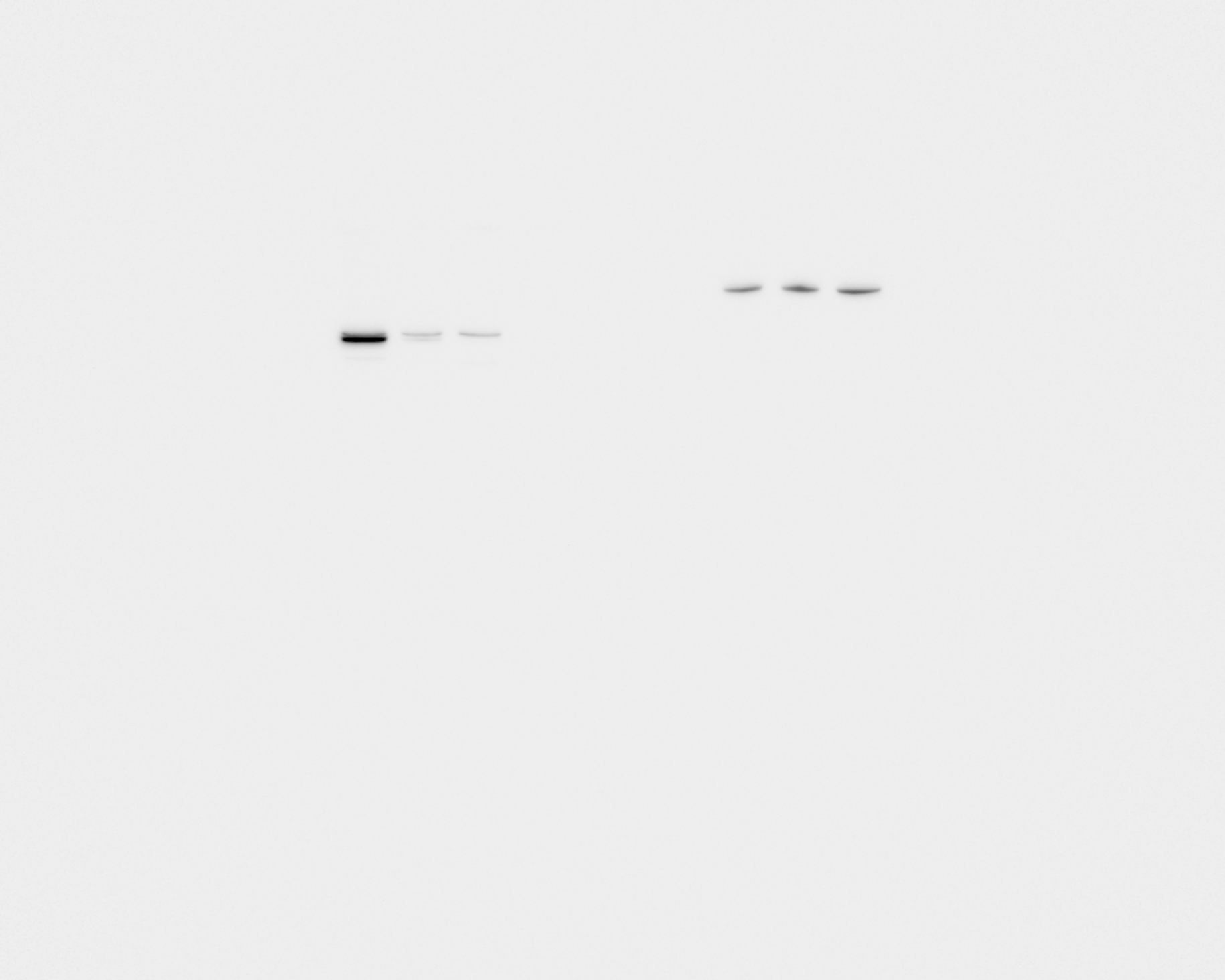

Supplement: Figure 1—source data 2. [file elife-105311-fig1-data2.zip › Figure 1 - Source Data 2/Fig1A_HSP90_2023-10-18 13h47m54s 7.000s.tif]

Fig1 Suppl. Figure 1

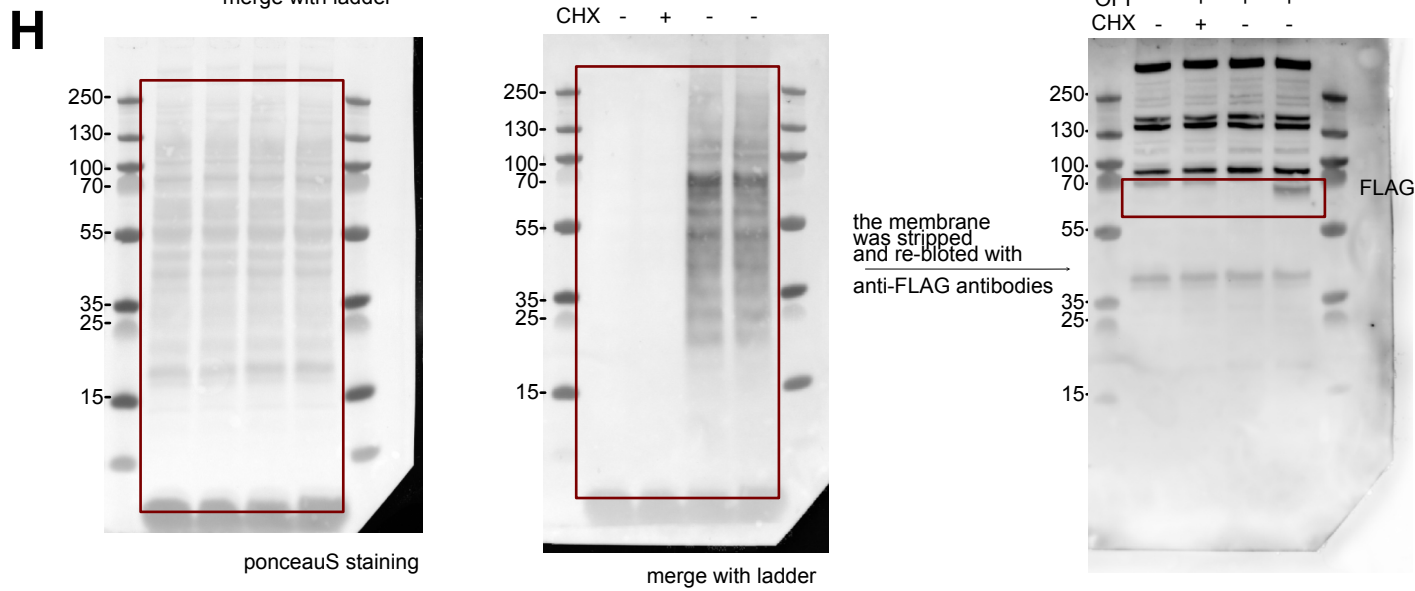

Supplement: Figure 1—figure supplement 1—source data 2. [file elife-105311-fig1-figsupp1-data2.pdf]

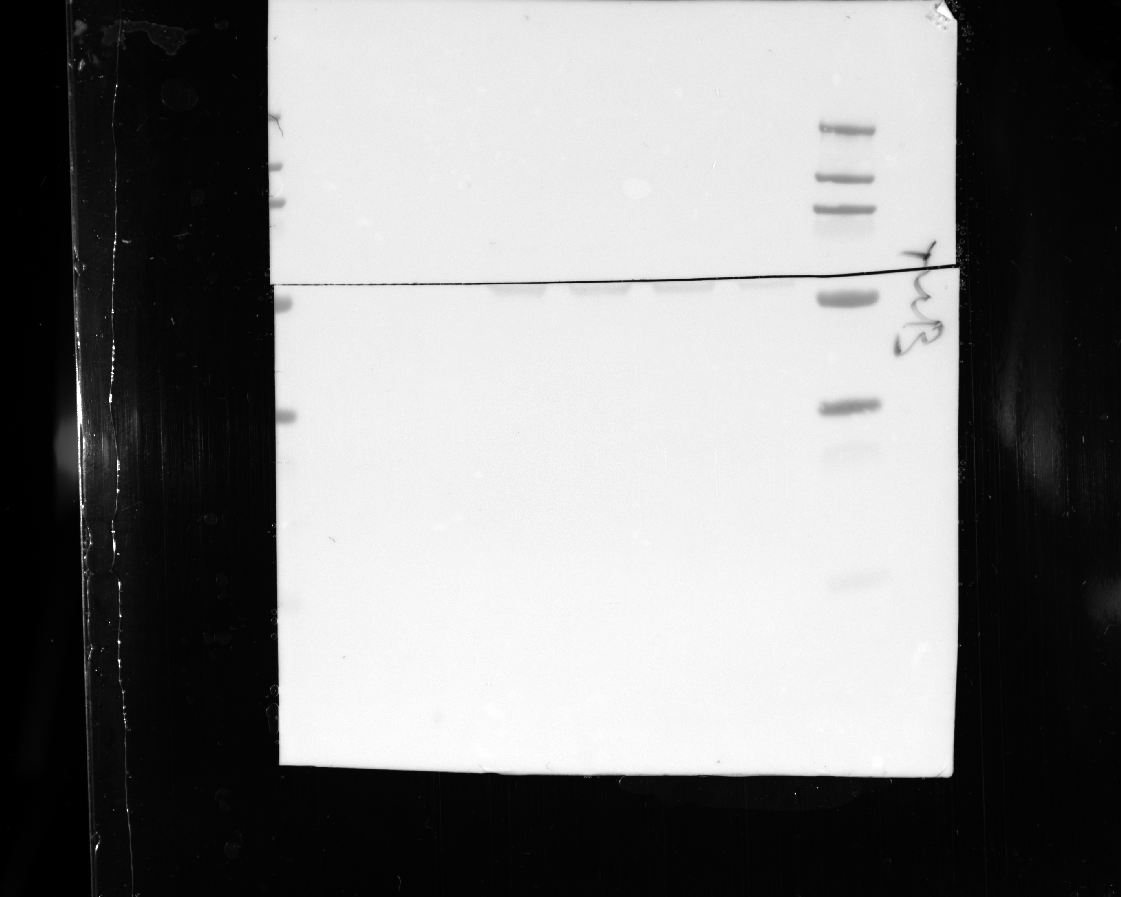

Supplement: Figure 1—figure supplement 1—source data 3. [file elife-105311-fig1-figsupp1-data3.zip › Figure 1 - Figure Supplement 1 - Source Data 3/Fig1_sup1C_marker_for_Lamin_GAPDH_2024-05-10 13h02m18s 0.065s.tif]

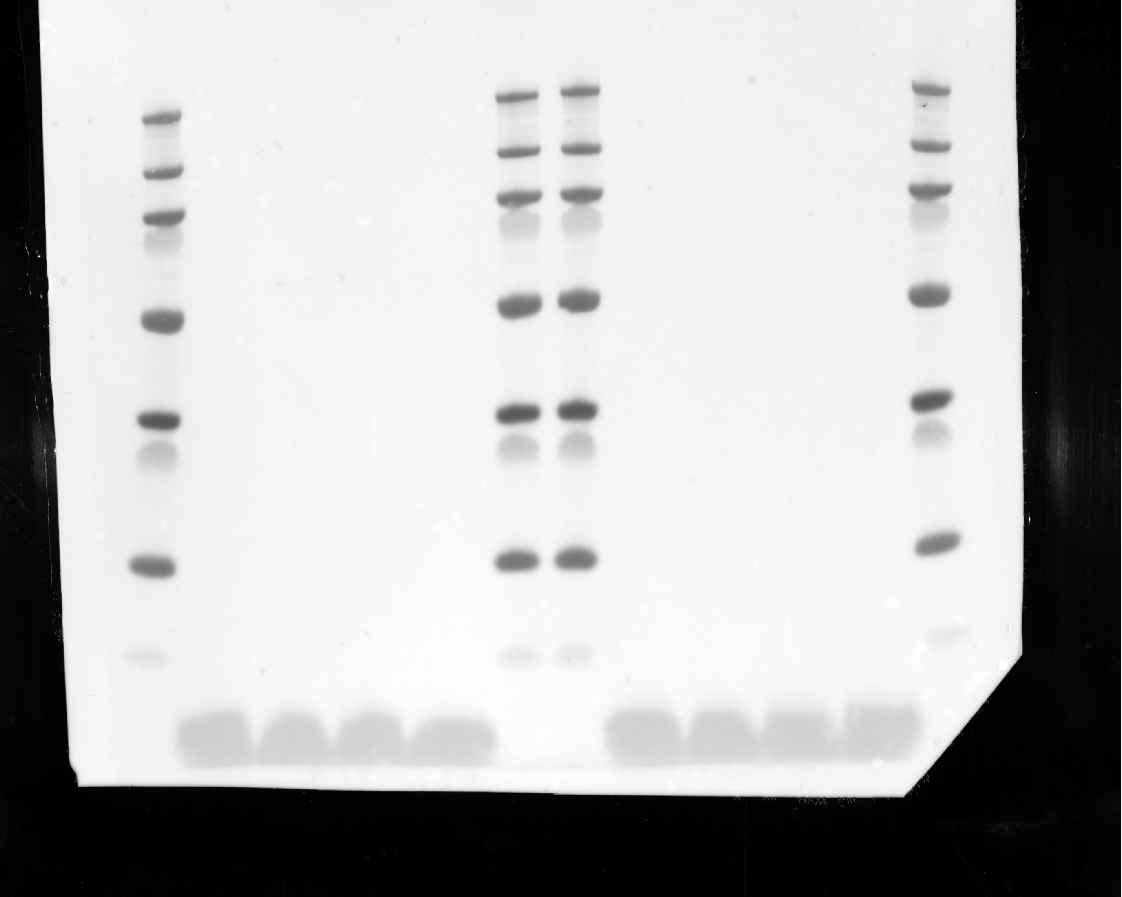

Supplement: Figure 1—figure supplement 1—source data 3. [file elife-105311-fig1-figsupp1-data3.zip › Figure 1 - Figure Supplement 1 - Source Data 3/Fig1_sup1H_marker_for_puromycin_2023-10-20 14h11m22s 0.176s.tif]

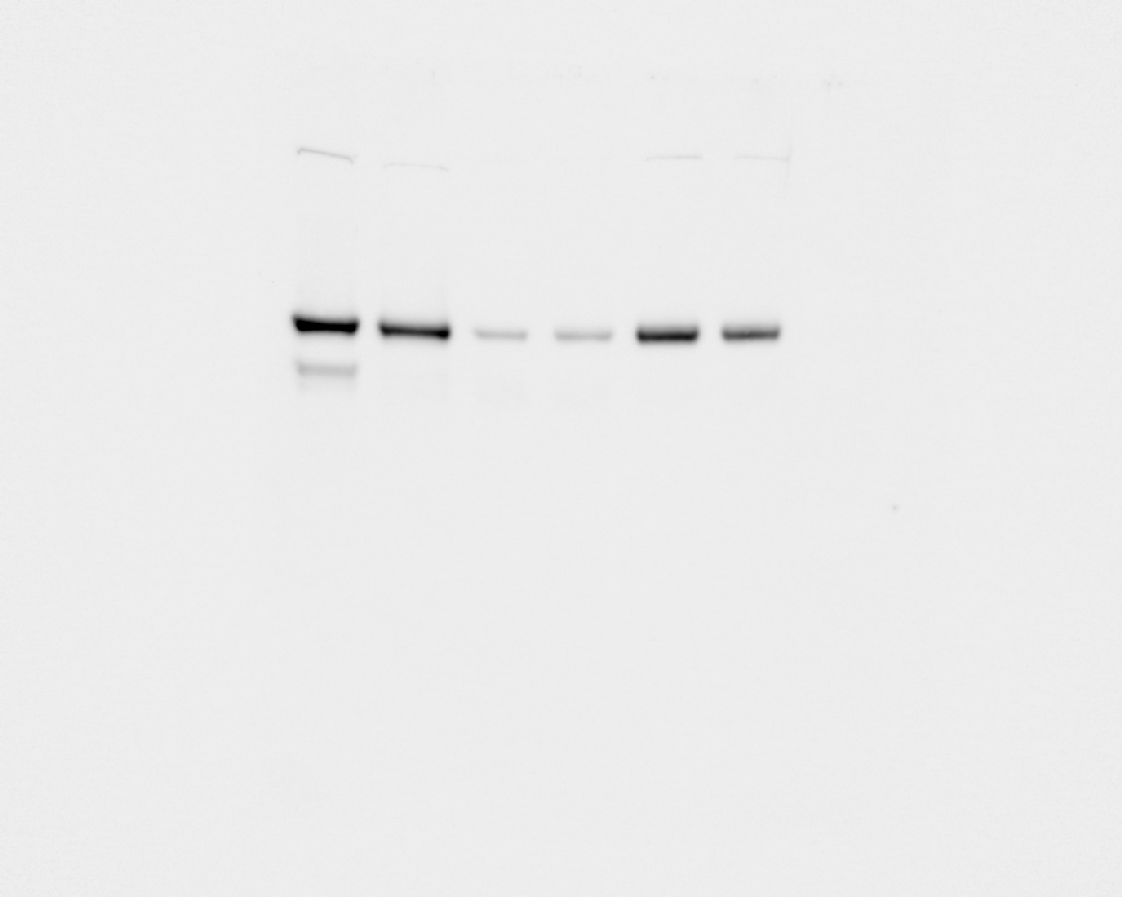

Supplement: Figure 1—figure supplement 1—source data 3. [file elife-105311-fig1-figsupp1-data3.zip › Figure 1 - Figure Supplement 1 - Source Data 3/Fig1_sup1C_ATF4_2024-05-06 13h23m00s 109.375s.tif]

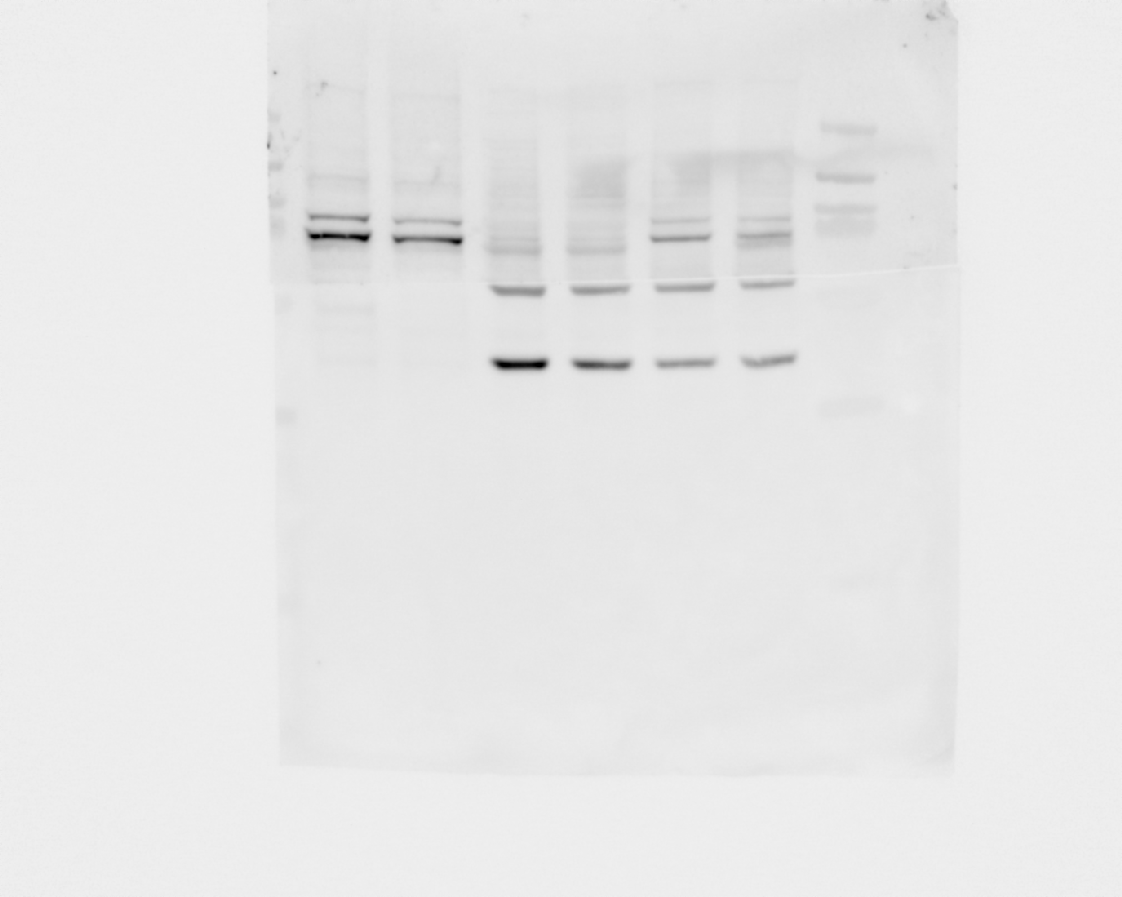

Supplement: Figure 1—figure supplement 1—source data 3. [file elife-105311-fig1-figsupp1-data3.zip › Figure 1 - Figure Supplement 1 - Source Data 3/Fig1_sup1C_Lamin_GAPDH_2024-05-10 13h01m39s 8.000s.tif]

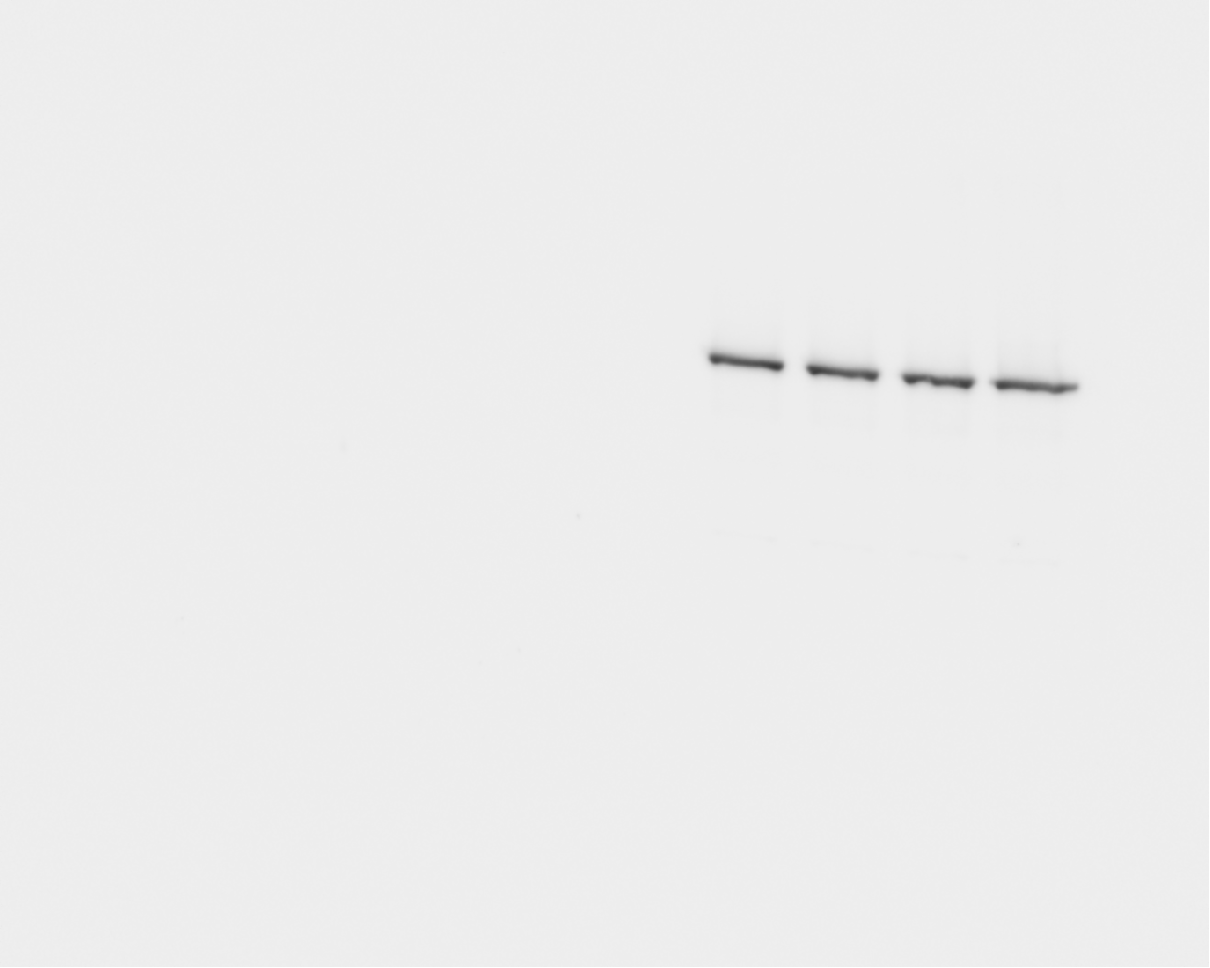

Supplement: Figure 1—figure supplement 1—source data 3. [file elife-105311-fig1-figsupp1-data3.zip › Figure 1 - Figure Supplement 1 - Source Data 3/Fig1_sup1F_HSP90_2024-07-31 15h00m22s 1.000s.tif]

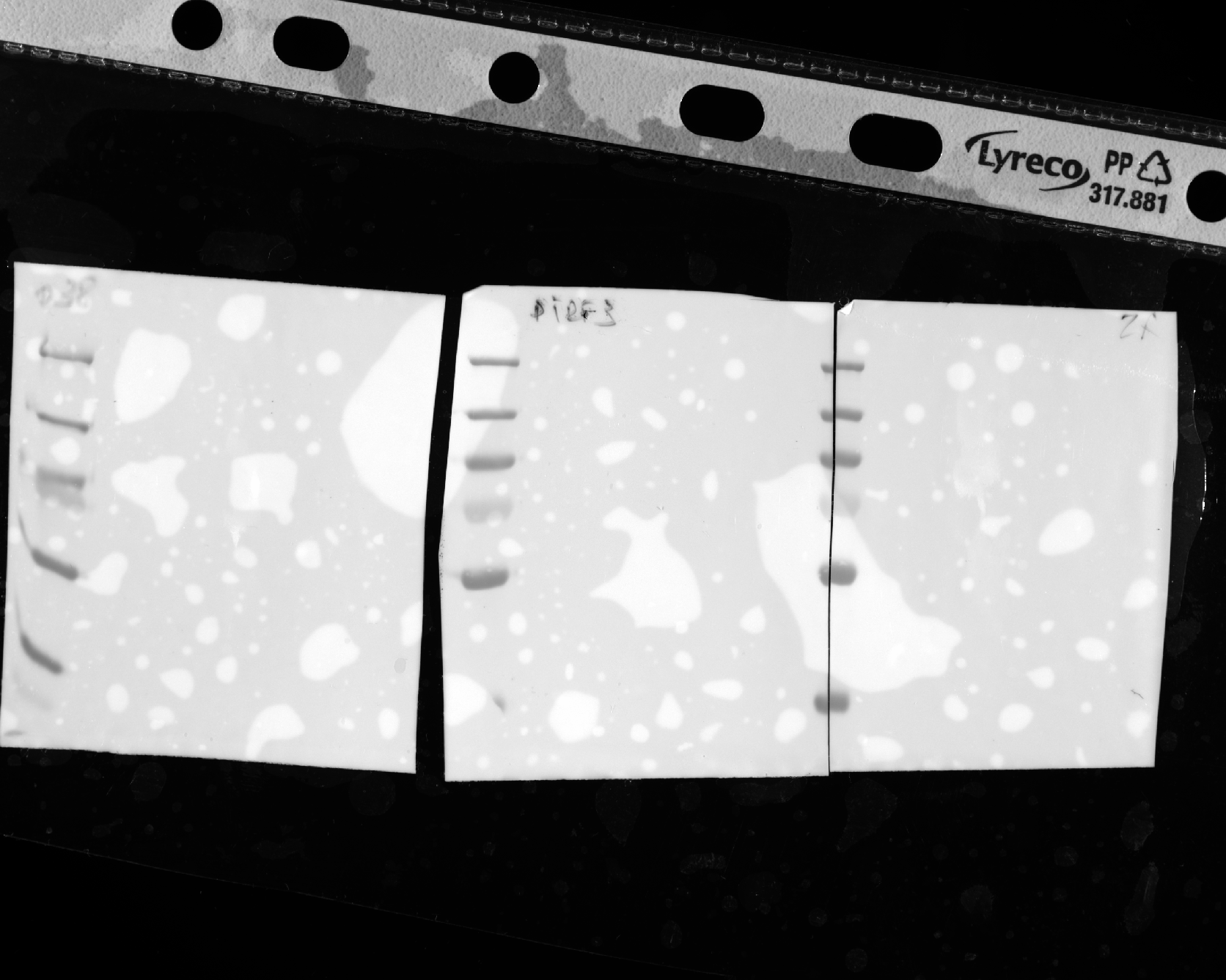

Supplement: Figure 1—figure supplement 1—source data 3. [file elife-105311-fig1-figsupp1-data3.zip › Figure 1 - Figure Supplement 1 - Source Data 3/Fig1_sup1F_marker_for_eIF2A_2024-07-30 15h12m20s 0.159s.tif]

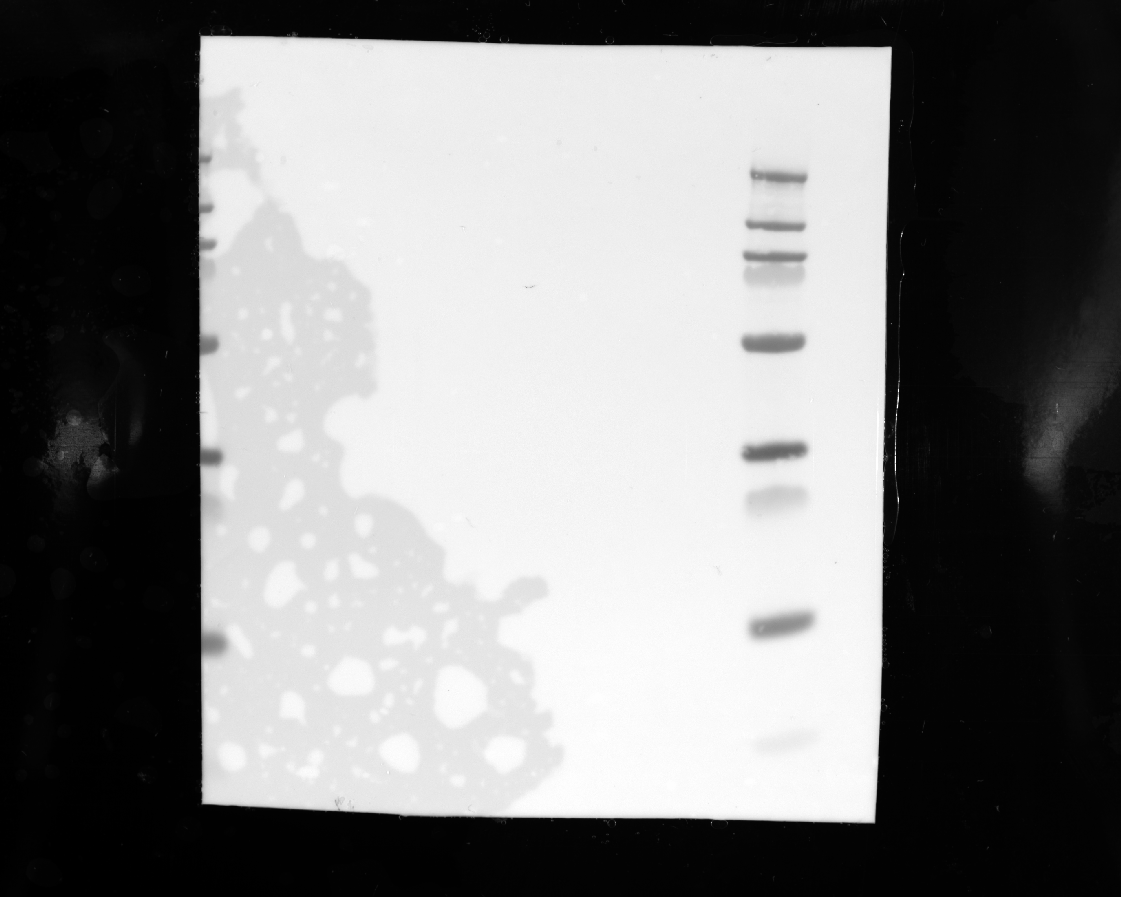

Supplement: Figure 1—figure supplement 1—source data 3. [file elife-105311-fig1-figsupp1-data3.zip › Figure 1 - Figure Supplement 1 - Source Data 3/Fig1_sup1C_marker_for_eIF2A_2024-05-03 13h31m52s 0.180s.tif]

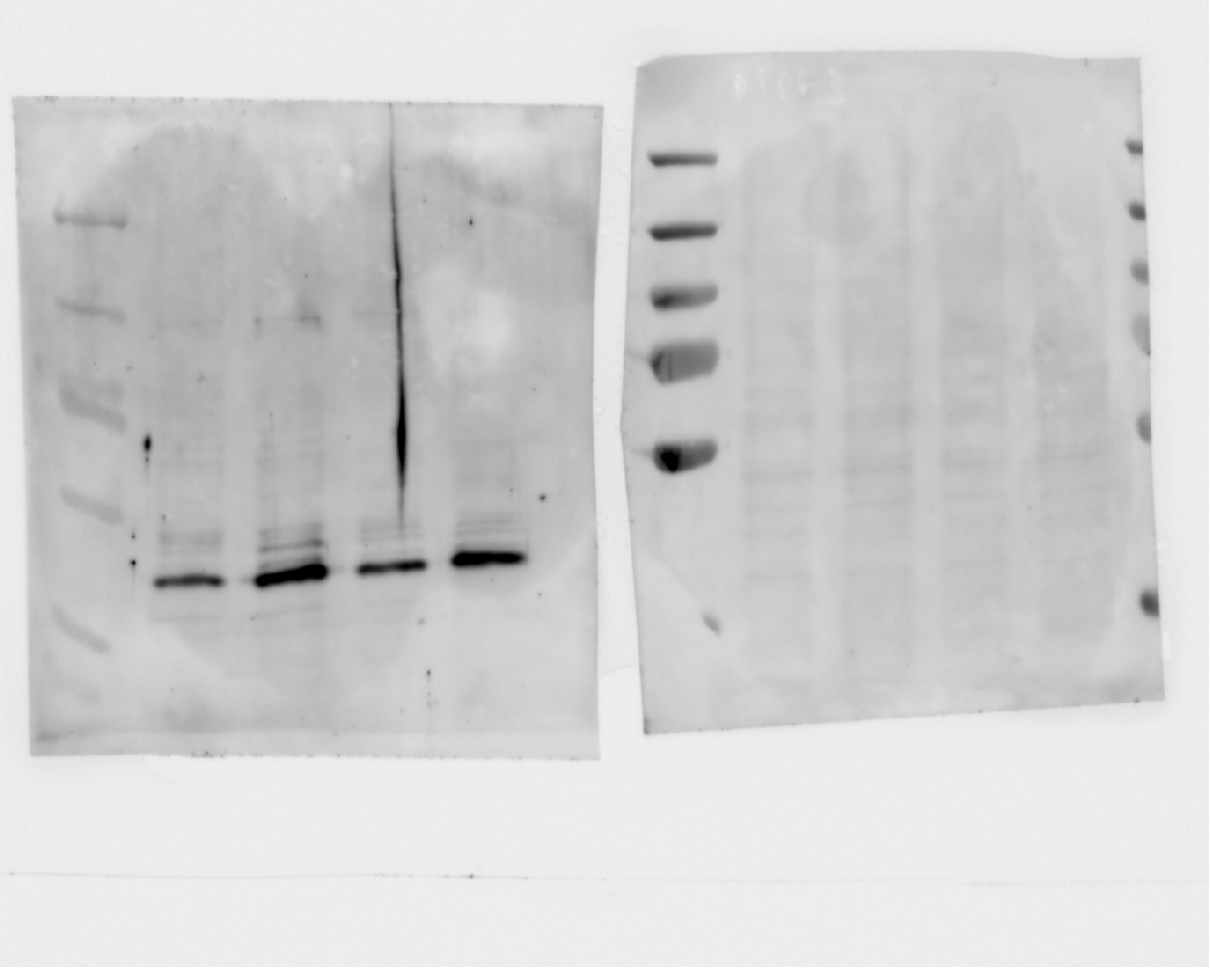

Supplement: Figure 1—figure supplement 1—source data 3. [file elife-105311-fig1-figsupp1-data3.zip › Figure 1 - Figure Supplement 1 - Source Data 3/Fig1_sup1F_p-p38_2024-07-30 15h15m25s 13.000s.tif]

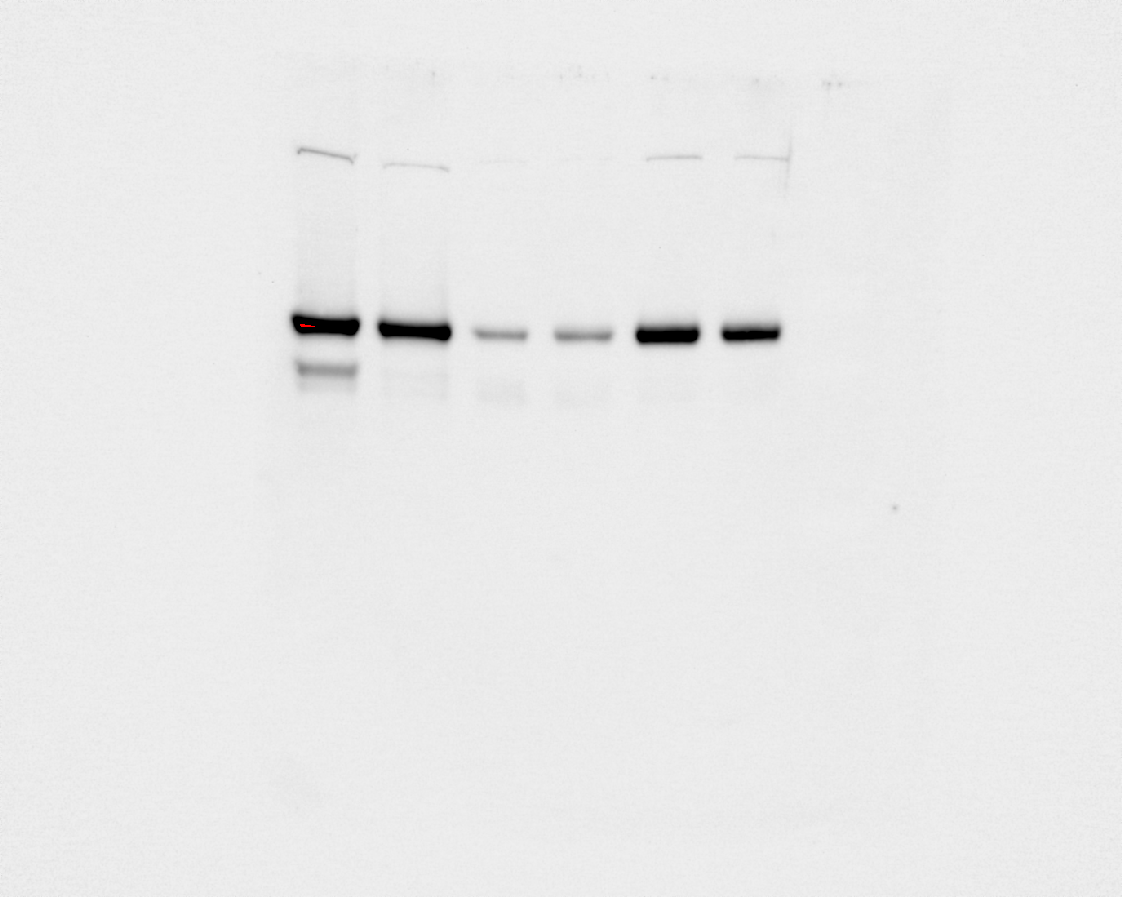

Supplement: Figure 1—figure supplement 1—source data 3. [file elife-105311-fig1-figsupp1-data3.zip › Figure 1 - Figure Supplement 1 - Source Data 3/Fig1_sup1C_cMYC_2024-05-06 13h25m30s 250.000s.tif]

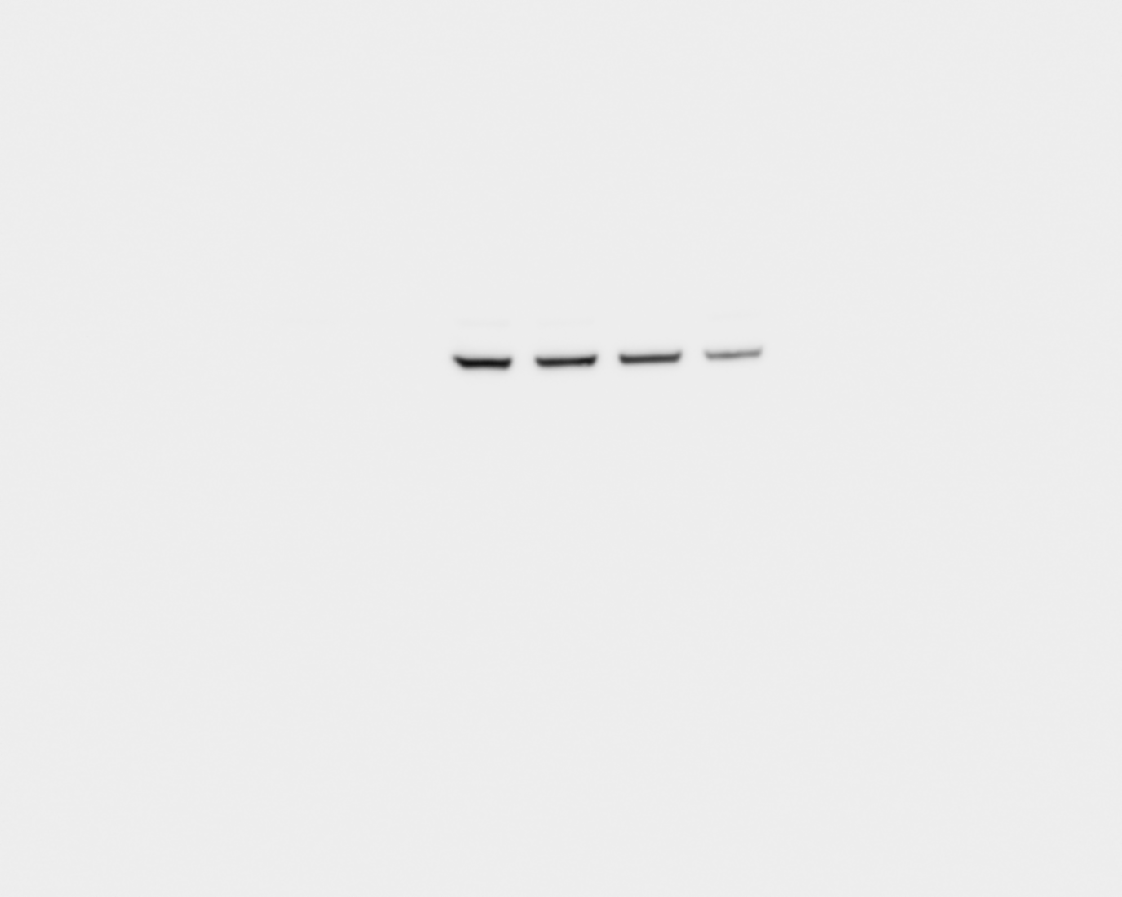

Supplement: Figure 1—figure supplement 1—source data 3. [file elife-105311-fig1-figsupp1-data3.zip › Figure 1 - Figure Supplement 1 - Source Data 3/Fig1_sup1C_tubulin_2024-05-07 14h48m52s 1.000s.tif]

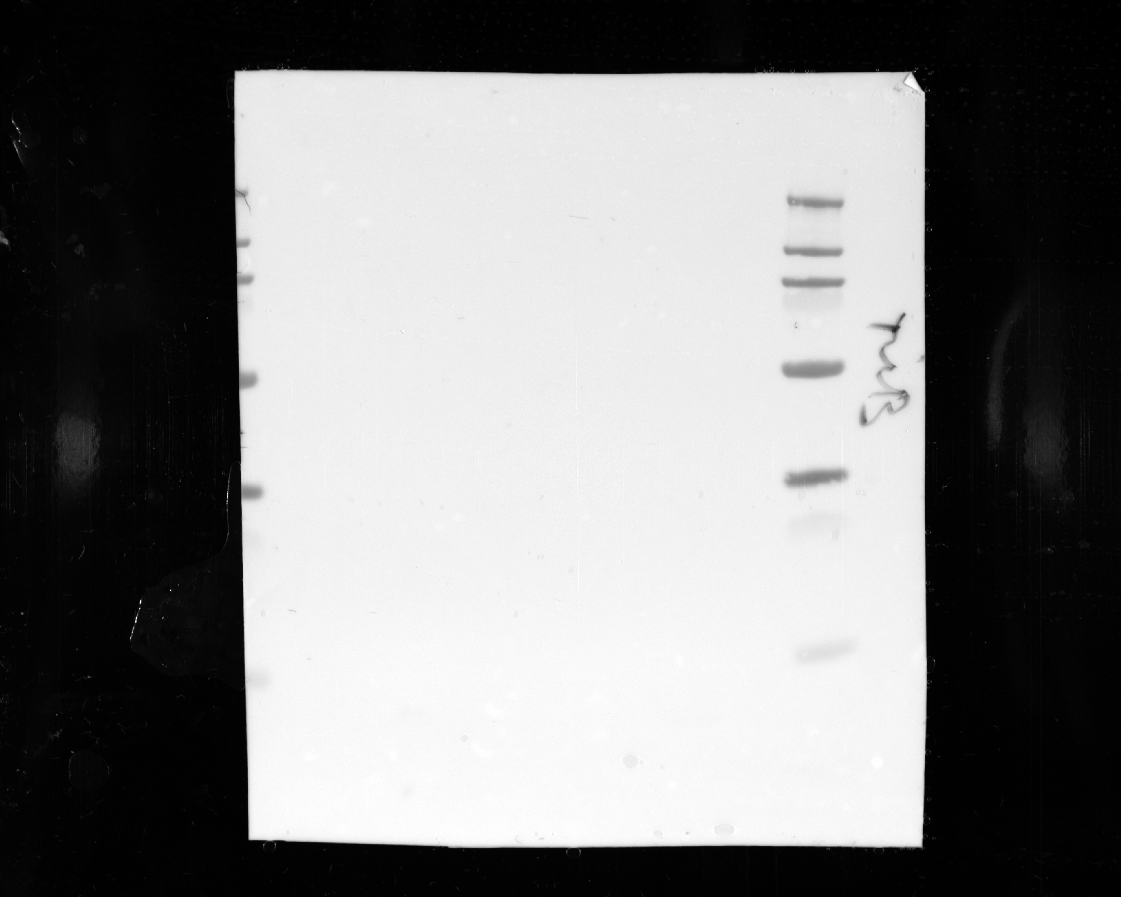

Supplement: Figure 1—figure supplement 1—source data 3. [file elife-105311-fig1-figsupp1-data3.zip › Figure 1 - Figure Supplement 1 - Source Data 3/Fig1_sup1C_marker_for_tubulin_2024-05-07 14h50m53s 0.143s.tif]

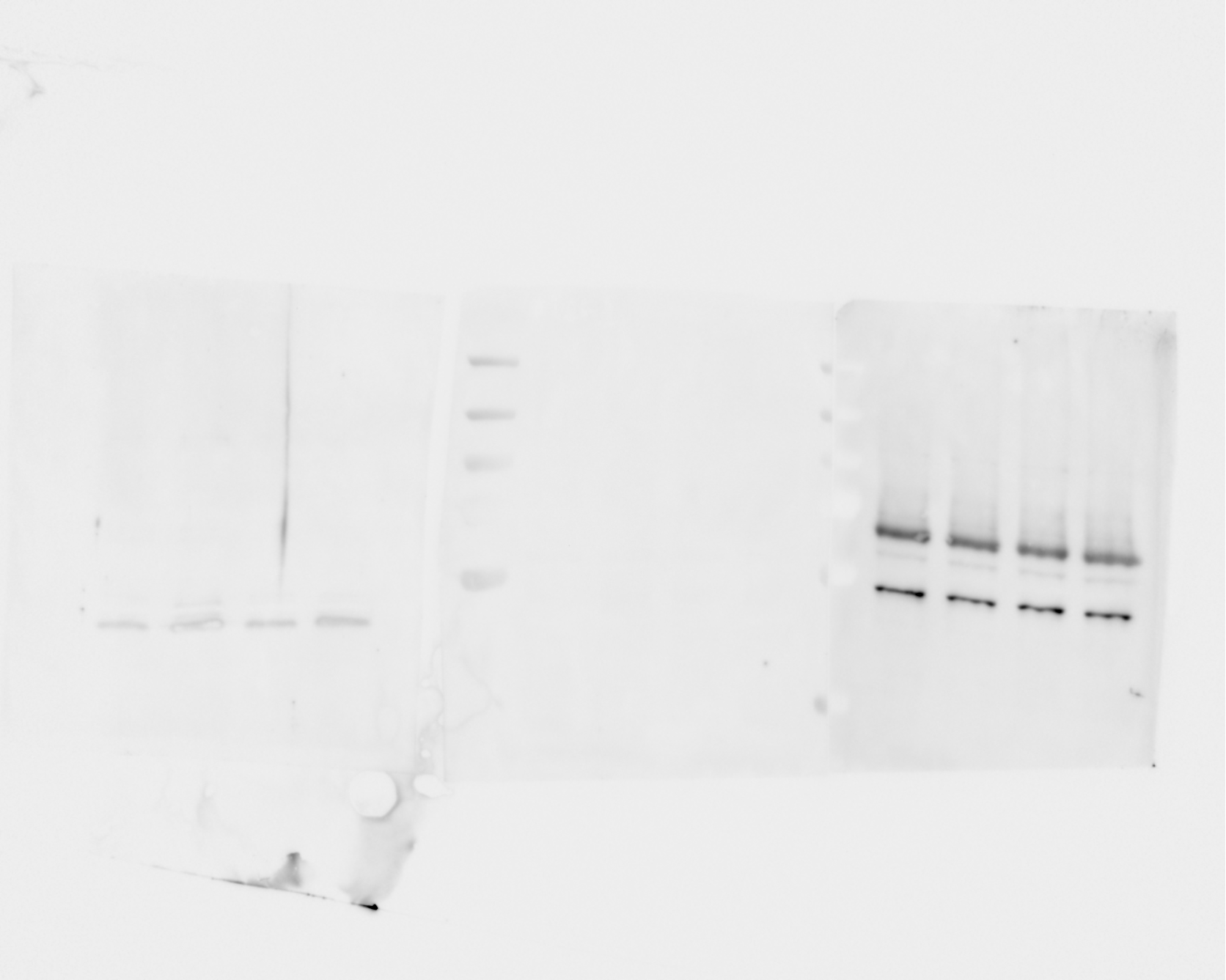

Supplement: Figure 1—figure supplement 1—source data 3. [file elife-105311-fig1-figsupp1-data3.zip › Figure 1 - Figure Supplement 1 - Source Data 3/Fig1_sup1F_eIF2A_2024-07-30 15h09m48s 37.500s.tif]

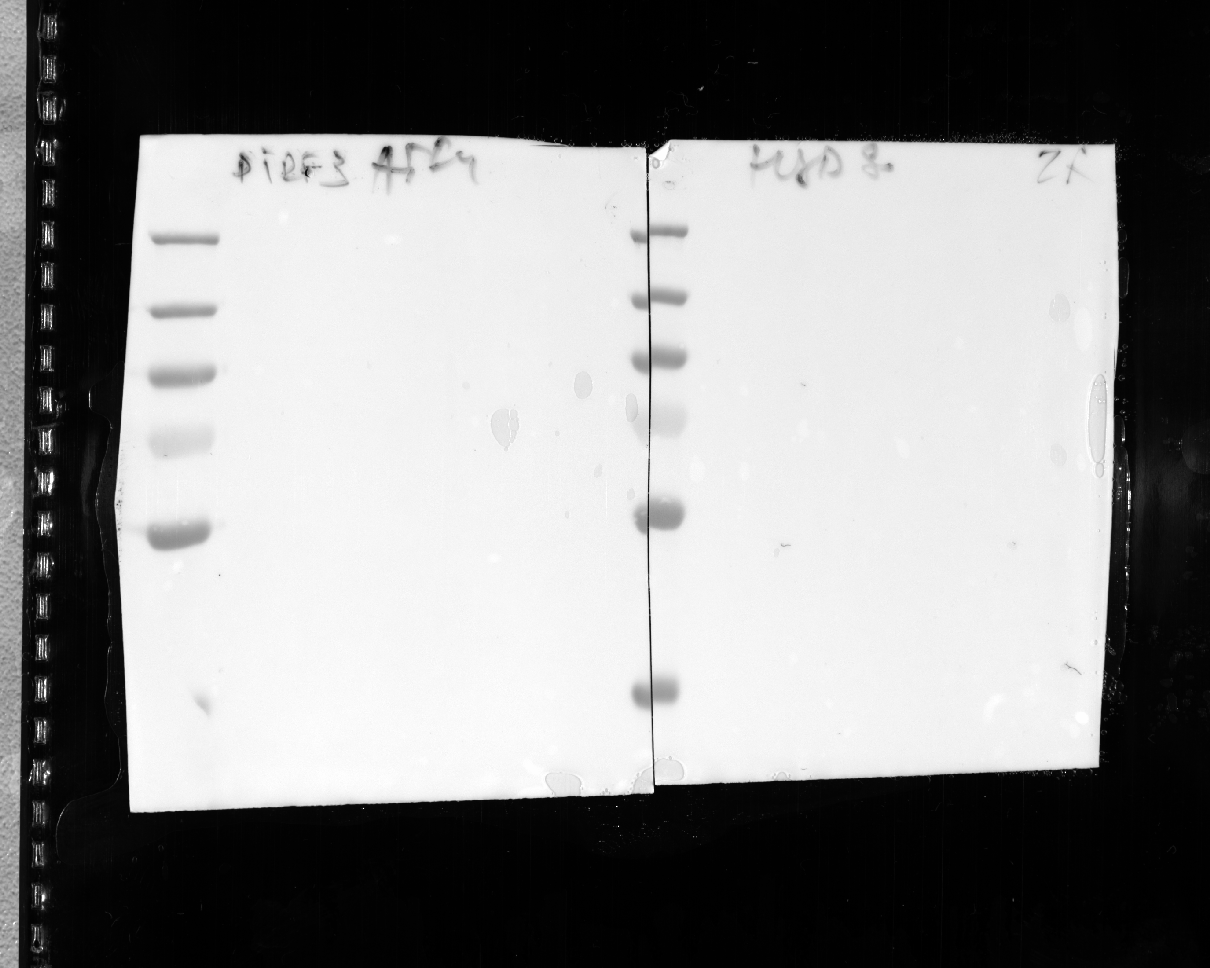

Supplement: Figure 1—figure supplement 1—source data 3. [file elife-105311-fig1-figsupp1-data3.zip › Figure 1 - Figure Supplement 1 - Source Data 3/Fig1_sup1F_marker_for_HSP90_2024-07-31 15h02m55s 0.100s.tif]

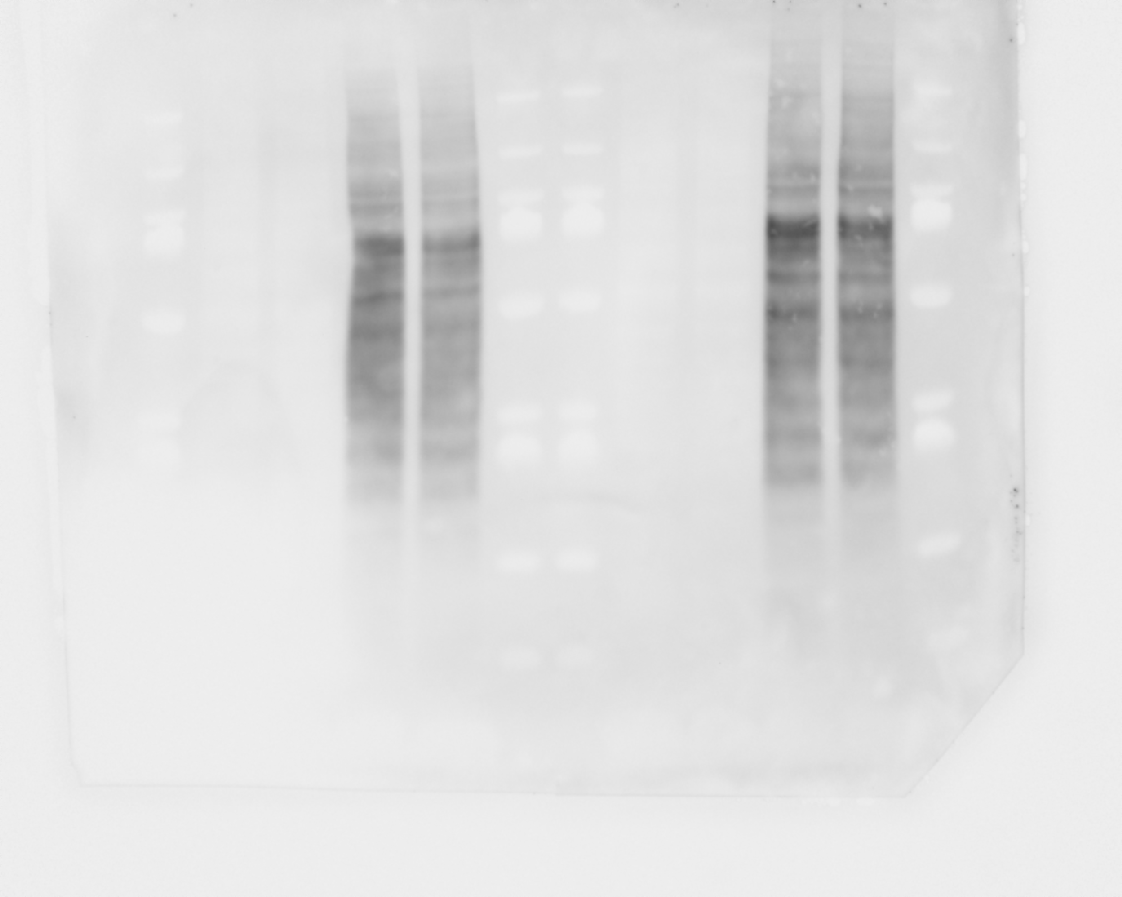

Supplement: Figure 1—figure supplement 1—source data 3. [file elife-105311-fig1-figsupp1-data3.zip › Figure 1 - Figure Supplement 1 - Source Data 3/Fig1_sup1H_puromycin_2023-10-20 14h08m23s 7.000s.tif]

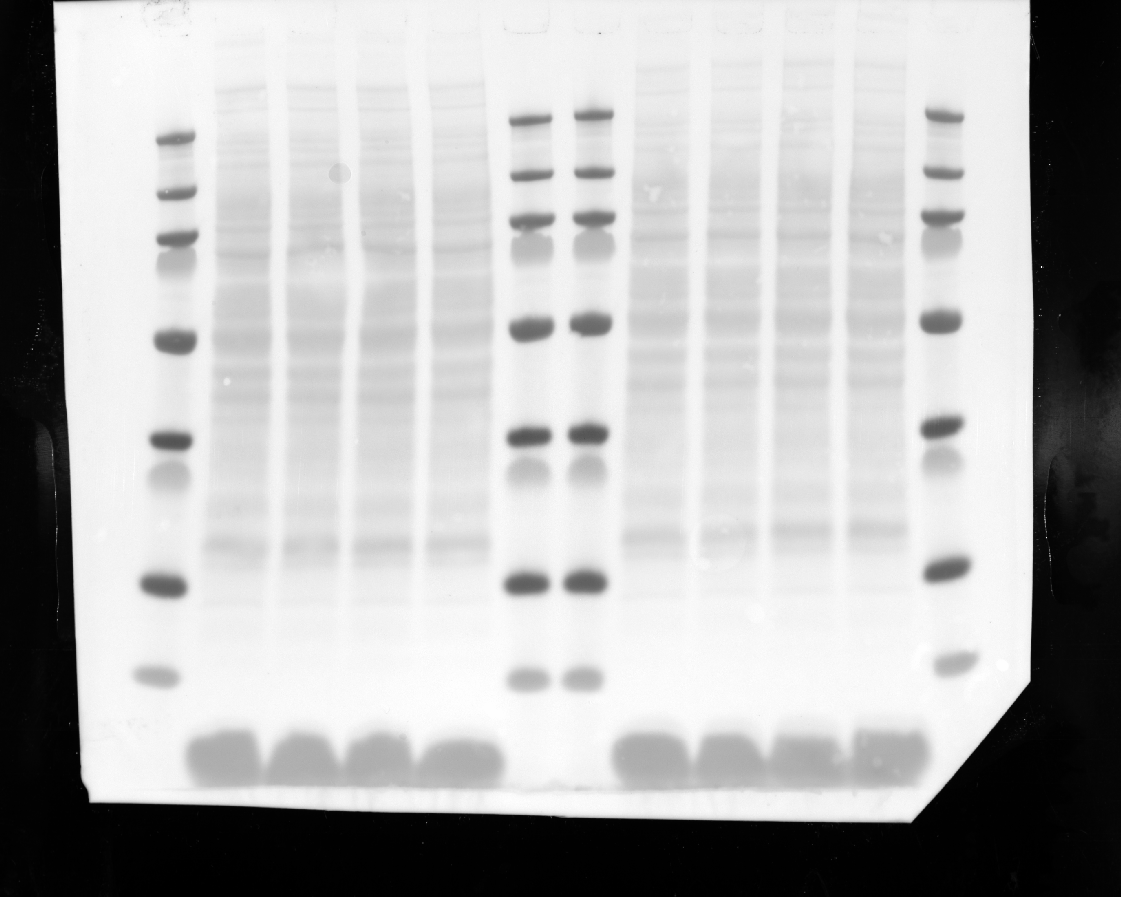

Supplement: Figure 1—figure supplement 1—source data 3. [file elife-105311-fig1-figsupp1-data3.zip › Figure 1 - Figure Supplement 1 - Source Data 3/Fig1_sup1H_ponceauS_2023-10-19 18h13m02s 0.175s.tif]

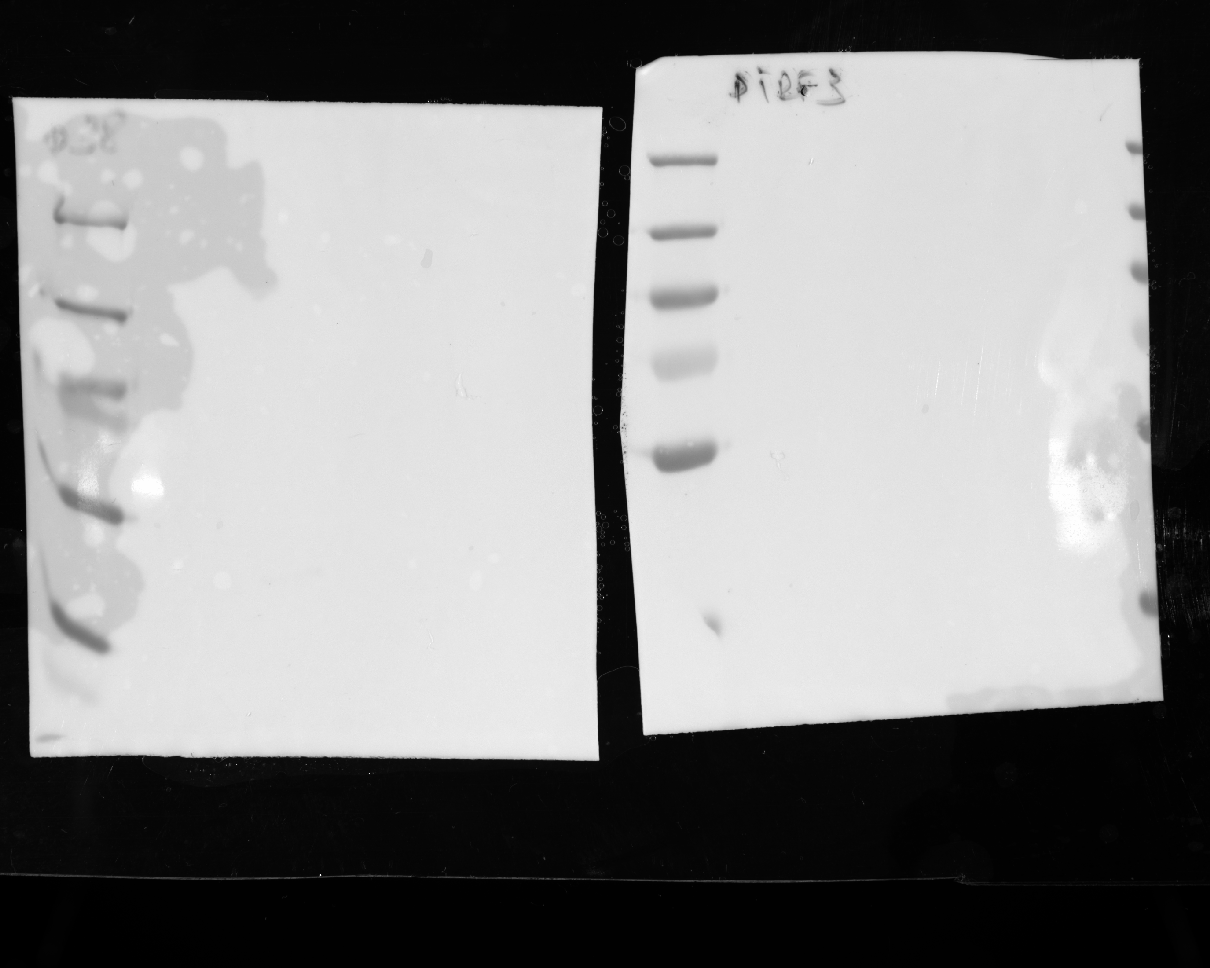

Supplement: Figure 1—figure supplement 1—source data 3. [file elife-105311-fig1-figsupp1-data3.zip › Figure 1 - Figure Supplement 1 - Source Data 3/Fig1_sup1F_marker_for_pp38_2024-07-30 15h18m54s 0.156s.tif]

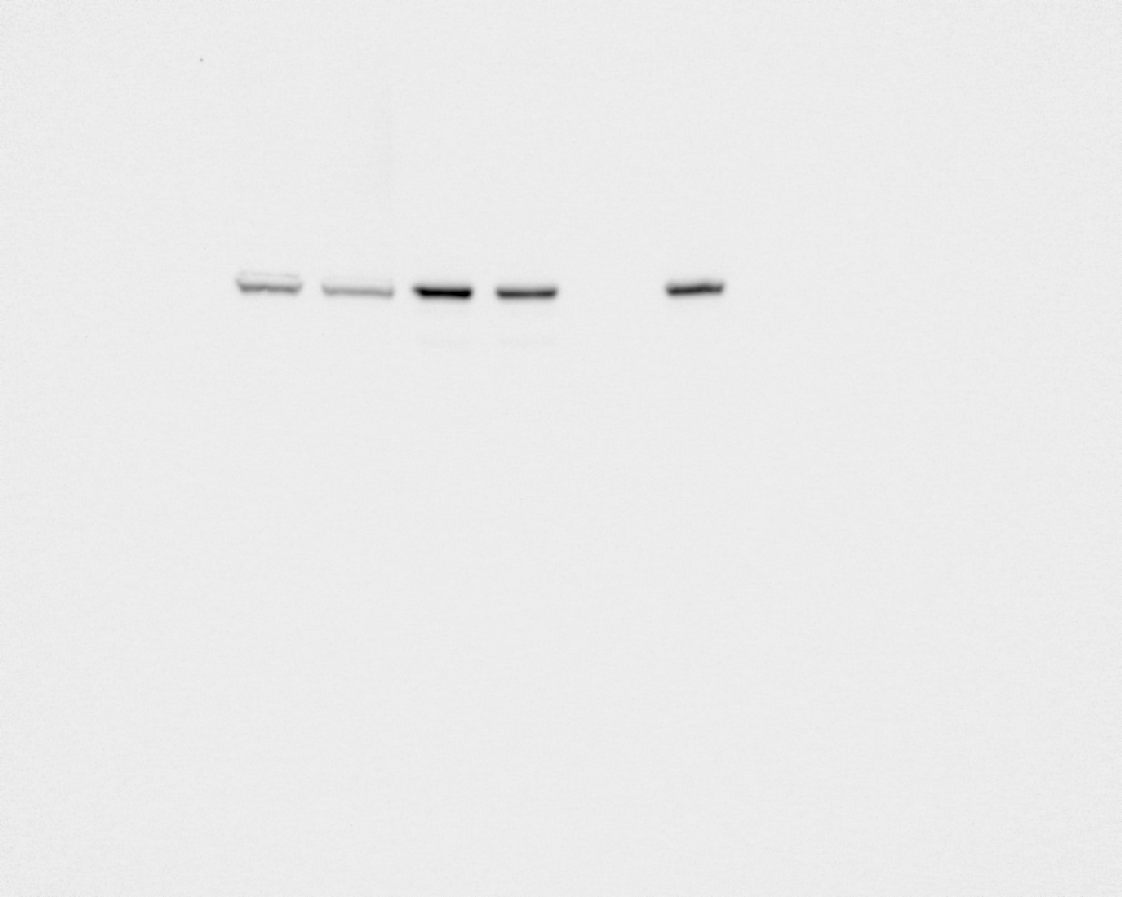

Supplement: Figure 1—figure supplement 1—source data 3. [file elife-105311-fig1-figsupp1-data3.zip › Figure 1 - Figure Supplement 1 - Source Data 3/Fig1_sup1C_eIF2A_2024-05-03 13h24m21s 22.000s.tif]

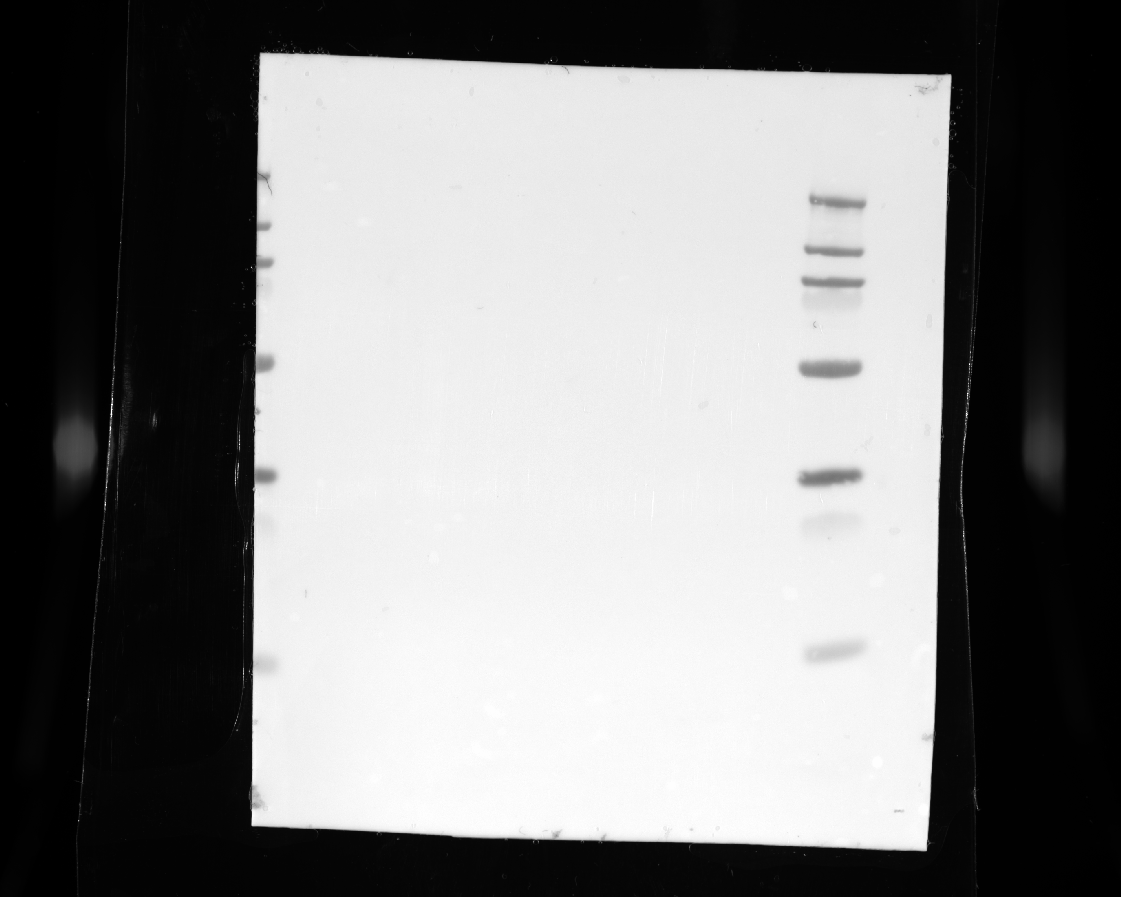

Supplement: Figure 1—figure supplement 1—source data 3. [file elife-105311-fig1-figsupp1-data3.zip › Figure 1 - Figure Supplement 1 - Source Data 3/Fig1_sup1C_marker_for_ATF4_CMYC_2024-05-06 13h28m26s 0.186s.tif]

# Figure 2

same samples were run in a parallel and blotted for the different proteins:

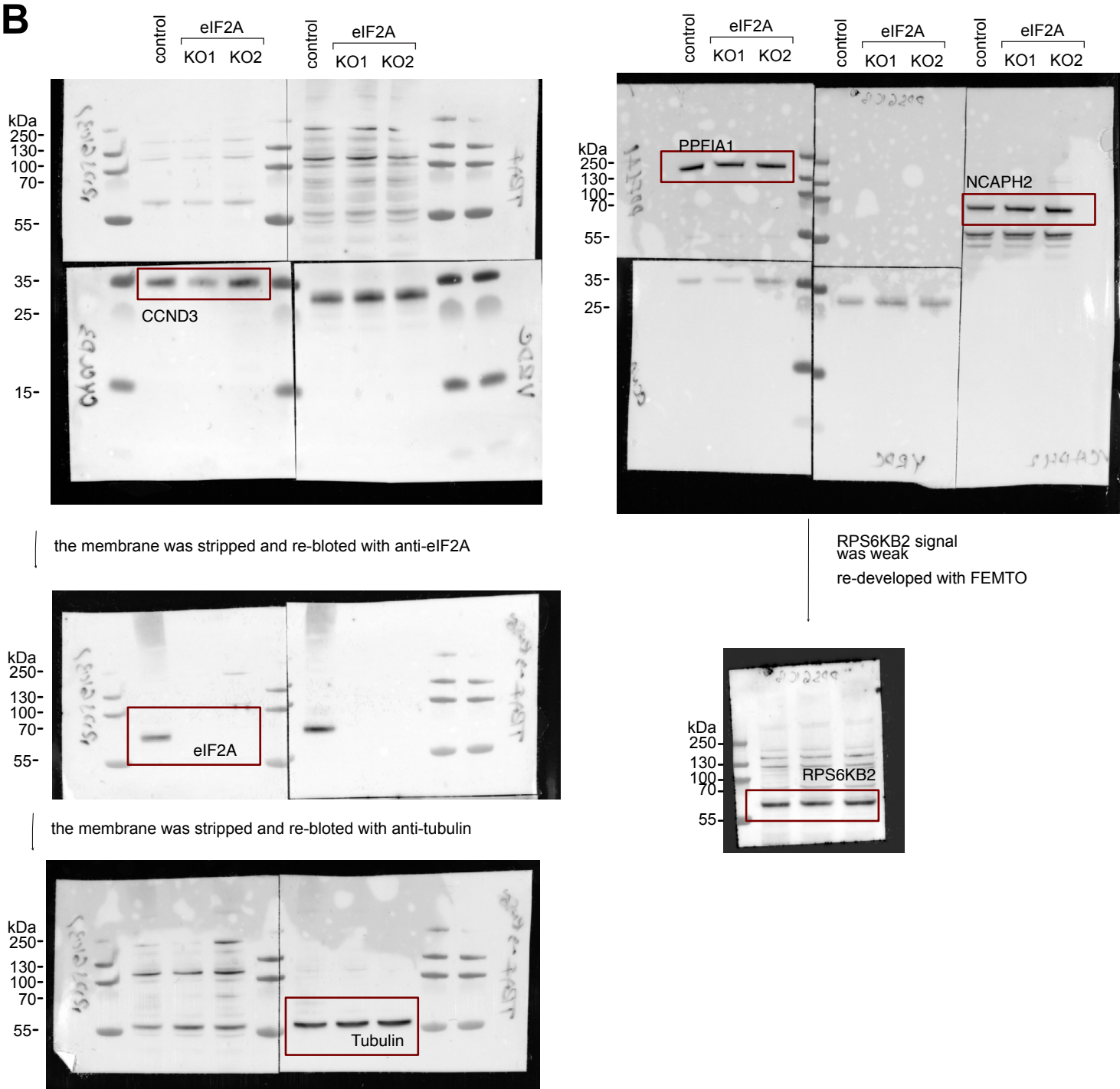

Supplement: Figure 2—source data 1. [file elife-105311-fig2-data1.pdf]

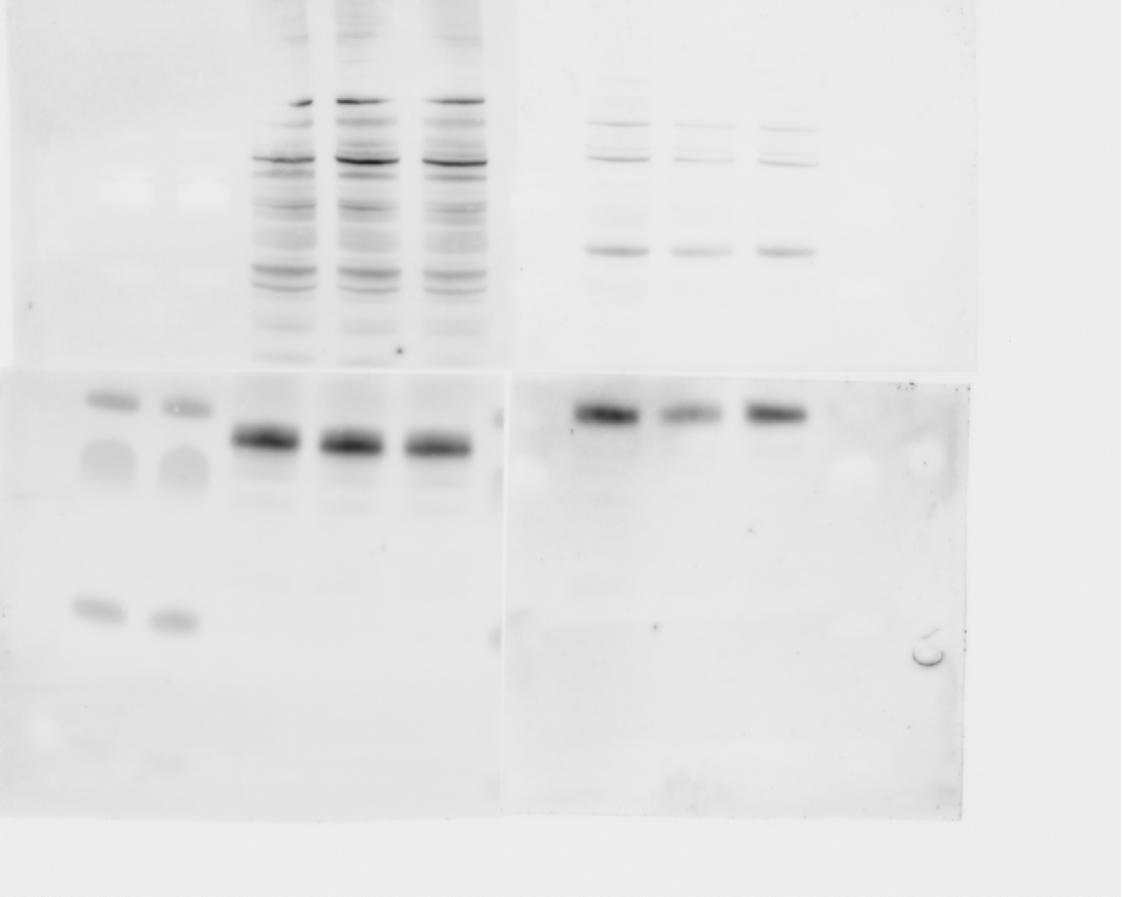

Supplement: Figure 2—source data 2. [file elife-105311-fig2-data2.zip › Figure 2 - Source Data 2/Fig2C_CCND3_2024-04-16 14h23m25s 19.000s.tif]

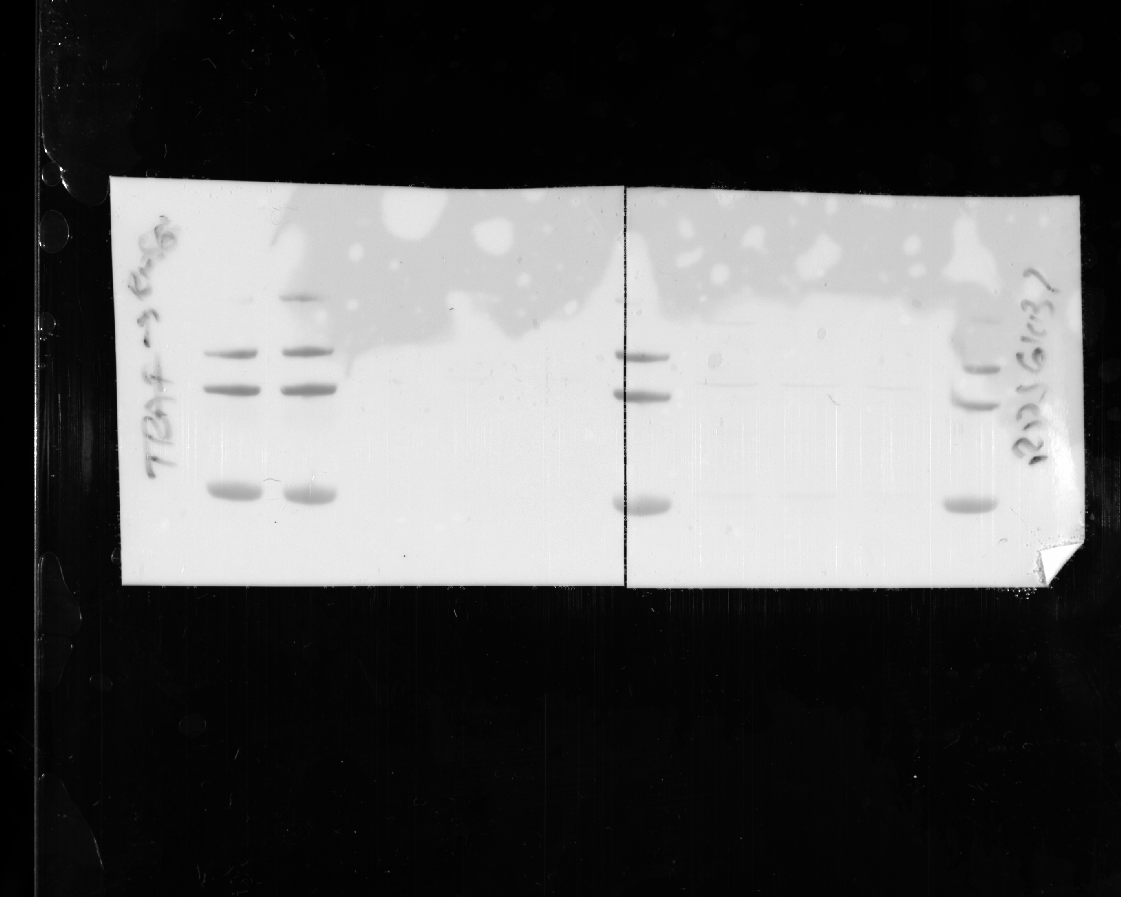

Supplement: Figure 2—source data 2. [file elife-105311-fig2-data2.zip › Figure 2 - Source Data 2/Fig2C_marker_for_tubulin_2024-04-19 15h40m17s 0.134s.tif]

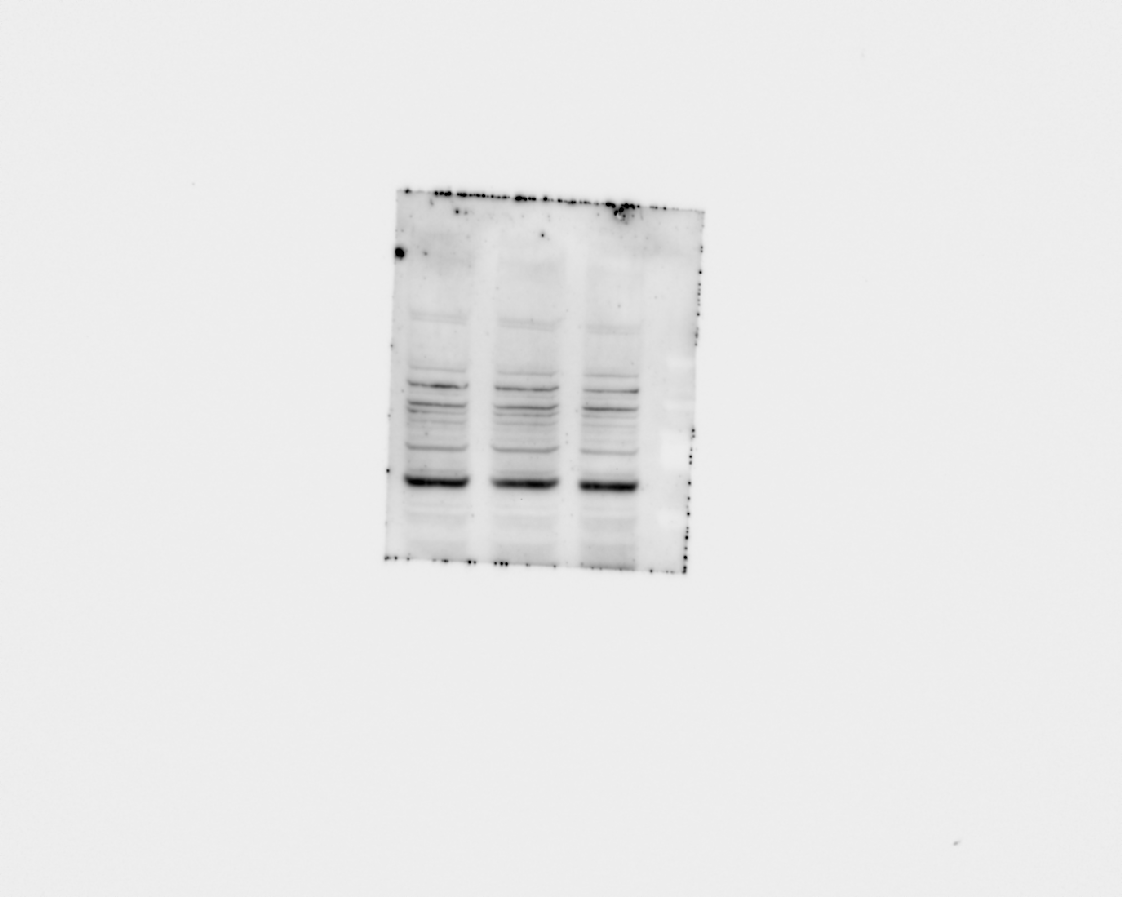

Supplement: Figure 2—source data 2. [file elife-105311-fig2-data2.zip › Figure 2 - Source Data 2/Fig2C_RPS6KB2_2024-04-26 13h53m15s 63.000s.tif]

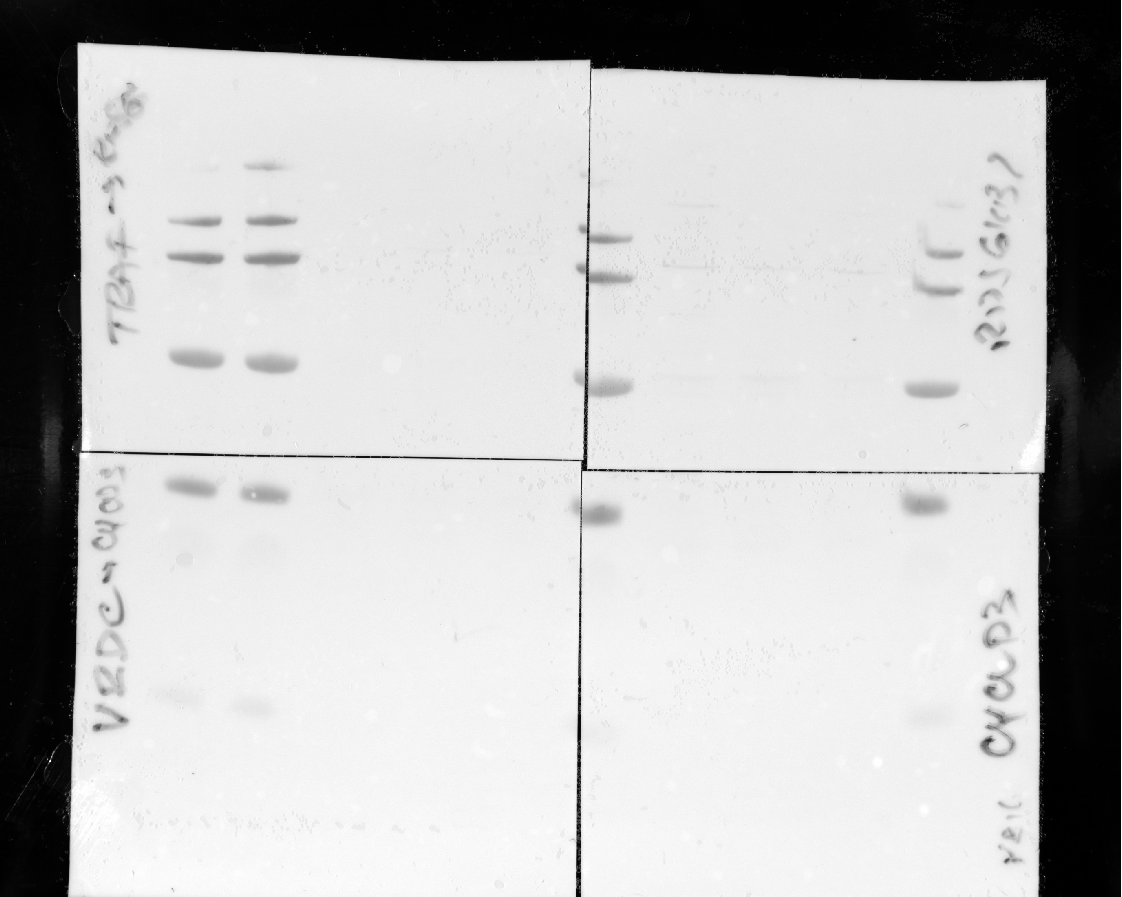

Supplement: Figure 2—source data 2. [file elife-105311-fig2-data2.zip › Figure 2 - Source Data 2/Fig2C_marker_for_eIF2A_2024-04-18 19h35m07s 0.169s.tif]

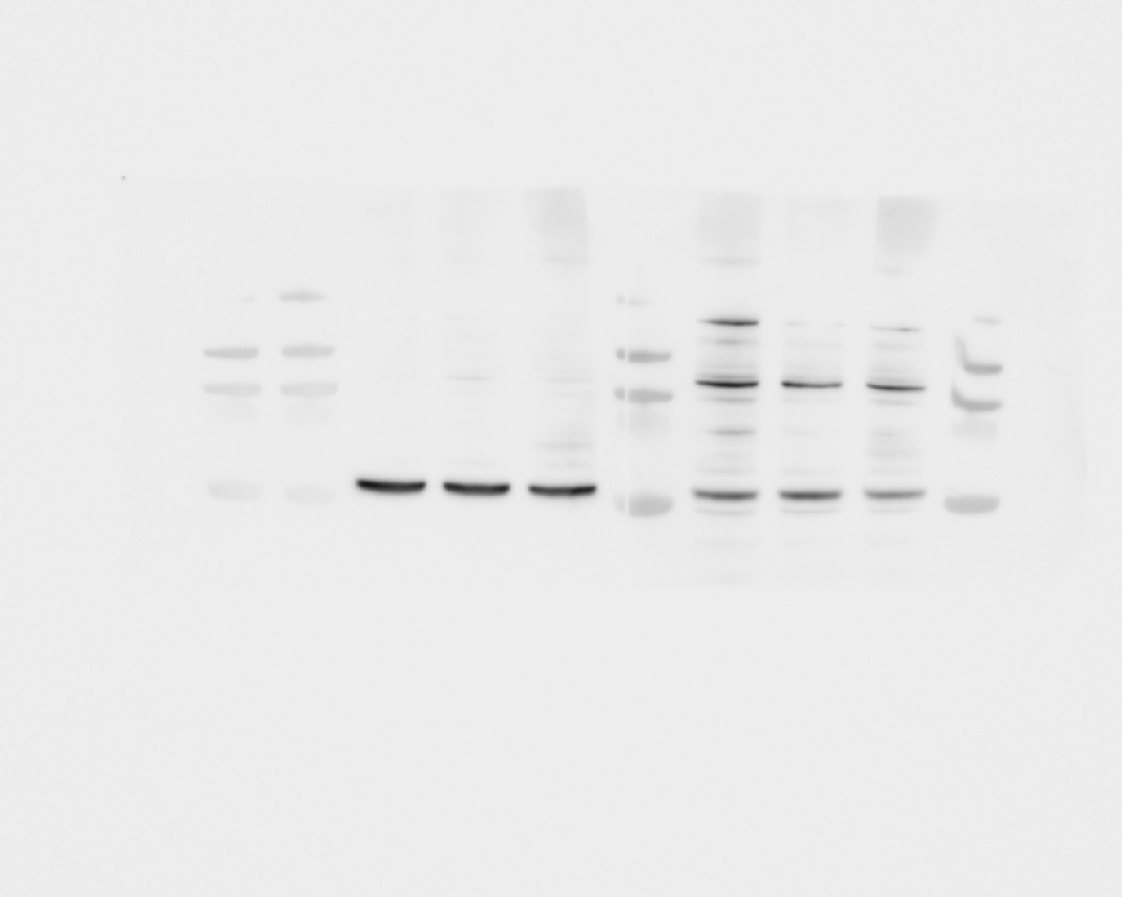

Supplement: Figure 2—source data 2. [file elife-105311-fig2-data2.zip › Figure 2 - Source Data 2/Fig2C_tubulin_2024-04-19 15h39m32s 13.000s.tif]

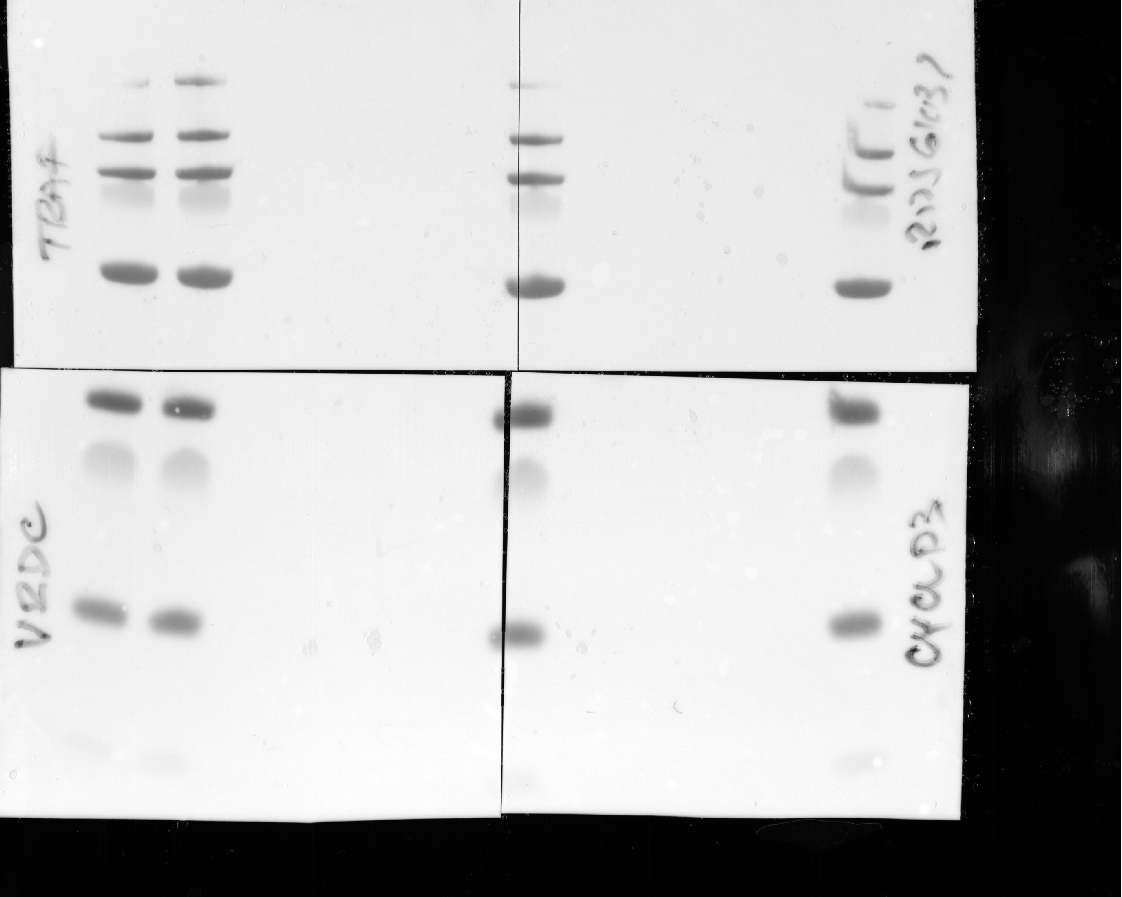

Supplement: Figure 2—source data 2. [file elife-105311-fig2-data2.zip › Figure 2 - Source Data 2/Fig2C_marker_for_CCND3_2024-04-16 14h31m42s 0.173s.tif]

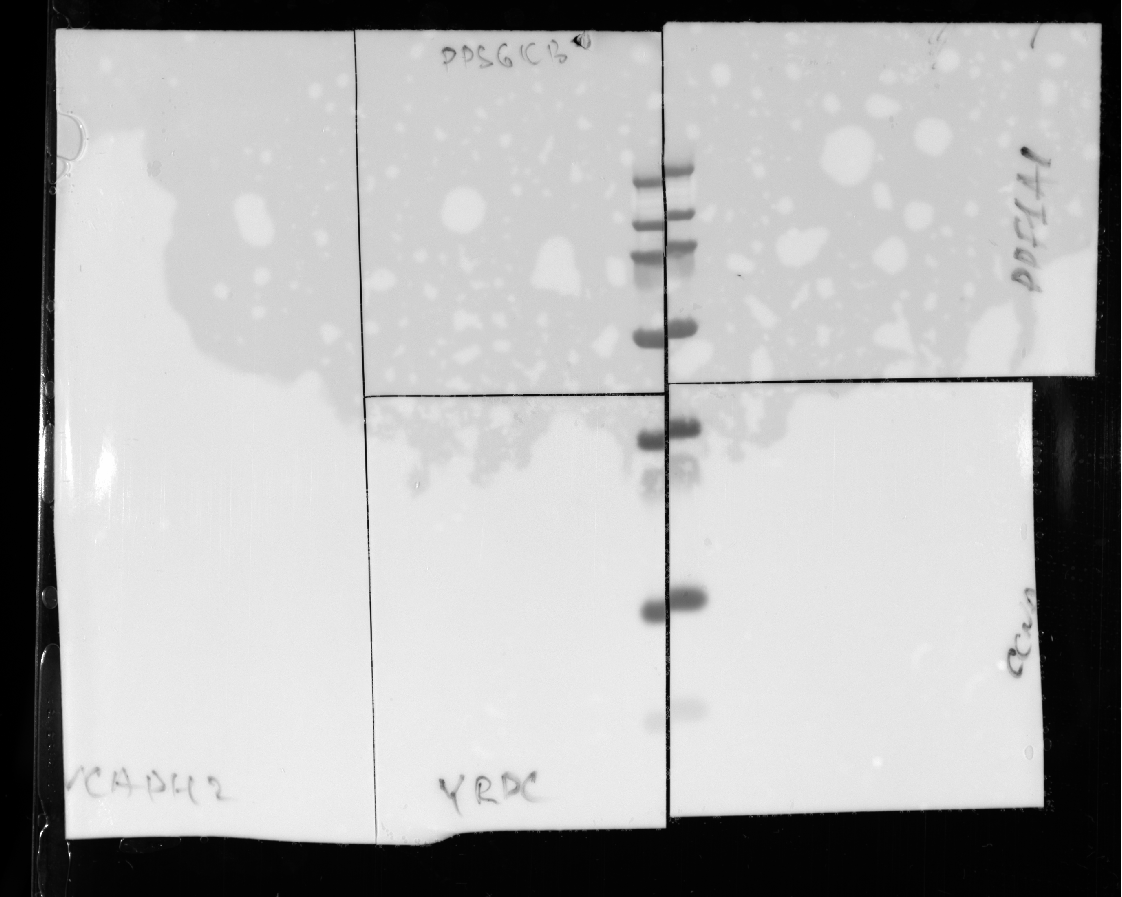

Supplement: Figure 2—source data 2. [file elife-105311-fig2-data2.zip › Figure 2 - Source Data 2/Fig2B_marker_for_NCAPH2_PPF1A1_2024-04-26 13h44m14s 0.071s.tif]

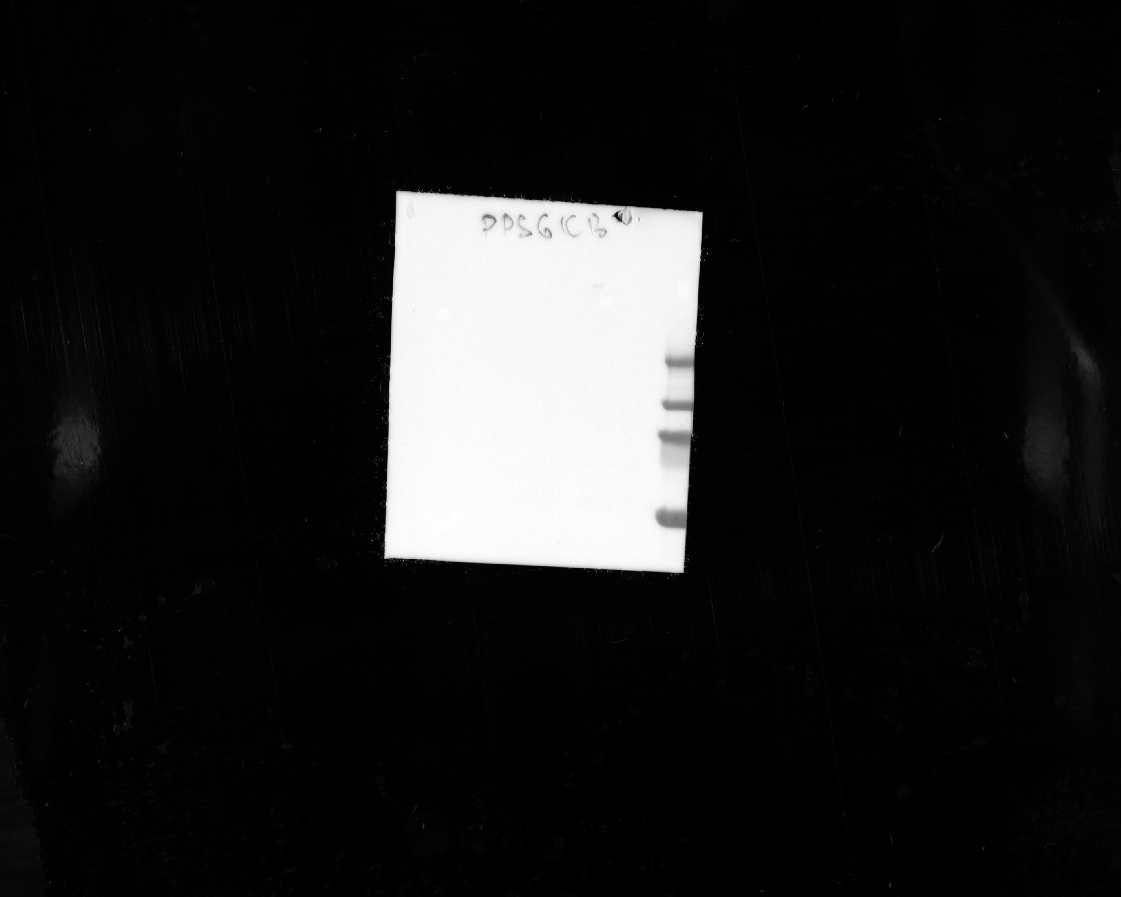

Supplement: Figure 2—source data 2. [file elife-105311-fig2-data2.zip › Figure 2 - Source Data 2/Fig2C_marker_for_RPS6KB2_2024-04-26 13h58m46s 0.192s.tif]

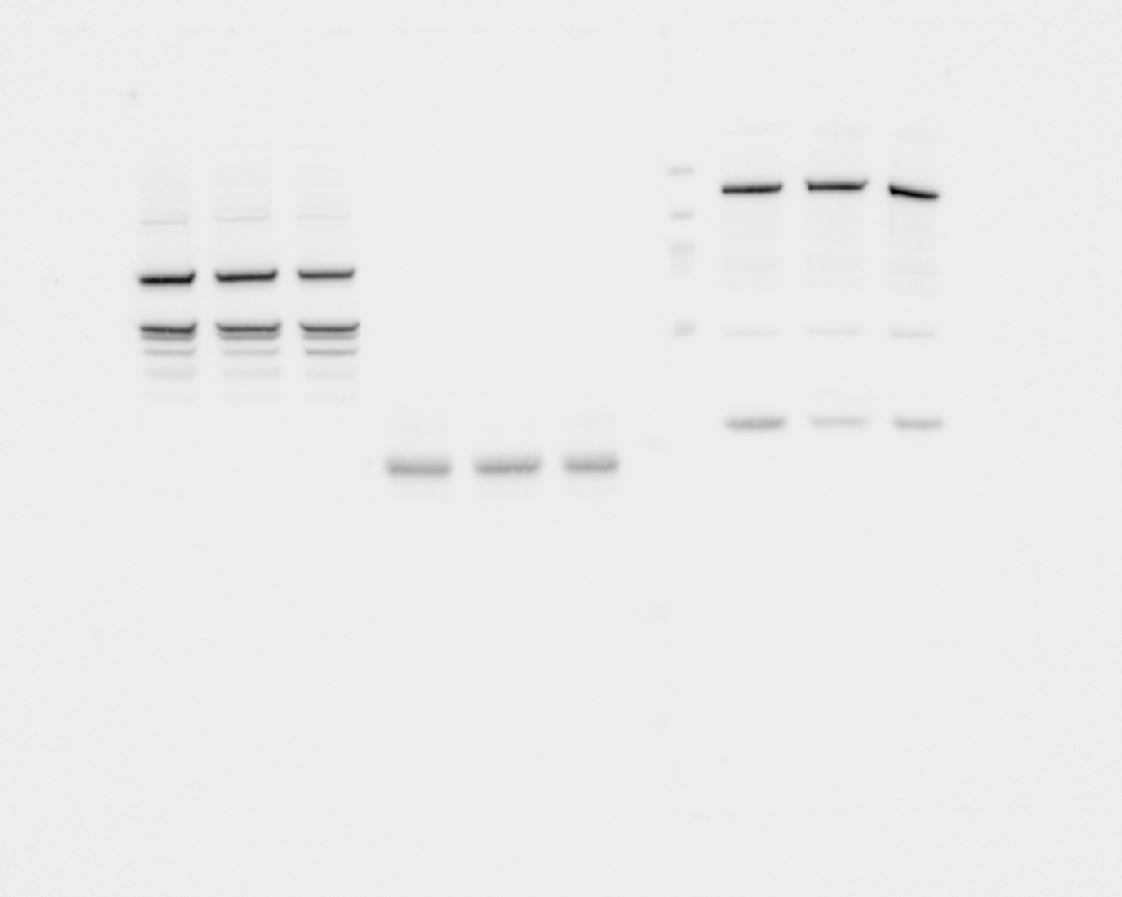

Supplement: Figure 2—source data 2. [file elife-105311-fig2-data2.zip › Figure 2 - Source Data 2/Fig2B_NCAPH2_PPF1A1_2024-04-26 13h40m30s 4.428s.tif]

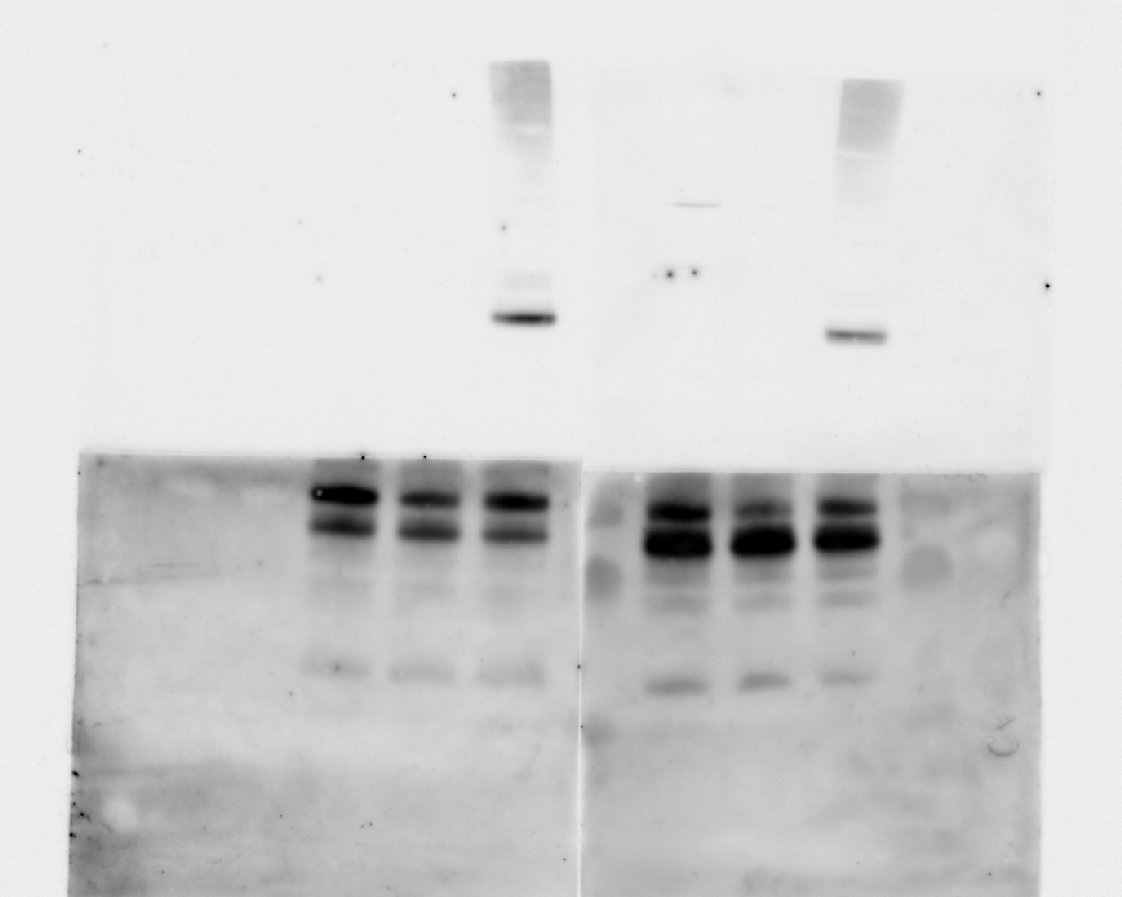

Supplement: Figure 2—source data 2. [file elife-105311-fig2-data2.zip › Figure 2 - Source Data 2/Fig2C_eIF2A_2024-04-18 19h29m20s 74.998s.tif]

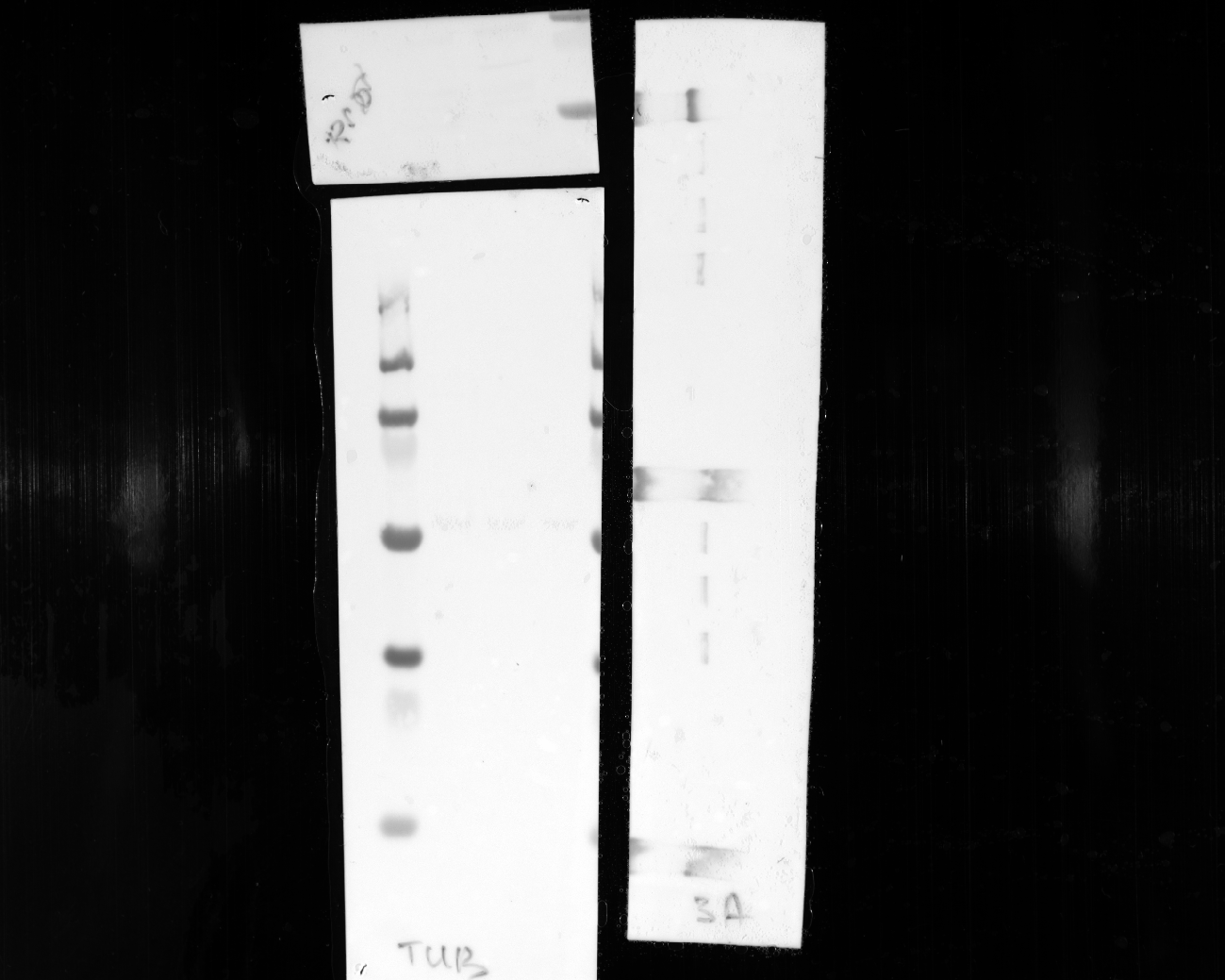

Supplement: Figure 2—figure supplement 2—source data 2. [file elife-105311-fig2-figsupp2-data2.zip › Figure 2 - Figure Supplement 2 - Source Data 2/Fig2_sup2B_marker_for_eIF2A_HSP90_2024-05-08 15h22m59s 0.180s.tif]

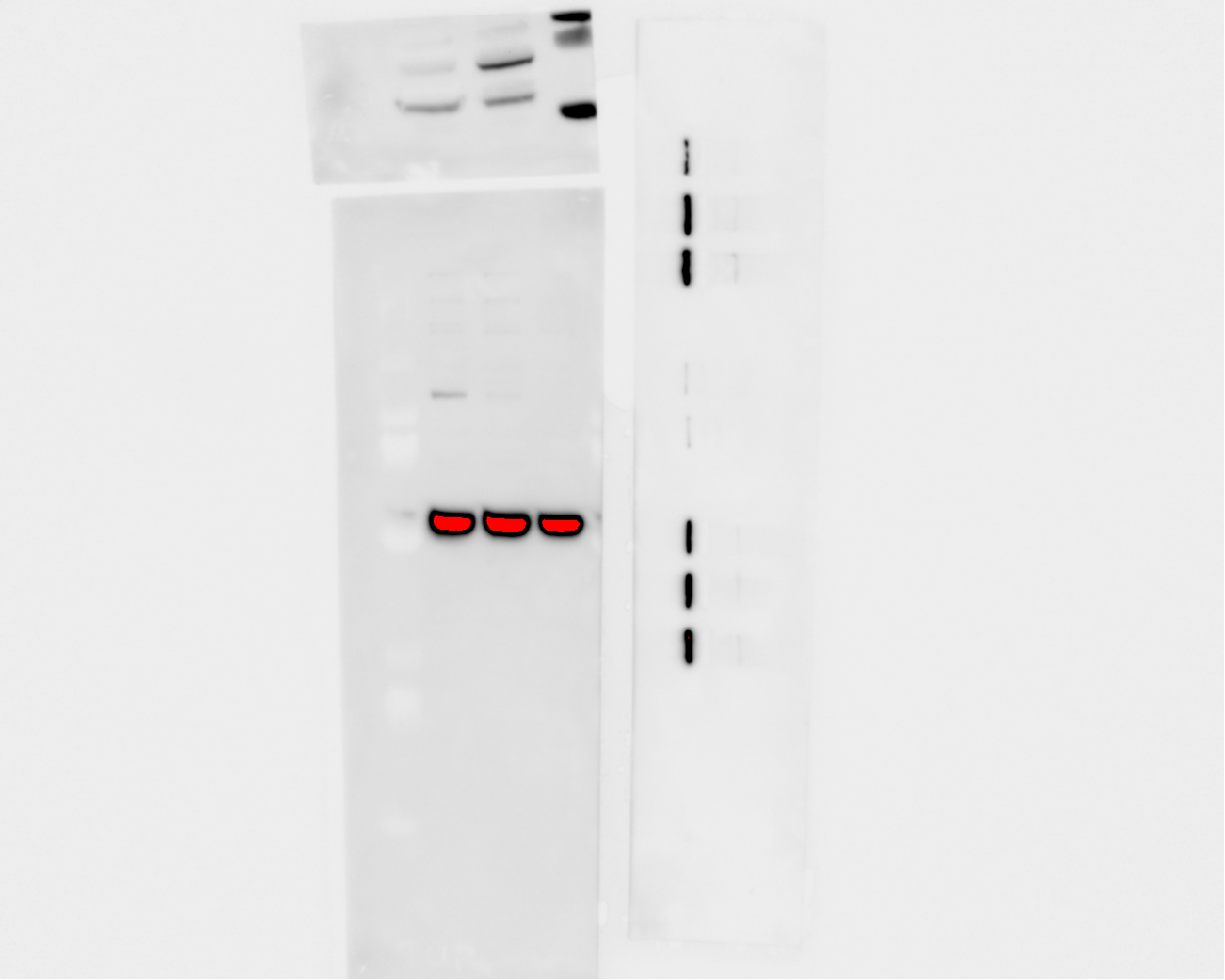

Supplement: Figure 2—figure supplement 2—source data 2. [file elife-105311-fig2-figsupp2-data2.zip › Figure 2 - Figure Supplement 2 - Source Data 2/Fig2_sup2B_eIF2A_HSP90_2024-05-08 15h16m20s 7.000s.tif]

## Figure 3

**B**

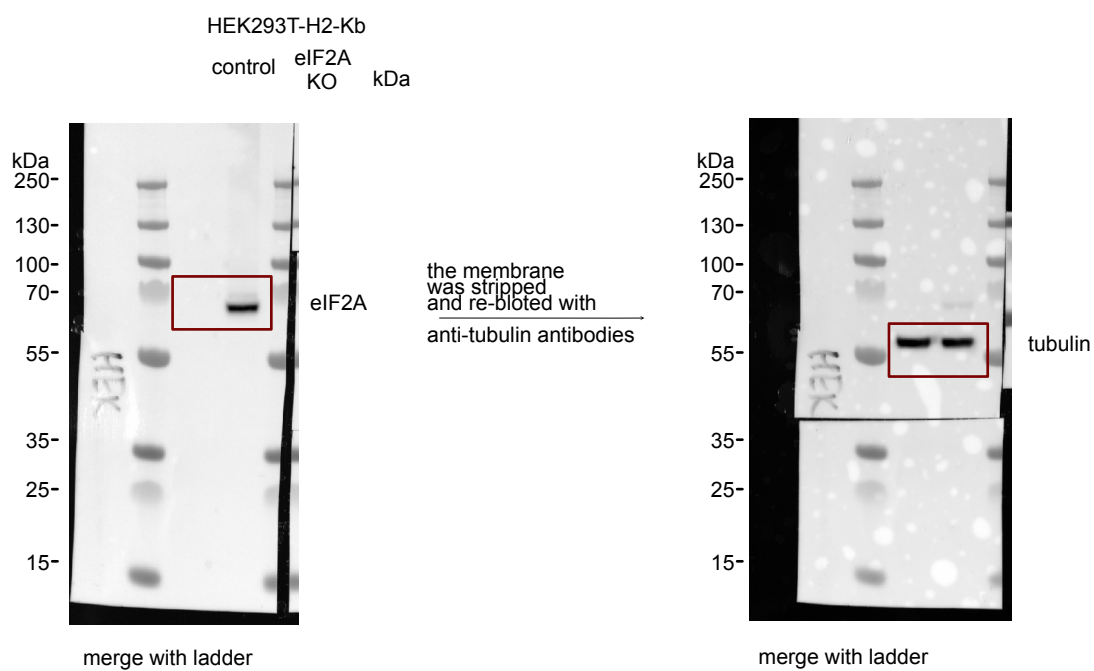

Supplement: Figure 3—source data 1. [file elife-105311-fig3-data1.pdf]

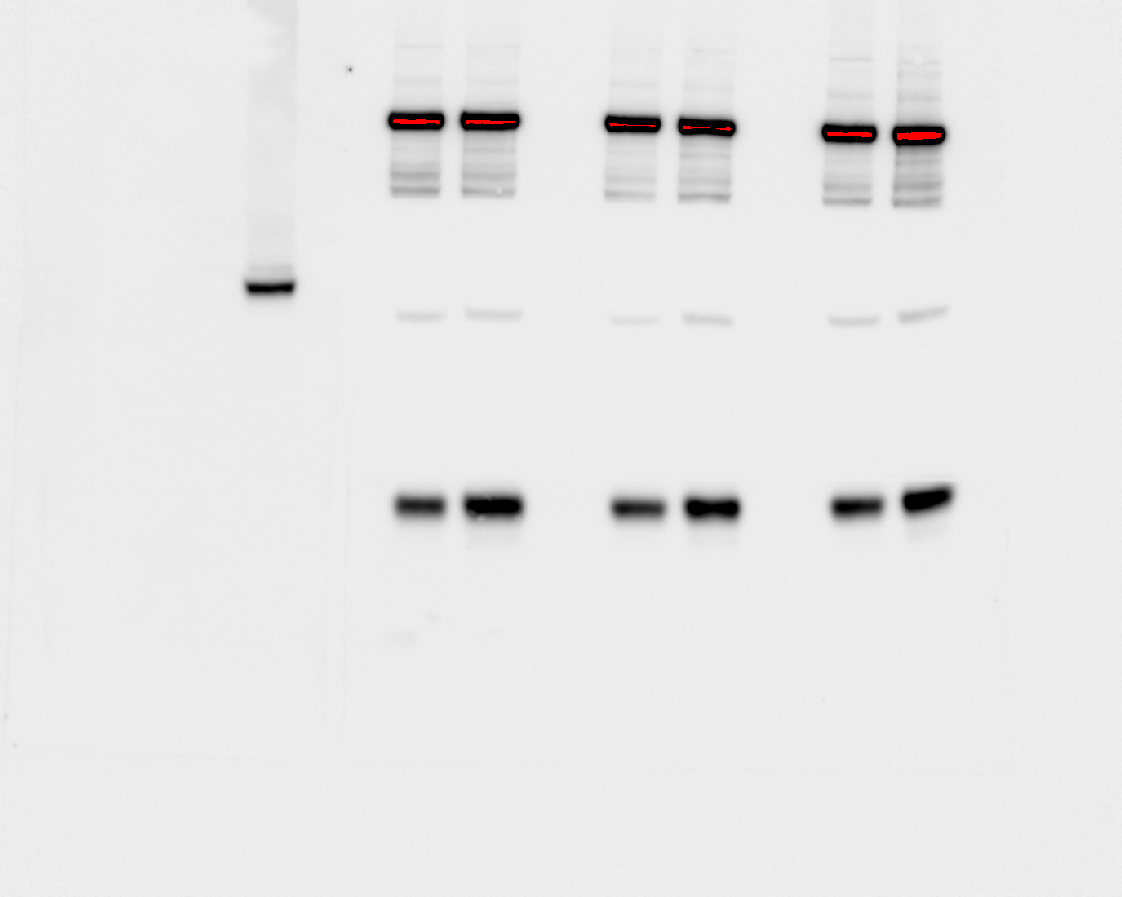

Supplement: Figure 3—source data 2. [file elife-105311-fig3-data2.zip › Figure 3 - Source Data 2/Fig3B_eIF2A_2024-05-19 18h47m25s 10.000s.tif]

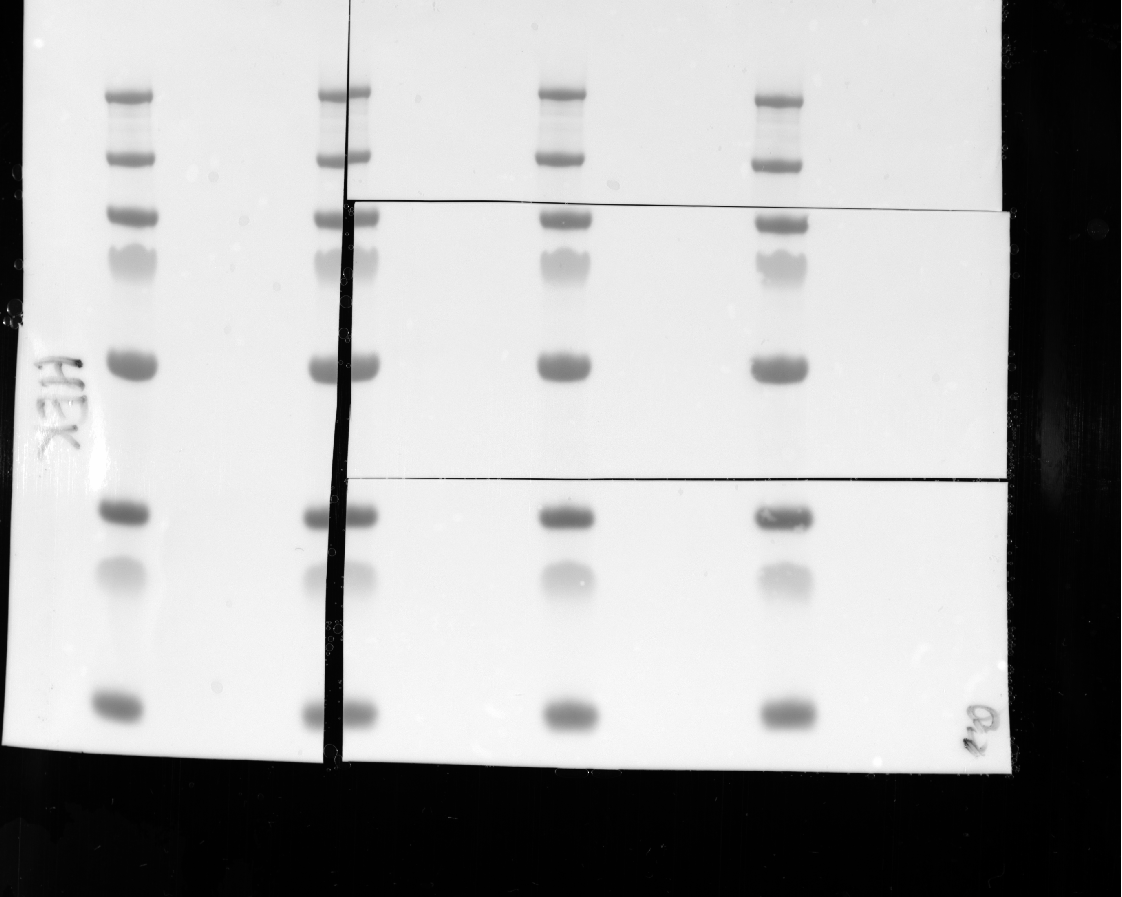

Supplement: Figure 3—source data 2. [file elife-105311-fig3-data2.zip › Figure 3 - Source Data 2/Fig3B_marker_for_eIF2A_2024-05-19 18h52m08s 0.157s.tif]

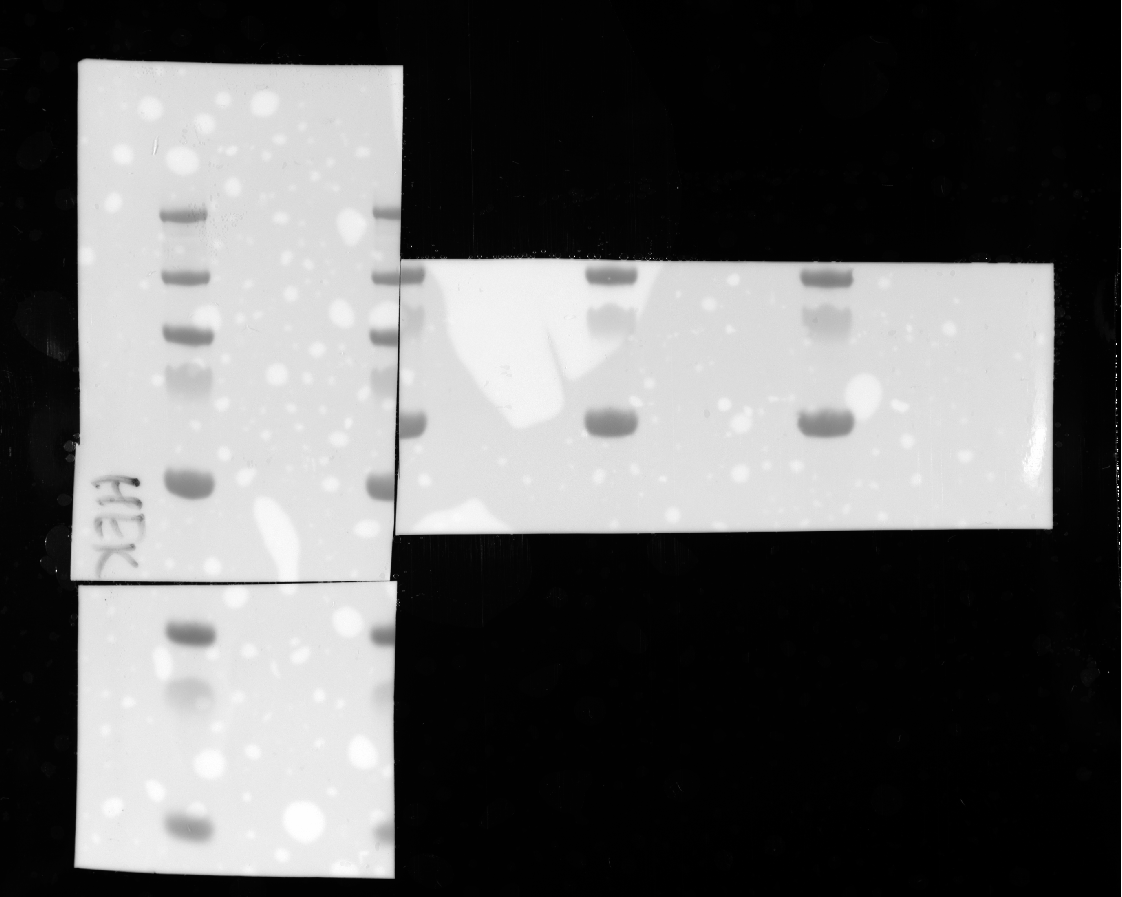

Supplement: Figure 3—source data 2. [file elife-105311-fig3-data2.zip › Figure 3 - Source Data 2/Fig3B_marker_for_tubulin_2024-05-20 21h46m13s 0.179s.tif]

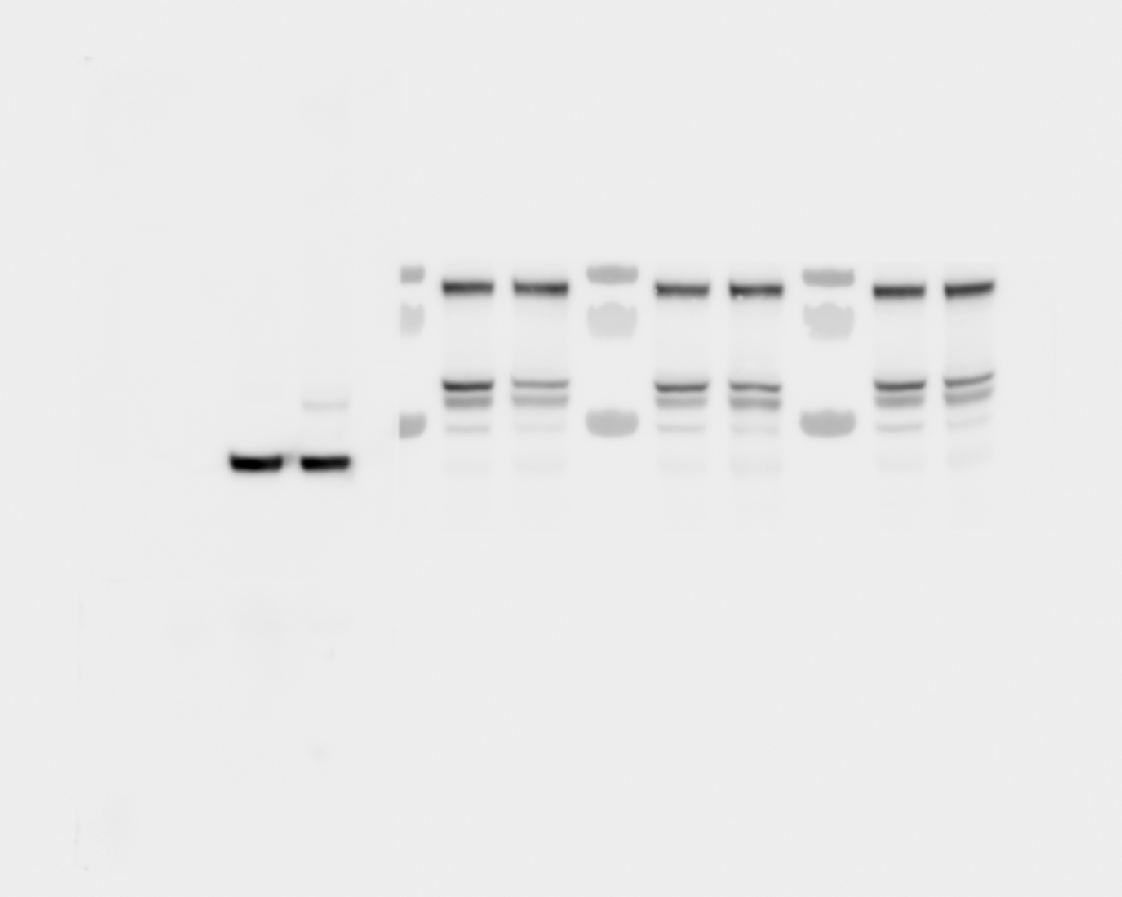

Supplement: Figure 3—source data 2. [file elife-105311-fig3-data2.zip › Figure 3 - Source Data 2/Fig3B_tubulin_2024-05-20 21h38m48s 1.000s.tif]

Fig3 Suppl. Figure 1G

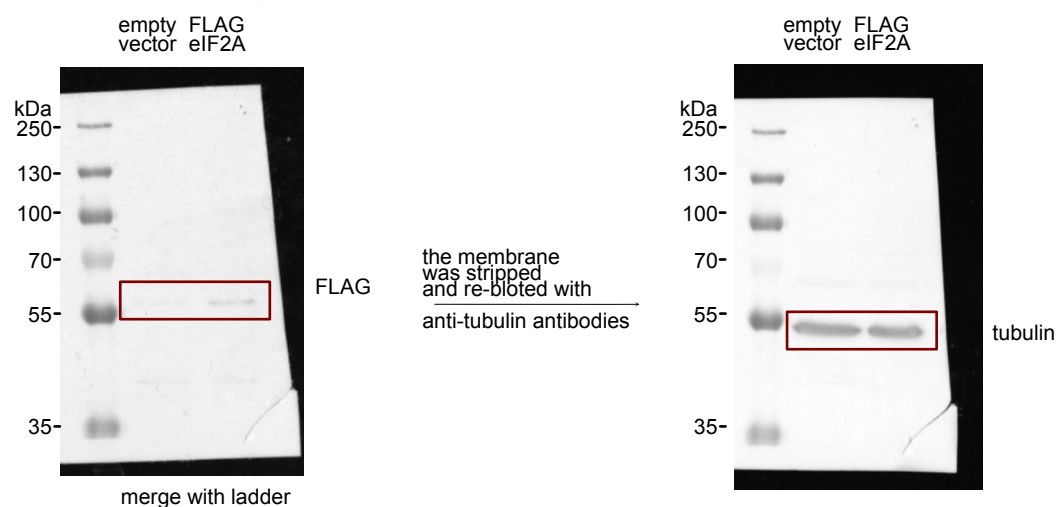

Supplement: Figure 3—figure supplement 1—source data 1. [file elife-105311-fig3-figsupp1-data1.pdf]

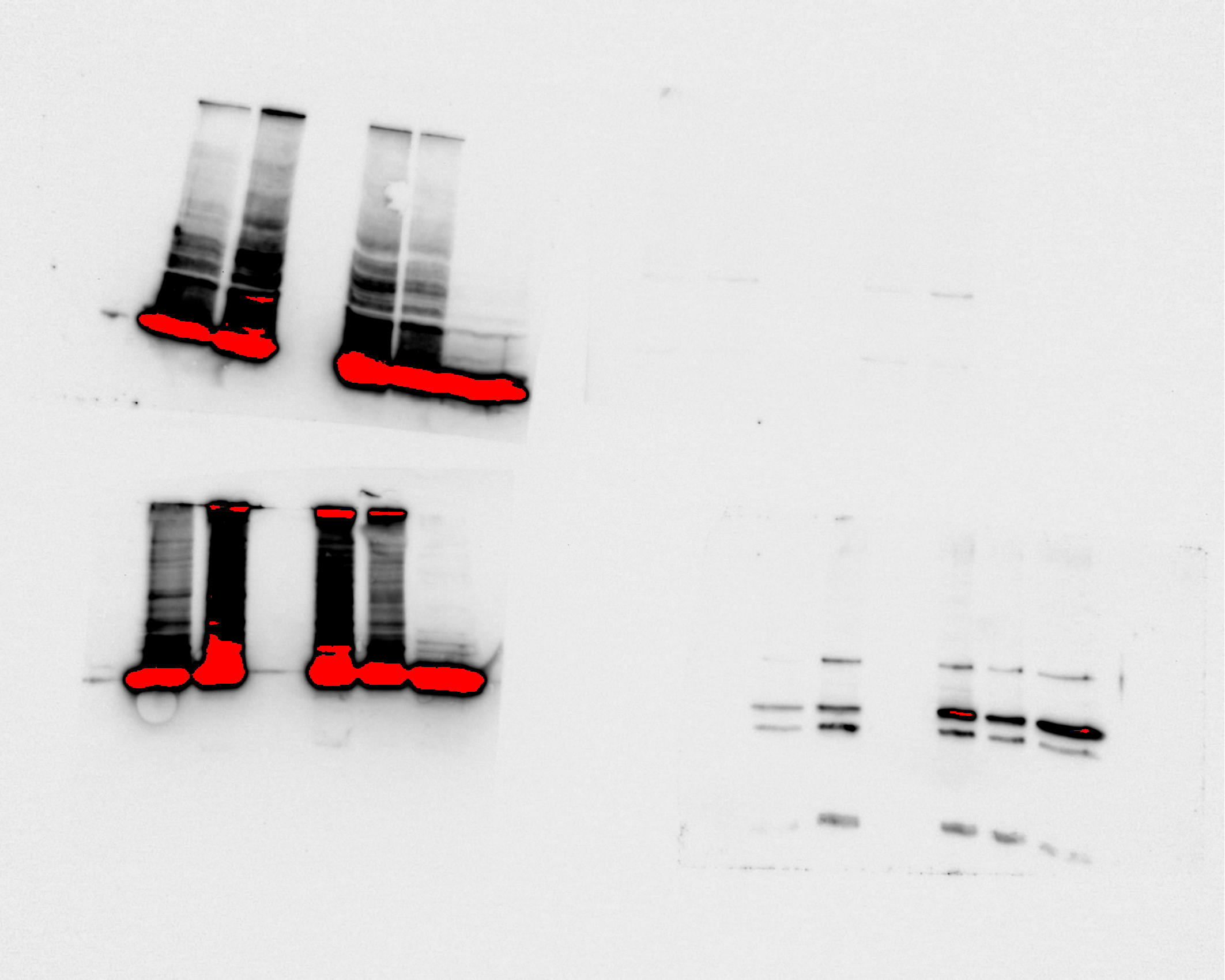

Supplement: Figure 3—figure supplement 1—source data 2. [file elife-105311-fig3-figsupp1-data2.zip › Figure 3 - Figure Supplement 1 - Source Data 2/Fig3_sup1G_Flag_2025-01-20 11h35m51s 119.996s.tif]

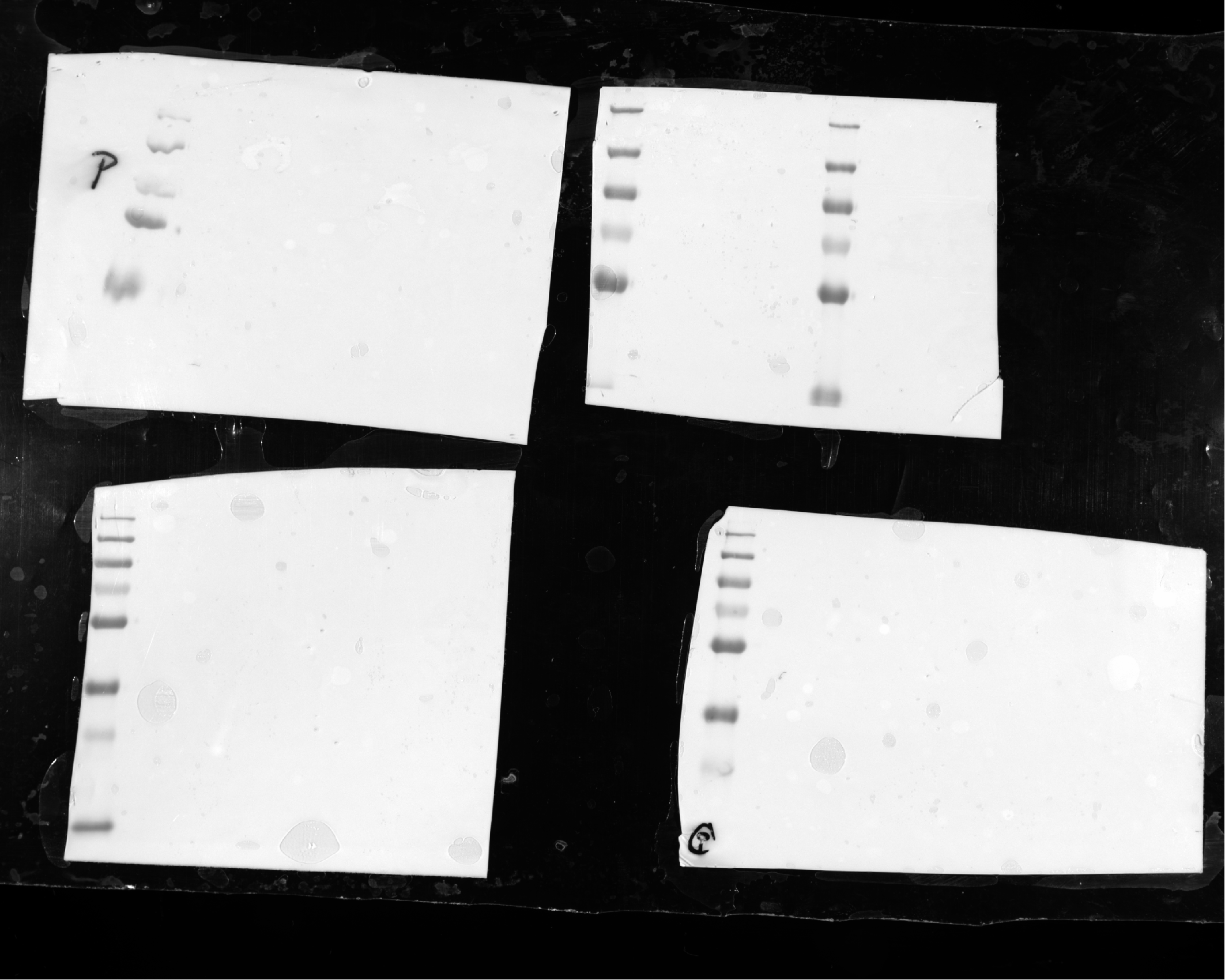

Supplement: Figure 3—figure supplement 1—source data 2. [file elife-105311-fig3-figsupp1-data2.zip › Figure 3 - Figure Supplement 1 - Source Data 2/Fig3_sup1G_marker_for_Flag_2025-01-20 11h36m53s 0.239s.tif]

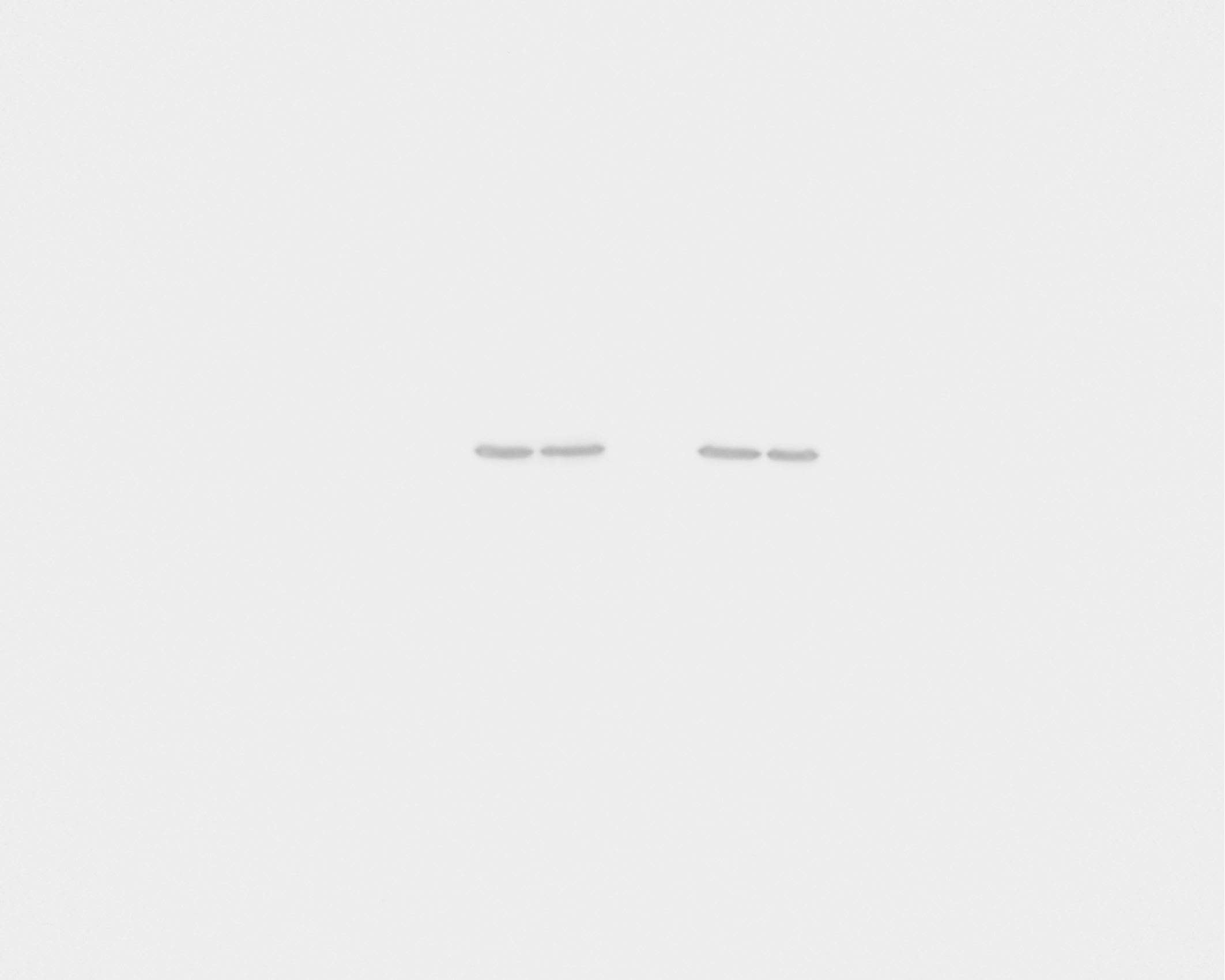

Supplement: Figure 3—figure supplement 1—source data 2. [file elife-105311-fig3-figsupp1-data2.zip › Figure 3 - Figure Supplement 1 - Source Data 2/Fig3_sup1G_tubulin_2025-01-28 12h01m12s 3.666s.tif]

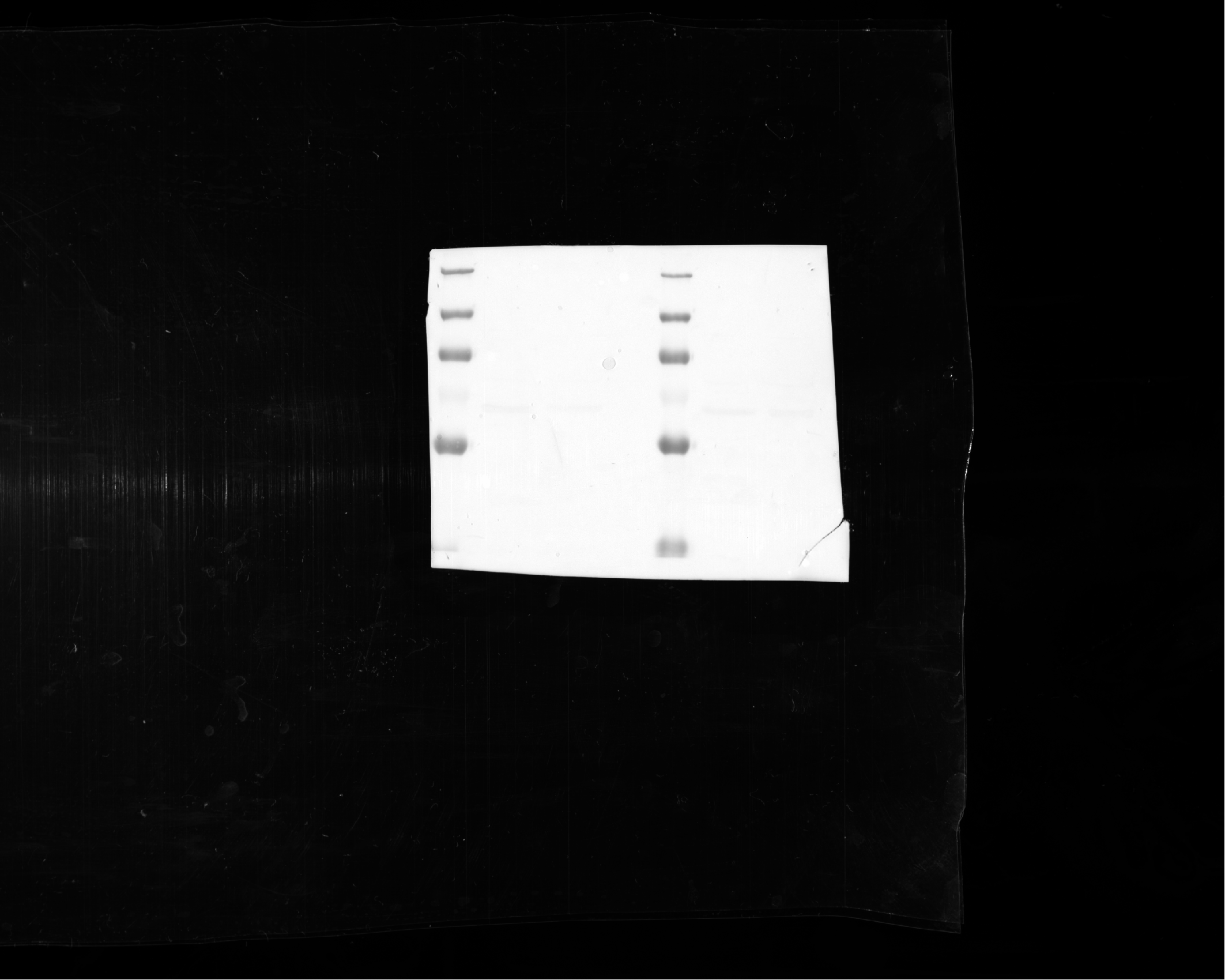

Supplement: Figure 3—figure supplement 1—source data 2. [file elife-105311-fig3-figsupp1-data2.zip › Figure 3 - Figure Supplement 1 - Source Data 2/Fig3_sup1G_marker_for_tubulin_2025-01-28 12h02m27s 0.276s.tif]

# Fig.4. Suppl. Figure 1D

same samples were run in a parallel:

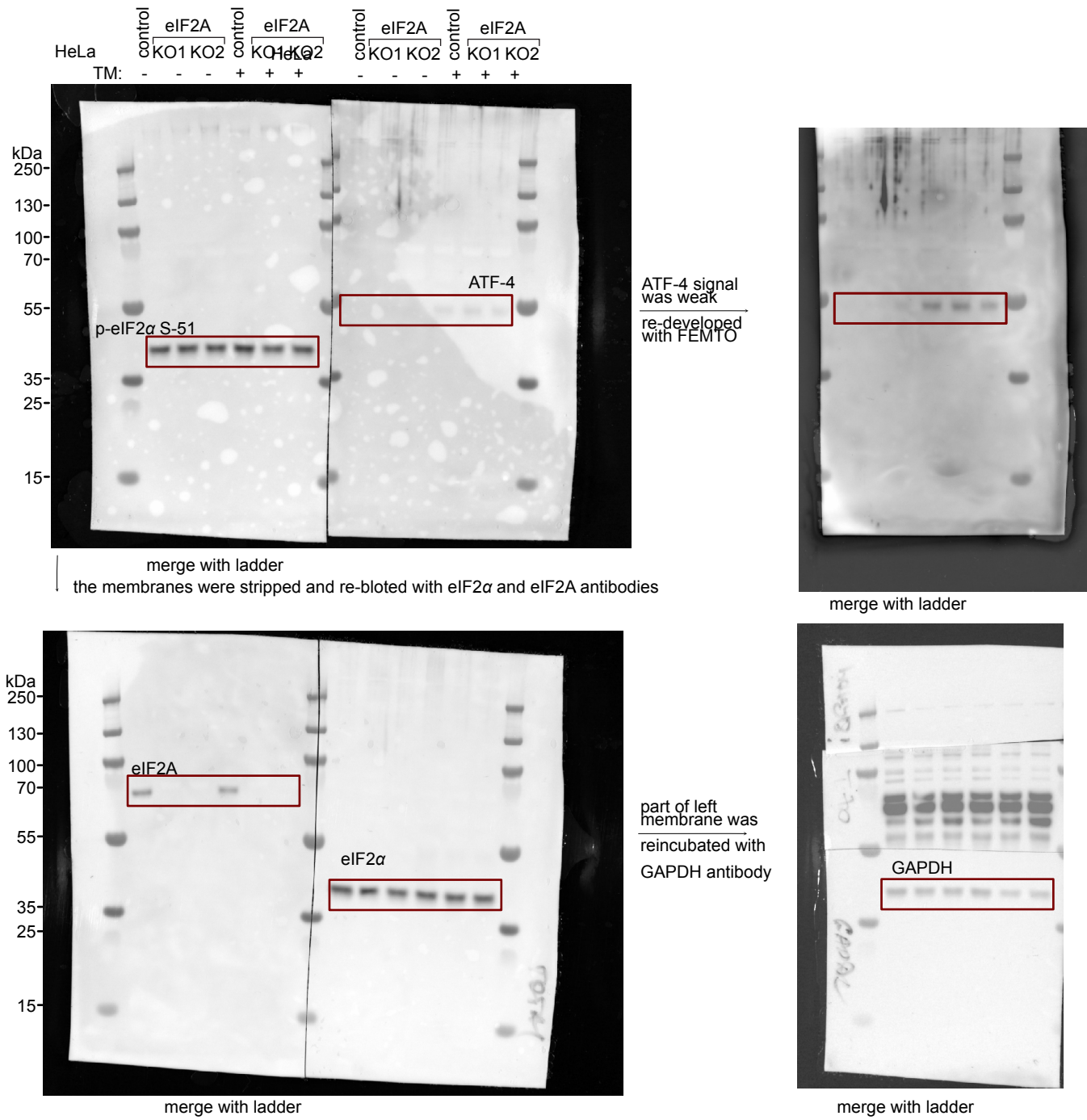

Supplement: Figure 4—figure supplement 1—source data 1. [file elife-105311-fig4-figsupp1-data1.pdf]

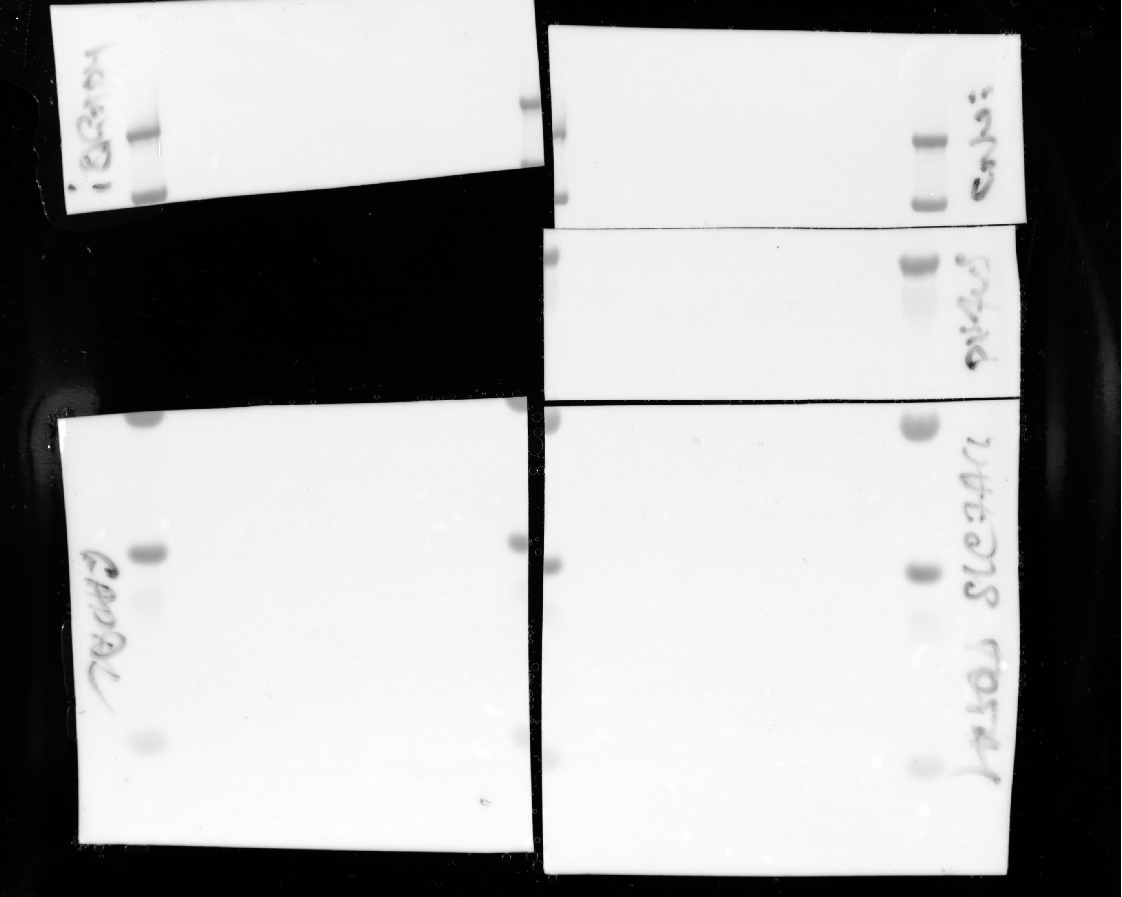

Supplement: Figure 4—figure supplement 1—source data 2. [file elife-105311-fig4-figsupp1-data2.zip › Figure 4 - Figure Supplement 1 - Source Data 2/Fig4_sup1D_marker_for_GAPDH_2024-05-16 13h35m18s 0.169s.tif]

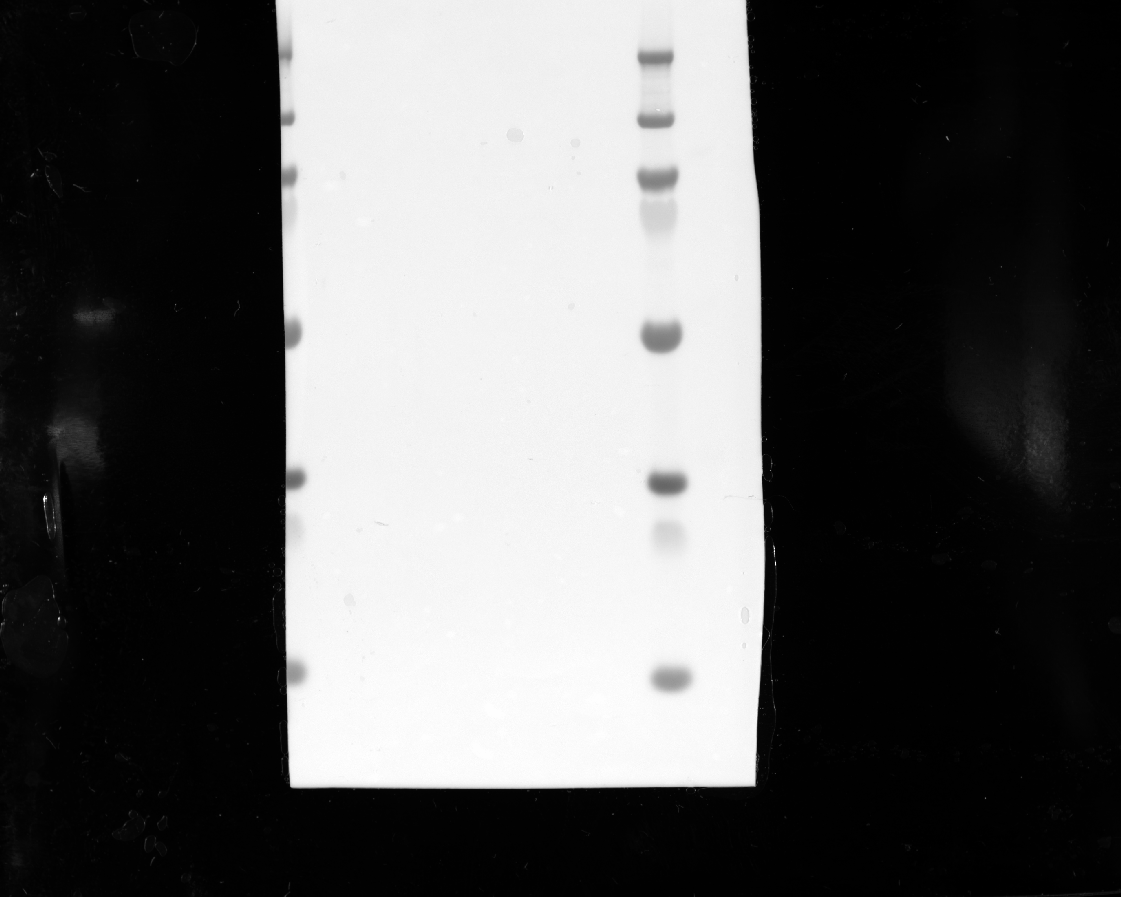

Supplement: Figure 4—figure supplement 1—source data 2. [file elife-105311-fig4-figsupp1-data2.zip › Figure 4 - Figure Supplement 1 - Source Data 2/Fig4_sup1D_marker_for_ATF4_2024-05-13 17h07m50s 0.186s.tif]

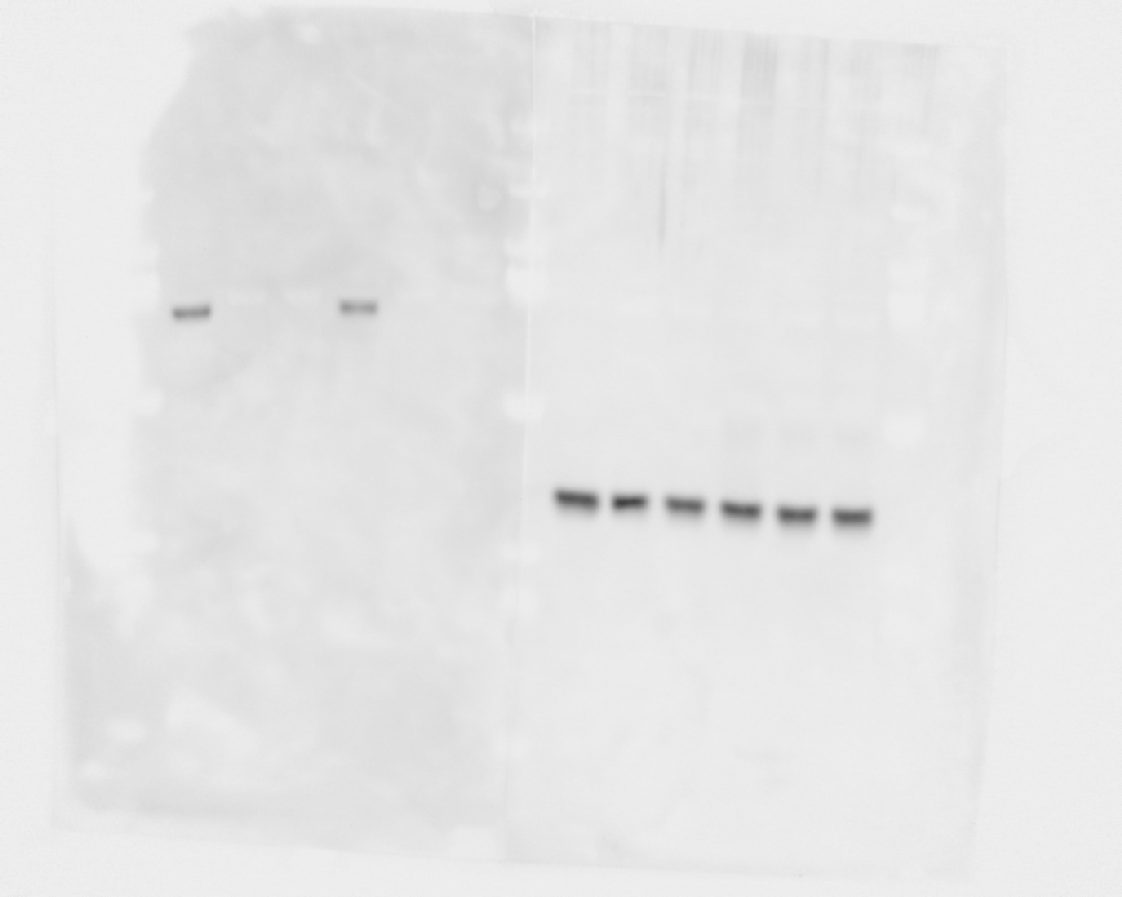

Supplement: Figure 4—figure supplement 1—source data 2. [file elife-105311-fig4-figsupp1-data2.zip › Figure 4 - Figure Supplement 1 - Source Data 2/Fig4_sup1D_eIF2alpha_eIF2A_2024-05-14 13h30m06s 13.000s.tif]

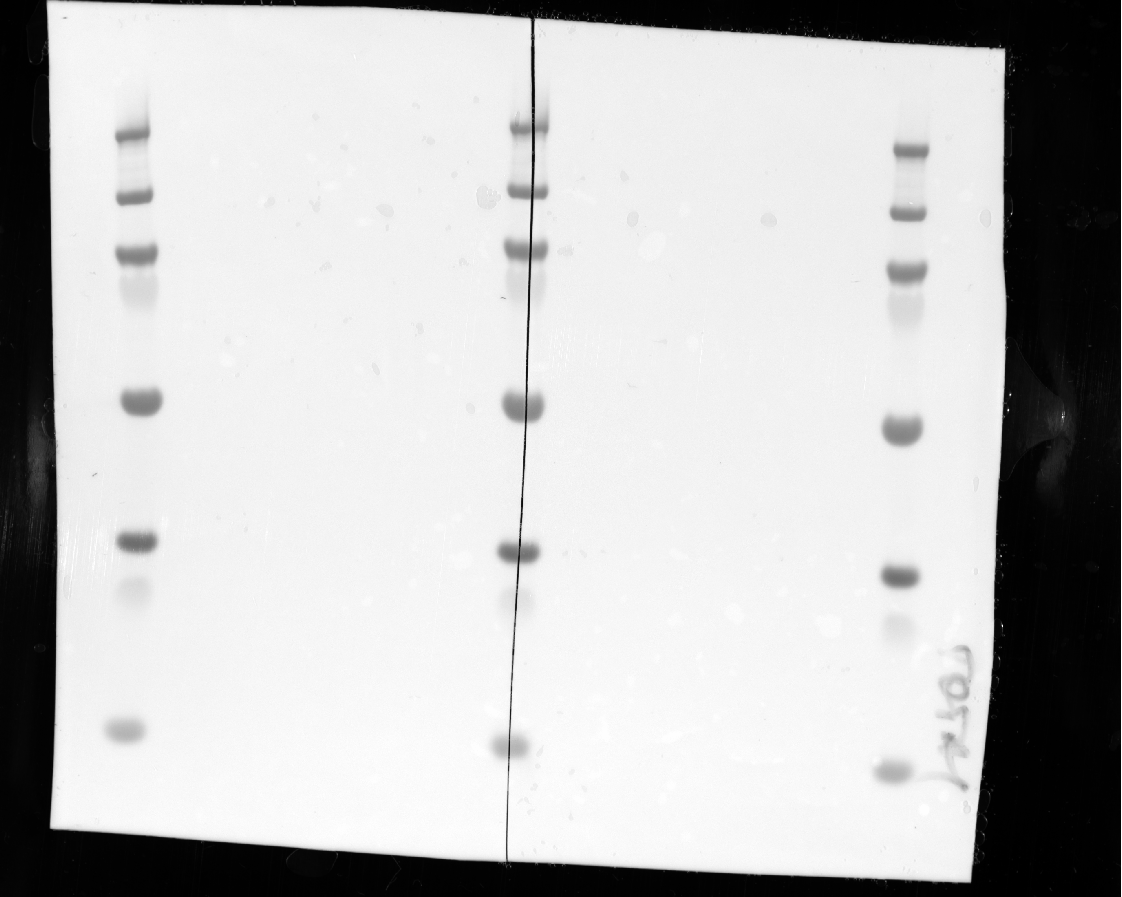

Supplement: Figure 4—figure supplement 1—source data 2. [file elife-105311-fig4-figsupp1-data2.zip › Figure 4 - Figure Supplement 1 - Source Data 2/Fig4_sup1D_marker_for_eIF2alpha_eIF2A_2024-05-14 13h37m16s 0.172s.tif]

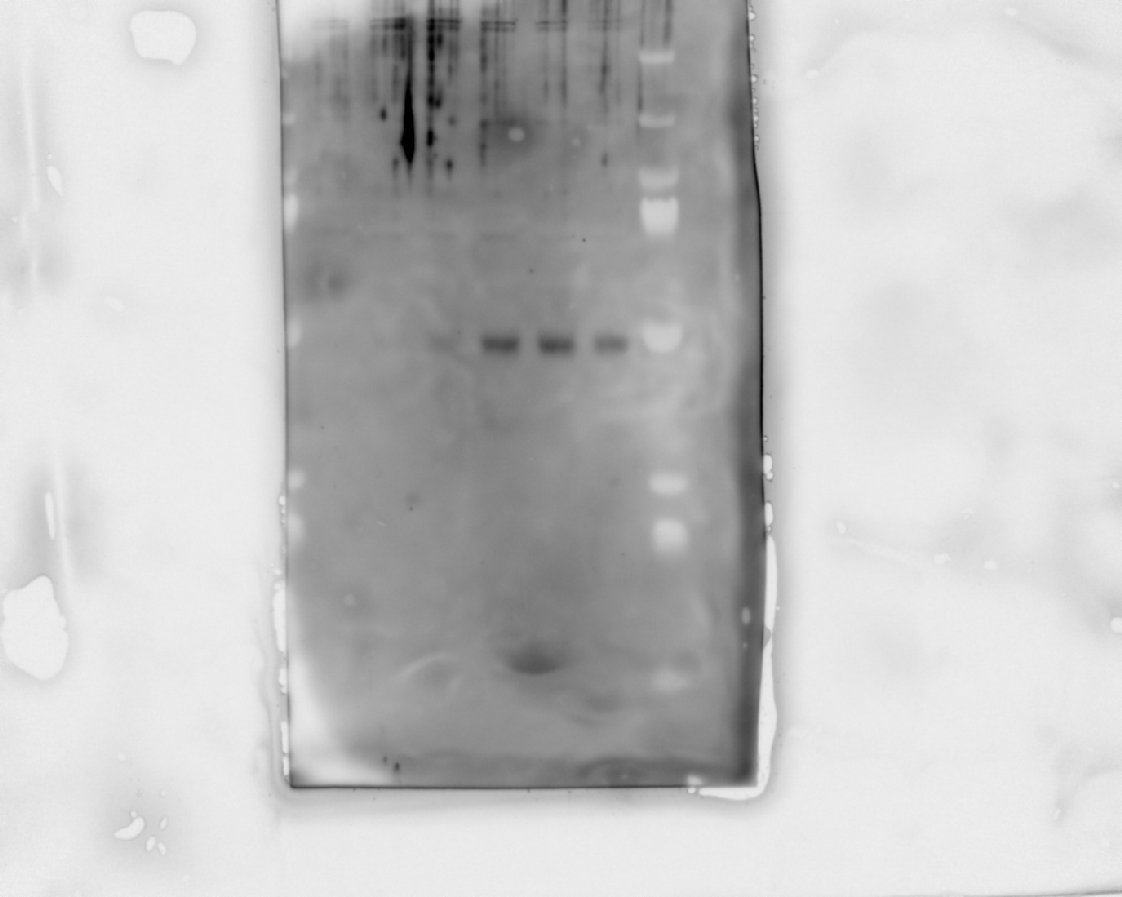

Supplement: Figure 4—figure supplement 1—source data 2. [file elife-105311-fig4-figsupp1-data2.zip › Figure 4 - Figure Supplement 1 - Source Data 2/Fig4_sup1D_ATF4_2024-05-13 17h06m45s 10.000s.tif]

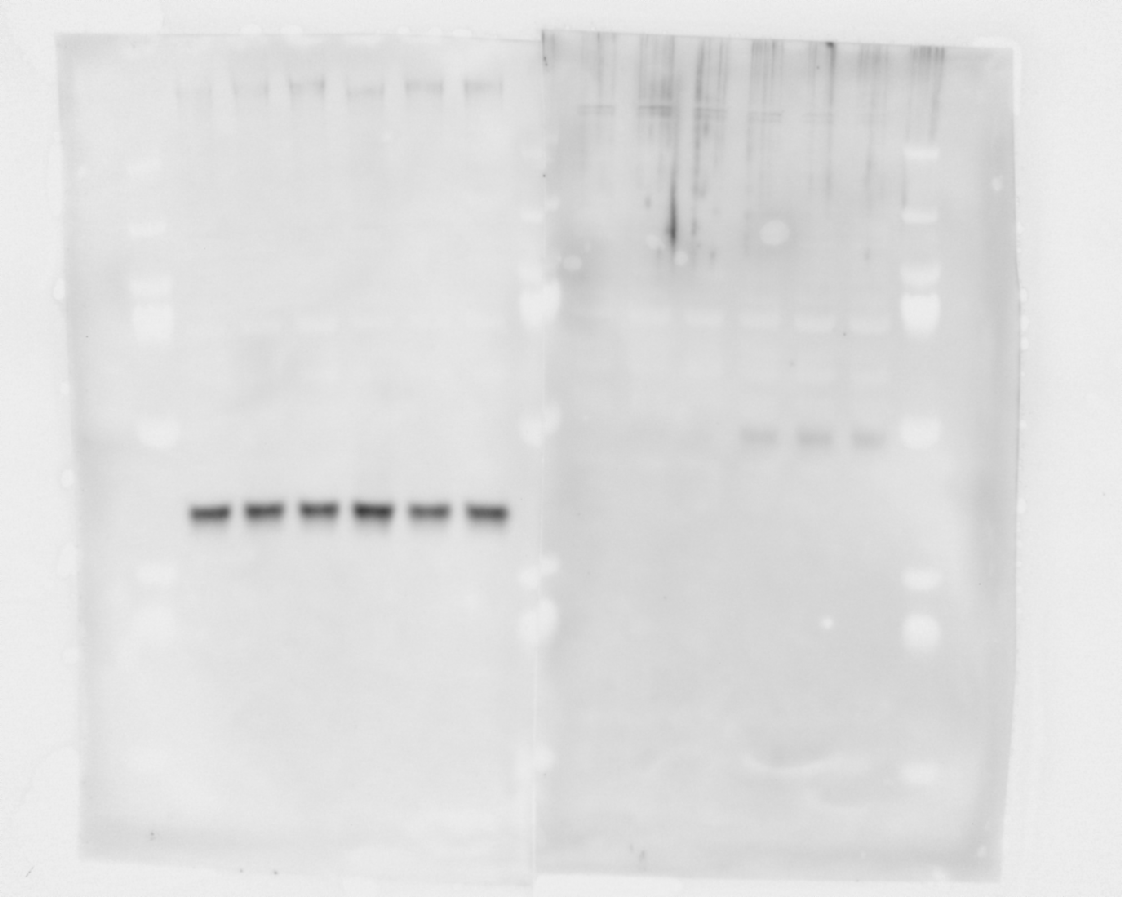

Supplement: Figure 4—figure supplement 1—source data 2. [file elife-105311-fig4-figsupp1-data2.zip › Figure 4 - Figure Supplement 1 - Source Data 2/Fig4_sup1D_p_eIF2alpha_2024-05-13 16h53m14s 16.498s.tif]

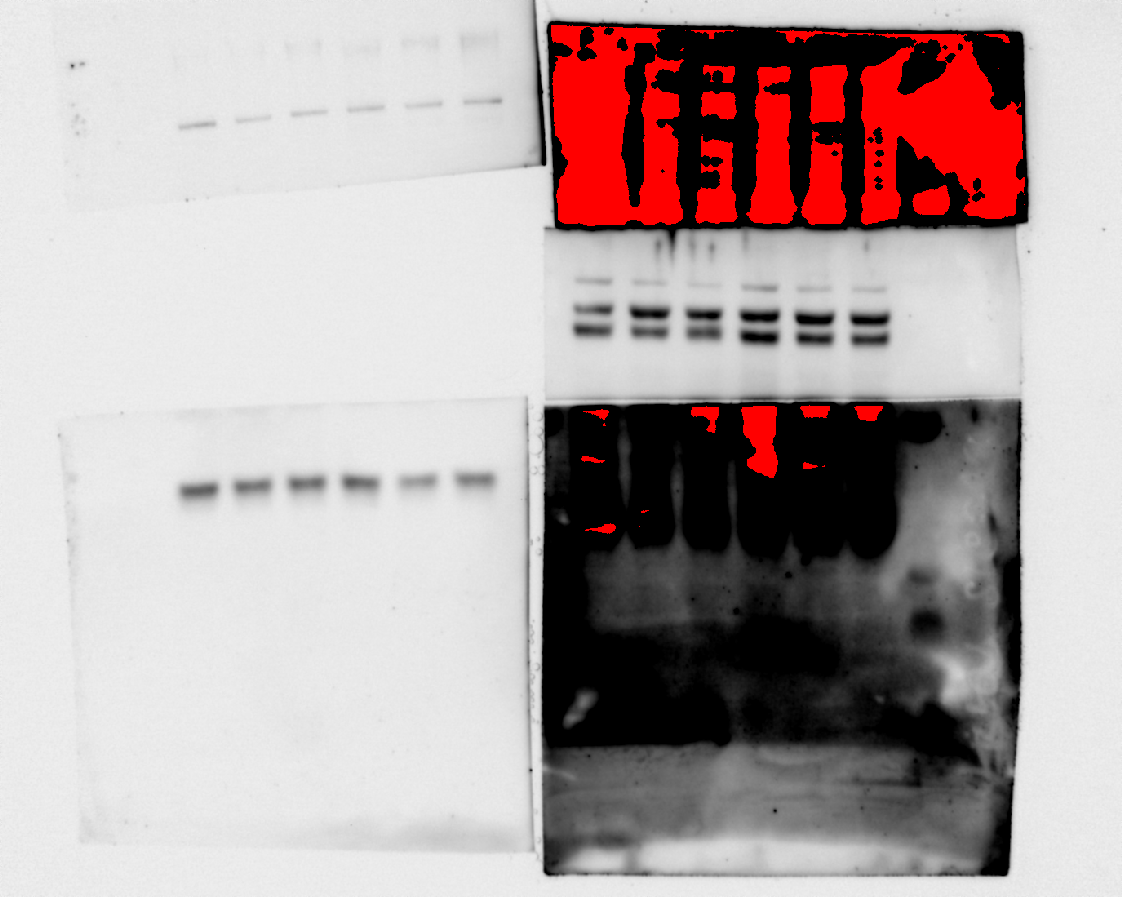

Supplement: Figure 4—figure supplement 1—source data 2. [file elife-105311-fig4-figsupp1-data2.zip › Figure 4 - Figure Supplement 1 - Source Data 2/Fig4_sup1D_GAPDH_2024-05-16 13h34m16s 45.250s.tif]

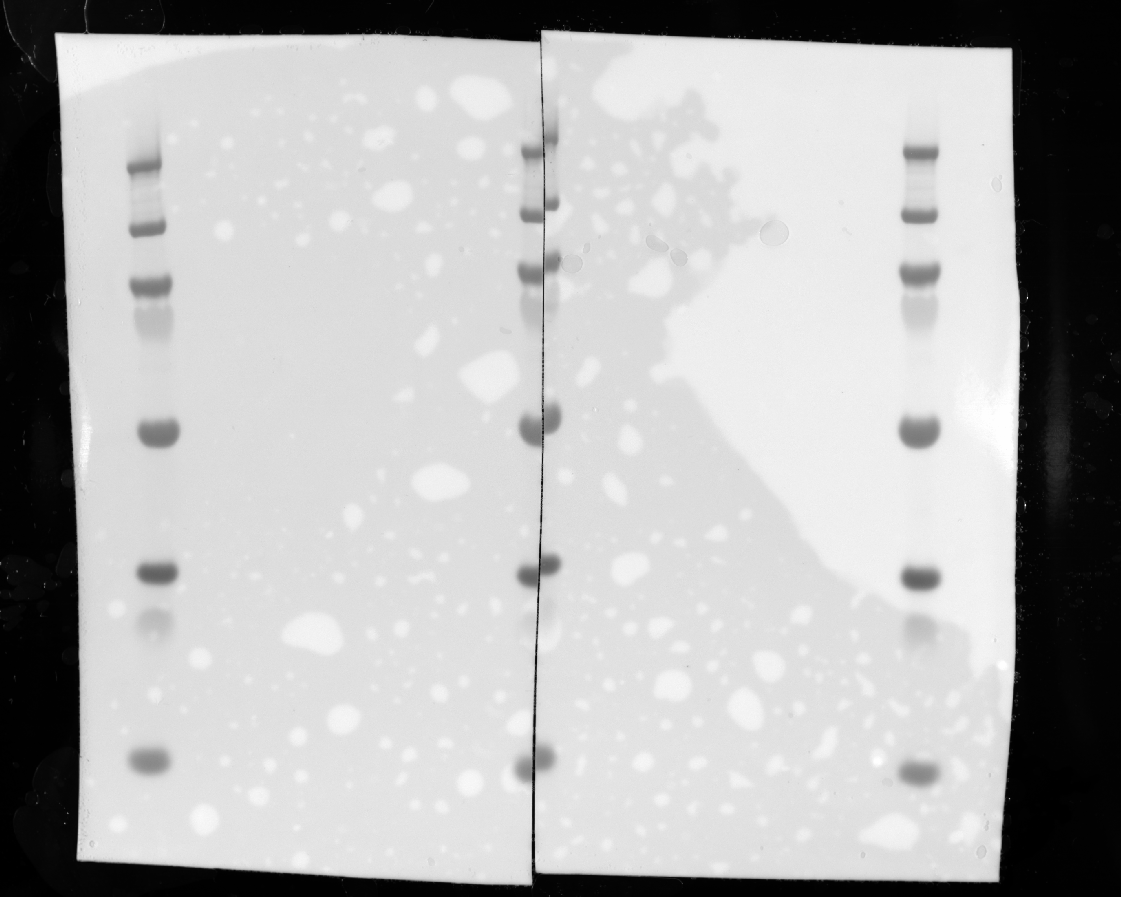

Supplement: Figure 4—figure supplement 1—source data 2. [file elife-105311-fig4-figsupp1-data2.zip › Figure 4 - Figure Supplement 1 - Source Data 2/Fig4_sup1D_marker_for_p_eif2alpha_2024-05-13 16h56m13s 0.168s.tif]
