# Supplementary material for: Human eIF2A has a minimal role in translation initiation and in uORF-mediated translational control in HeLa cells
Source: eLife. 2025 Jul 2;14:RP105311. doi: 10.7554/eLife.105311 (PMC12221301; doi:10.7554/eLife.105311)

Fig1 Suppl. Figure 1

C

the membrane was stripped
and re-bloted with anti-CMYC
and ATF-4 antibodies

the membrane was stripped
and re-bloted with
anti-tubulin antibodies

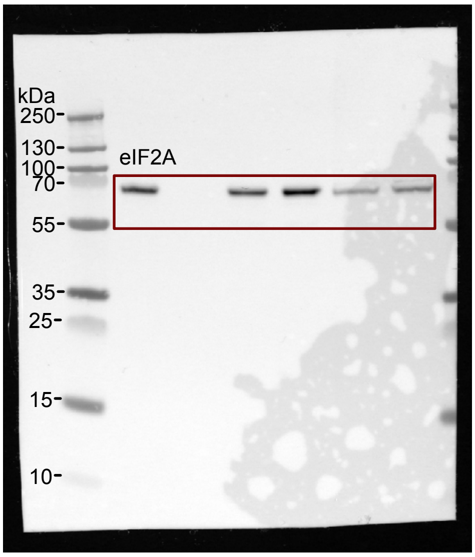

merge with ladder

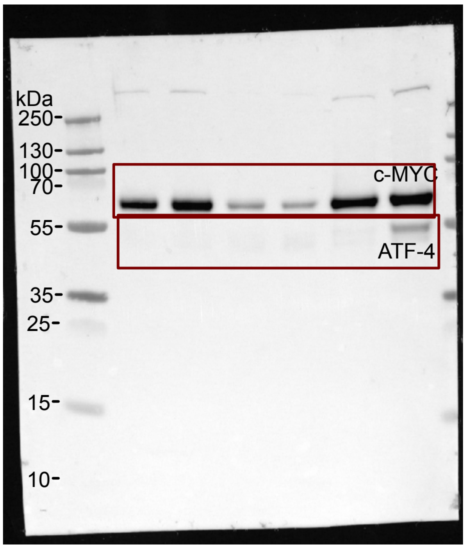

merge with ladder

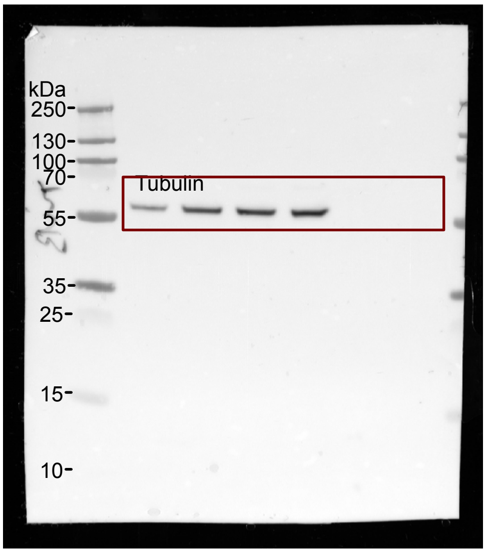

merge with ladder

F

same samples were run in a parallel:

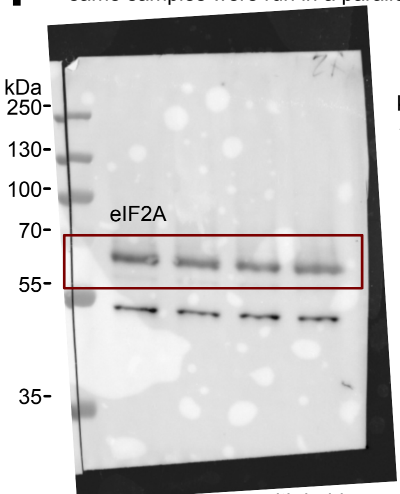

merge with ladder

the membrane was stripped
and re-bloted with
anti-HSP90 antibodies

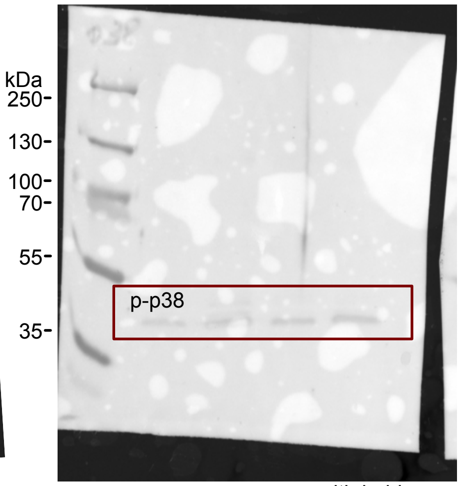

merge with ladder

the membrane was stripped cut
and re-bloted with
anti-Lamin and GAPDH antibodies

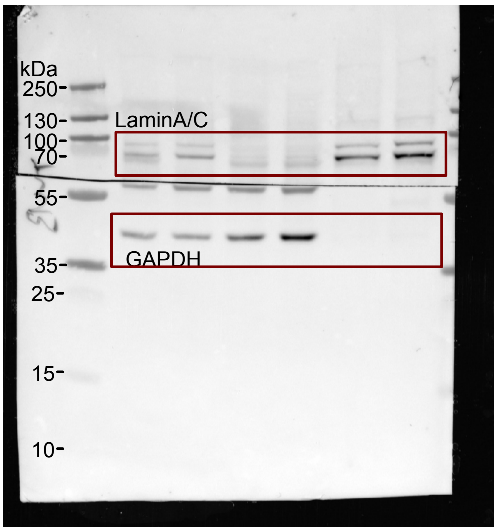

merge with ladder

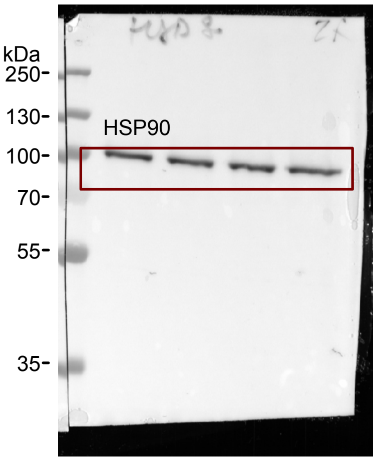

Supplement: Figure 1—figure supplement 1—source data 1. [file elife-105311-fig1-figsupp1-data1.pdf]
