# Supplementary material for: Human eIF2A has a minimal role in translation initiation and in uORF-mediated translational control in HeLa cells
Source: eLife. 2025 Jul 2;14:RP105311. doi: 10.7554/eLife.105311 (PMC12221301; doi:10.7554/eLife.105311)
Supplement: Figure 2—figure supplement 2—source data 1. [file elife-105311-fig2-figsupp2-data1.pdf]

Fig2 Suppl. Figure 2B

the area of interest was directly incubated with both eIF2A and tubulin antibodies:

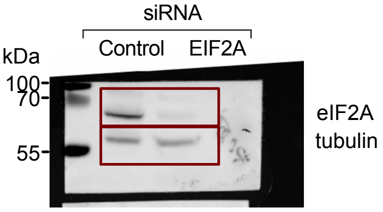

merge with ladder
